# Discovery of Novel and Selective G-Protein Coupled Receptor 120 (GPR120) Agonists for the Treatment of Type 2 Diabetes Mellitus

**DOI:** 10.3390/molecules27249018

**Published:** 2022-12-17

**Authors:** Xuekun Wang, Xu Li, Shiting Wei, Min Wang, Yao Xu, Weidi Hu, Zhenzhen Gao, Renmin Liu, Shiben Wang, Guoxia Ji

**Affiliations:** School of Pharmaceutical Sciences, Liaocheng University, 1 Hunan Street, Liaocheng 252059, China

**Keywords:** GPR120 agonists, design and synthesis, type 2 diabetes mellitus

## Abstract

Diabetes mellitus (DM), a chronic metabolic disorder characterized by high blood glucose, not only poses a serious threat to human life and health, but also places an economic burden on society. Currently available antidiabetic pharmacological agents have some adverse effects, which have stimulated researchers to explore novel antidiabetic agents with different mechanisms of action. G-protein Coupled Receptor 120 (GPR120), also known as free fatty acid receptor 4 (FFAR4), which is activated by medium-chain and long-chain fatty acids, has emerged as an interesting potential target for the treatment of metabolic disorders. Herein, we designed and synthesized a series of novel GPR120 agonists based on the structure of TUG-891, which is susceptible to *β*-oxidation and loses its GPR120 agonistic activity in vivo. Among the designed compounds, **14d** showed excellent agonistic activity and selectivity and could improve glucose tolerance in normal mice in a dose-dependent manner. In addition, the compound **14d** displayed good antidiabetic effects in diet-induced obese (DIO) mice and elevated insulin levels. Molecular simulations illustrated that compound **14d** could enter the active site of GPR120 and interact with ARG99, which plays an important role in GPR120 activation. Based on these observations, compound **14d** may be a promising lead compound deserving of further biological evaluation and structural modifications.

## 1. Introduction

Diabetes mellitus (DM), a chronic metabolic disorder characterized by high blood glucose levels, not only poses a serious threat to human life and health, but also places an economic burden on society. According to the latest data from the International Diabetes Federation, approximately 537 million adults (20–79 years) worldwide have diabetes, and this number is projected to increase to 643 million by 2030 and to 783 million by 2045. In parallel, the total global health expenditure on DM is estimated at USD 966 billion [1]. Several types of drugs that reduce blood glucose levels (insulin secretagogues, insulin sensitizers, α-glucosidase inhibitors, glucagon-like peptide-1 analogs, and dipeptidyl peptidase-4 inhibitors) have been developed for treating DM. However, currently available antidiabetic agents have some adverse effects, such as hypoglycemia, weight gain, gastrointestinal discomfort (disorder), nausea, diarrhea, liver function disorder, jaundice, and heart failure, which have prompted researchers to explore novel antidiabetic agents with different mechanisms of action [2].

Free fatty acid receptors (FFARs), such as GPR40 (also known as FFAR1), GPR41 (also known as FFAR3), GPR43 (also known as FFAR2), GPR119, and GPR120 (also known as FFAR4), which are activated by free fatty acids (FFAs), play key roles in regulating various physiological responses, including insulin secretion [3]. Several studies have reported that GPR41 and GPR43 are activated by short-chain FFAs [4,5,6], GPR40 is activated by medium- and long-chain FFAs [7,8], and GPR120 is activated by long-chain FFAs [9,10]. GPR120 is abundantly expressed in the intestines, lungs, adipose tissue, and pro-inflammatory macrophages and can stimulate glucagon-like peptide-1 (GLP-1) release [9,11,12]. GLP-1, a 36 amino acid peptide hormone secreted by endocrine L-cells in the small intestine and colon, plays an essential role in blood glucose regulation. In the pancreas, GLP-1 stimulates insulin secretion from *β*-cells in a blood glucose-dependent manner and inhibits glucagon secretion from α-cells [13,14]. Therefore, selective GPR120 agonists have therapeutic potential for the treatment of metabolic diseases, such as obesity and type 2 diabetes mellitus (T2DM).

**Figure 1:**
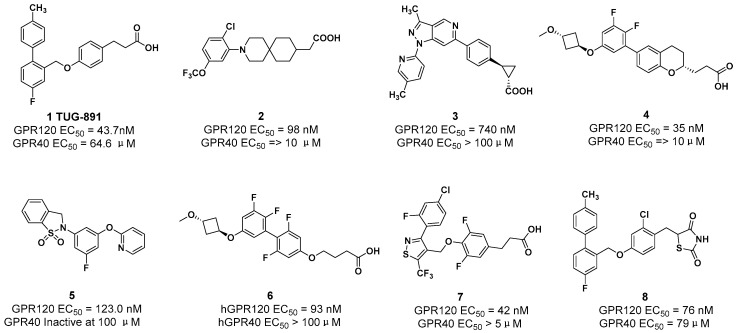
Representative GPR120 agonists.

**Figure 2:**
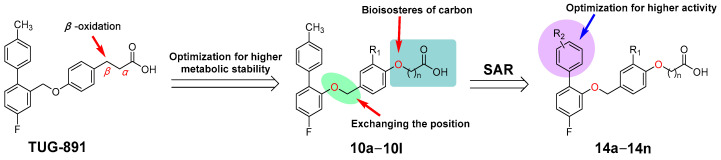
The design of phenoxyalkanoic acid derivatives.

Only a few GPR120 agonists have been reported in the literature, and no GPR120 agonists have entered clinical trials (Figure 1) [15,16,17,18,19,20,21,22,23,24,25,26]. Further research is needed to discover potent, selective, and orally bioavailable small molecule GPR120 agonists. TUG-891, with an EC_50_ value of 43.7 nM against human GPR120, is the first GPR120 agonist with high selectivity and activity, and has been widely used to explore the physiological function of GPR120 [15,27]. However, TUG-891 displayed high plasma clearance and a short half-life in vivo, possibly because phenylpropanoic acid moiety can undergo *β*-oxidation to generate benzoic acid derivatives. Therefore, we sought ways to modify the structure of TUG-891 to improve its pharmacokinetic profile, while retaining or improving GPR120 potency and selectivity. Based on a structure–activity relationship (SAR) study of TUG-891, our strategy to accomplish this goal was to replace the *β*-carbon with oxygen, which is the bio-isostere of carbon (Figure 2).

In this study, we first designed compounds **10a**–**10l** by replacing the *β*-carbon with oxygen, exchanging the position of carbon and oxygen, introducing substituents on the benzene ring, and prolonging the distance between the carboxylic acid group and benzene ring to improve the metabolic stability of the target compounds. After GPR120 agonistic activity and selectivity evaluation in vitro, compounds with good activity and selectivity were selected as lead compounds for SAR studies (**14a**–**14n**) by introducing substituents on the terminal benzene ring (Figure 2). Compound **14d**, with its excellent agonistic activity and selectivity in vitro, was suitable for further development. The results in rodents showed that compound **14d** had low clearance and long half-life and could improve glucose tolerance in normal mice in a dose-dependent manner, decreasing blood glucose levels in diet-induced obese (DIO) mice. These results indicated that compound **14d** may be a promising lead compound deserving of further biological evaluation and structural modifications.

## 2. Results and Discussion

### 2.1. Chemistry

**Scheme 1:**
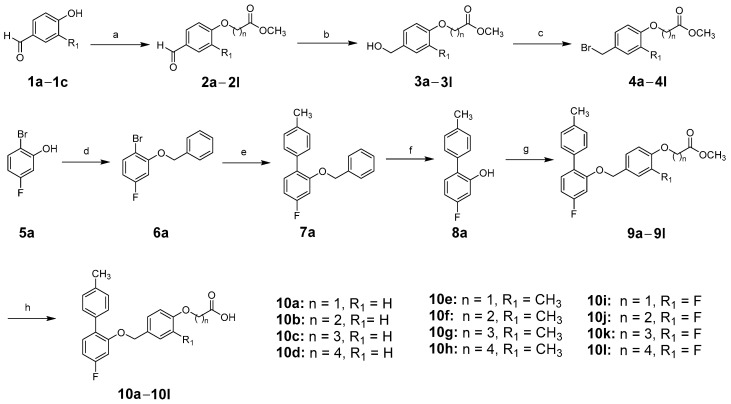
Reagents and conditions: Synthesis of target compounds **10a**–**10l**. (**a**) K_2_CO_3_, DMF, rt., 12 h, 62–78%; (**b**) NaBH_4_, CH_3_OH, THF, 0 °C, 2 h; (**c**) PBr_3_, CH_2_Cl_2_, 0 °C, 2 h, 48–56% (two steps); (**d**) K_2_CO_3_, acetone, 50 °C, 12 h, 82%; (**e**) 4-Tolylboronic acid, Pd(PPh_3_)_4_, Na_2_CO_3_, toluene, ethanol, H_2_O, 80 °C, overnight, 75%; (**f**) H_2_, Pd-C, rt., 12 h, 77%; (**g**) K_2_CO_3_, DMF, rt., 12 h; (**h**) NaOH, H_2_O, CH_3_OH, THF, 2 h, 51–58% (two steps).

Target compounds **10a**–**10l** were obtained according to the synthetic route summarized in Scheme 1. Substituted or unsubstituted *p*-hydroxybenzaldehyde **1a**–**1c** were reacted with commercially available halogenated hydrocarbons in the presence of potassium carbonate using the Williamson reaction to produce the corresponding aldehydes **2a**–**2l** in 62–78% yield. Aldehydes **2a**–**2l** were reduced by NaBH_4_ to give the corresponding alcohols **3a**–**3l**, which were then converted to benzyl bromides **4a**–**4l** by substituting the hydroxyl with PBr_3_ in 48–56% yield in two steps. Coupling of 2-bromo-5-fluorophenol **5a** with benzyl bromide in the presence of K_2_CO_3_ in refluxing acetone to produce **6a** in 82% yield. Suzuki coupling of **6a** and 4-tolylboronic acid by treatment with Pd(PPh_3_)_4_ and Na_2_CO_3_ produced arylated adduct **7a** in 75% yield, which was then deprotected with H_2_/Pa-C to produce phenol **8a** in 77% yield. Benzyl bromides **4a**–**4l** were coupled with phenol **8a** in the presence of K_2_CO_3_, followed by hydrolysis of the esters to produce the target compounds **10a**–**10l** in 51–58% yield in two steps. The structures of the target compounds **10a**–**10l** were confirmed using ^1^H-NMR, ^13^C-NMR, and high resolution mass spectrometry (HRMS).

**Scheme 2:**
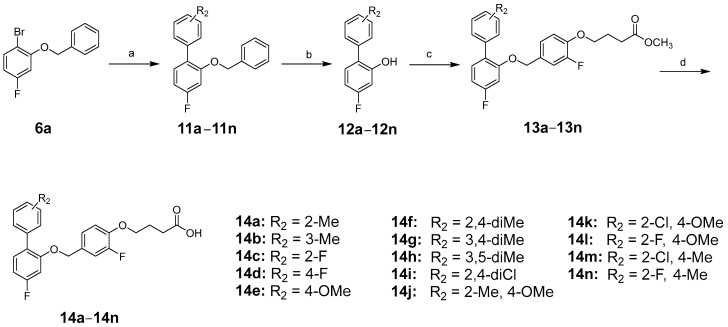
Reagents and conditions: Synthesis of target compounds **14a**–**14n**. (**a**) Pd(PPh_3_)_4_, Na_2_CO_3_, toluene, ethanol, H_2_O, 80 °C, overnight, 70–76%; (**b**) H_2_, Pd-C, rt., 12 h, 66–78%; (**c**) K_2_CO_3_, DMF, rt., 12 h, 73–85%; (**d**) NaOH, H_2_O, CH_3_OH, THF, 2 h, 53–62% (two steps).

Compound **10k**, a fluorine-containing phenoxybutyric acid derivative, exhibited excellent GPR120 agonist activity and selectivity in in vitro screening. Based on the structure of **10k**, fluorine-containing phenoxybutyric acid derivatives **14a**–**14n** were designed by introducing substituents on the terminal benzene ring. Target compounds **14a**–**14n** were obtained according to the synthetic route summarized in Scheme 2. Suzuki coupling of **6a** and various phenylboronic acids by treatment with Pd(PPh_3_)_4_ and Na_2_CO_3_ produced the corresponding arylated adducts **11a**–**11n** in 70–76% yield, which were then deprotected by H_2_/Pa-C to produce phenols **12a**–**12n** in 66–78% yield. The coupling of phenols **12a**–**12n** with methyl 4-(4-(bromomethyl)-2-fluorophenoxy)butanoate under Williamson ether conditions produced esters **13a**–**13n**, which were used in the next step without further purification. The esters **13a**–1**3n** were hydrolyzed by sodium hydroxide to give the target compounds **14a**–**14n** in 53–62% yield in two steps. The structures of the target compounds **14a**–**14n** were confirmed using ^1^H-NMR, ^13^C-NMR, and HRMS.

### 2.2. Pharmacology

#### 2.2.1. GPR120 Agonistic Activity and Selectivity

Compounds were screened against human GPR120 (hGPR120) transfected CHO cells using a calcium flux assay and TUG-891 was used as a positive control. We began our SAR studies by modifying the phenylpropionic acid moiety. Compound **10a**, obtained by replacing the *β*-carbon atom of phenylpropionic acid with an oxygen atom and exchanging carbon and oxygen between benzene rings, maintained the agonistic activity of TUG-891. Compound **10b** was obtained by extending the distance between the carboxyl group and the oxygen atom (n = 2), resulting in a significant decrease in agonistic activity, while compounds **10c** (n = 3) and **10d** (n = 4) slightly increased the agonistic activity compared to **10b**. The introduction of an electron-donating methyl group at the ortho position of the benzene ring (**10e**) slightly reduced the agonistic activity compared to **10a**, and extending the distance between carboxyl and oxygen further decreased the activity (**10f**, **10g**, **10h** vs. **10e**). Introduction of the electron-withdrawing group fluorine at the ortho position of benzene ring had a beneficial effect on hGPR120 activity, as indicated by EC_50_ values of 77.2, 112.2, 57.6 and 96.8 nM for **10i**, **10j**, **10k** and **10l**, respectively, in the calcium flux assay. Compounds with good hGPR120 agonistic activity (EC_50_ < 0.2 μM) were examined for human GPR40 (hGPR40) agonistic activity. The results revealed that the EC_50_ of the compounds to hGPR40 was >70 µM, indicating that the compounds had excellent selectivity (Table 1). This study aimed to improve the metabolic stability of TUG-891 cells in vivo. The pharmacokinetics of compound **10k** were determined in normal C57BL/6 mice, and compound **10k** exhibited higher metabolic stability than TUG-891 (Figure S1, see Supporting Materials). Compound **10k**, which exhibited potent hGPR120 activity, selectivity and metabolic stability, was selected for further optimization.

Compounds **14a**–**14n** were designed based on the phenoxybutanoic acid head of compound **10k** by altering the substituents on the terminal benzene ring. The monomethyl substitution of the terminal benzene ring, whether at the 2-position or the 3-position, maintained the agonistic activity of compound **10k** (EC_50_ values of 90.4 and 82.5 nM for **14a** and **14b**, respectively). Substitution of the 4-methyl group with fluorine significantly increased the agonistic activity, whereas that with 2-fluorine decreased the agonistic activity (EC_50_ values of 168.5 and 37.5 nM for **14c** and **14d**, respectively). Compound **14e** with a methoxy group at the 4-position was fourfold less potent against hGRP120 than 4-methyl-substituted analog **10k**, and the introduction of a methyl group (**14f**), chlorine(**14g**), or fluorine (**14h**) atom at the 2-position did not increase the agonistic activity. Compounds substituted by two of the same groups on the terminal benzene ring showed comparable agonistic activity, while the effects were weaker than that of compound **10k** (EC_50_ values of 158.6, 150.3, 125.6, and 136.5 nM for **14i**, **14j**, **14k**, and **14l**, respectively). The introduction of an electron-withdrawing group at the 2-position of compound **10k** could also reduce the agonistic activity (EC_50_ values of 187.3 and 312.7 nM for **14m** and **14m**, respectively). All results indicated that the disubstitution of the terminal benzene ring was detrimental to the agonistic activity. Compounds with good hGPR120 agonistic activity (EC_50_ < 0.2 μM) were examined for hGPR40 agonistic activity. The results revealed that the EC_50_ of the compounds to hGPR40 was >60 µM, indicating that the compounds had excellent selectivity (Table 2). The in vitro activity of compound **14d** was examined on CHO cells expressing mouse GPR120 (mGPR120) and mouse GPR40 (mGPR40). The results indicated that the agonistic activity of compound **14d** was slightly reduced against mGPR120 (EC_50_ = 83.2 nM), while the agonistic activity against mGPR40 was slightly increased (EC_50_ = 12.7 μM). As a result, the selectivity of compound **14d** was somewhat reduced (Table 3). The dose-dependent curves of compound **14d** and TUG-891 for hGPR120 and mGPR120 indicated that compound **14d** was a full agonist just like TUG-891 (Figure S2 and Table S1, see Supporting Materials). Compound **14d**, which exhibited potent GPR120 activity and 152.6-fold selectivity for mGPR120 over mGPR40, was selected for further evaluation in vivo.

#### 2.2.2. Pharmacokinetic Evaluation of Compounds **10k** and **14d**

The pharmacokinetic profiles of **10k** and **14d** were obtained from C57BL/6 mice. The results showed that compounds **10k** and **14d** displayed excellent metabolic stability. Compound **10k** obtained by replacing phenylpropionic acid with phenoxybutyric acid reached *C*_max_ after 30 min of gavage administration at a dose of 10 mg/kg and exhibited better pharmacokinetic properties than TUG-891, indicating that 2-fluoro-substituted phenoxybutyric acid has a metabolically stable chemical structure. The 2-fluoro-substituted phenoxybutyric acid analog **14d** displayed a favorable mouse pharmacokinetic profile, characterized by a higher maximum plasma concentration, longer half-life, and higher exposure, indicating that compound **14d** had a suitable profile to investigate the in vivo effects (Table 4). The pharmacokinetic profiles traces were shown in the Supplementary Materials (Figure S1, see Supporting Materials).

#### 2.2.3. Oral Glucose Tolerance Test (OGTT) of Compound **14d** in Normal C57BL/6 Mice

**Figure 3:**
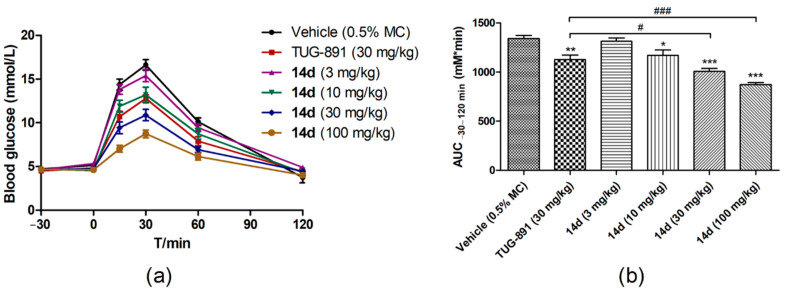
Effects of compound **14d** and TUG-891 on plasma glucose levels during the OGTT in fasting C57BL/6 mice. (**a**) Time-dependent changes of blood glucose levels after oral administration of compound **14d**, followed by the oral glucose load (3 g/kg). (**b**) AUC_−30–120 min_ of plasma glucose levels. Values are expressed as the mean ± SD (*n* = 8). * *p* ≤ 0.05 compared with vehicle-treated C57BL/6 mice by Student’s *t* test; ** *p* ≤ 0.01 compared with vehicle-treated C57BL/6 mice by Student’s *t* test; *** *p* ≤ 0.001 compared with vehicle-treated C57BL/6 mice by Student’s *t* test; ^#^ *p* ≤ 0.05 compared with TUG-891-treated C57BL/6 mice by Student’s *t* test; ^###^ *p* ≤ 0.001 compared with TUG-891-treated C57BL/6 mice by Student’s *t* test.

GPR120 agonists have been reported to increase GLP-1 secretion and improve glucose tolerance in vivo [28]. Thus, the glucose tolerance effect of compound **14d** was evaluated in C57BL/6 mice, and TUG-891 was selected as a positive control. Compound **14d** was orally administered at doses of 3, 10, 30, and 100 mg/kg, 30 min before the oral glucose challenge at a dose volume of 3 g/kg. Glucose levels were measured from 30 min before glucose challenge to 120 min after the glucose challenge. The blood glucose levels reached a maximum after 30 min of the glucose challenge, and compound **14d** exhibited a significant reduction in blood glucose at a dose of 30 mg/kg, which was superior to that of TUG-891. Furthermore, compound **14d** showed a dose-dependent area for lowering glucose and overall lowering of glucose under the curve (AUC). Compound **14d** at 30 mg/kg reduced plasma glucose levels with an AUC of 25% (*p* < 0.001) after 120 min compared with glucose alone, and no hypoglycemia was observed during the experiment (Figure 3).

#### 2.2.4. Anti-Hyperglycemic Effects of Compound **14d** in DIO Mice

**Figure 4:**
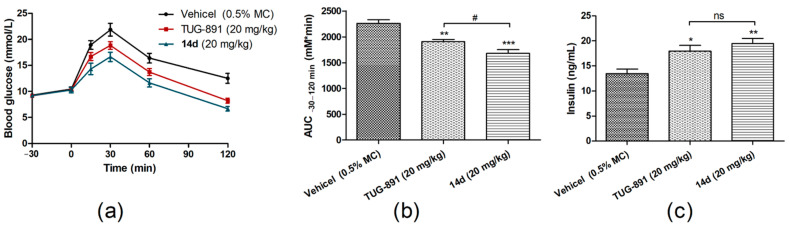
Effects of compound **14d** and TUG-891 on plasma glucose levels during the OGTT in fasting DIO mice. (**a**) The changes of blood glucose levels after oral administration of compound **14d**, followed by the oral glucose load (2 g/kg); (**b**) AUC_−30–120 min_ of plasma glucose levels. (**c**) Insulin levels at 30 min after the glucose load. Values are expressed as the mean ± SD (*n* = 6). * *p* ≤ 0.05 compared with vehicle-treated DIO mice by Student’s *t* test; ** *p* ≤ 0.01 compared with vehicle-treated DIO mice by Student’s *t* test; *** *p* ≤ 0.001 compared with vehicle-treated DIO mice by Student’s *t* test; ^#^ *p* ≤ 0.05 compared with TUG-891-treated DIO mice by Student’s *t* test; ns means no significant.

Thereafter, the anti-hyperglycemic and insulinotropic effects of compound **14d** were investigated in DIO mice. Compound **14d** and TUG-891 were orally administered at a dose of 20 mg/kg 30 min before the oral glucose challenge at a dose volume of 2 g/kg. The results showed that the glucose AUC_0–120 min_ of compound **14d** and TUG-891-treated DIO mice were significantly lower than that of vehicle-treated mice, and compound **14d** was superior to the positive control TUG-891. Additionally, insulin concentration was also tested 30 min after the glucose challenge; **14d**-treated mice showed significantly increased insulin levels compared to vehicle-treated mice, which was better than TUG-891 (Figure 4). These results indicated that compound **14d** could exert antidiabetic effects by promoting insulin secretion.

#### 2.2.5. Molecular Modeling

**Figure 5:**
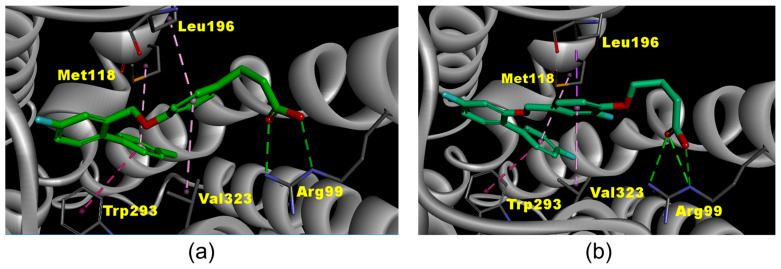
(**a**) Docking of compound **14d** inside a homology GPR120 model. (**b**) Docking of TUG-891 inside a homology GPR120 model.

Molecular docking studies were performed to explore the mode of binding of compound **14d** to the GPR120 receptor. As the crystal structure of GPR120 has not been obtained experimentally, we first constructed the protein structure of GPR120 with homology modeling. To ensure the accuracy of homology modeling, the crystal structures of turkey β1 adrenoceptor 20 (PDB code 6IBL) [29], neurotensin receptor21 (PDB code 4XES) [30], and β2-adrenoceptor (PDB code 3P0G) [31] with good homology were selected as templates. The model structure with the lowest discrete optimized protein energy (DOPE) score was optimized and evaluated, and used for molecular docking. The docking results showed that compound **14d** interacted with GPR120 in a manner similar to that of TUG-891. Compound **14d** also bound excellently to the active site of GPR120 and formed hydrogen-bonding interactions with ARG99, which played a vital role in stabilizing its binding mode [32,33]. Additionally, the ring moieties of **14d** exhibited several π-π and π-alkyl interactions with pocket amino acids of the GPR120 receptor, which was advantageous for stabilizing the complex formed by **14d** and GPR120 (Figure 5). The docking results further indicated that compound **14d** regulated blood glucose through agonist of GPR120.

## 3. Materials and Methods

### 3.1. Synthesis

All commercially available materials and reagents were used without purification unless otherwise indicated. Purification via column chromatography was performed using silica gel (200–300 mesh). The melting points of the target compounds **10a**–**10l** and **14a**–**14n** were determined using an x-5 micro melting point apparatus, which was uncorrected. The purity and characterization of the target compounds were established using a combination of high-performance liquid chromatography and NMR analytical techniques, and the purity was >95% for all test compounds. NMR spectra (500 MHz for ^1^H NMR and 125 MHz for ^13^C NMR spectra) were recorded on a Bruker AVANCE NEO 500 instrument, and were to be determined in CDCl_3_ or DMSO-*d_6_*. Chemical shifts were reported in ppm relative to tetramethylsilane (0.00 ppm) or solvent peaks as the internal reference. Splitting patterns are indicated as follows: s, singlet; d, doublet; t, triplet; m, multiplet. Coupling constants (*J* values) are given in hertz (Hz). High resolution mass spectrometry was conducted using a UPLC G2-XS QTOF spectrometer (Waters) with the electrospray ionization Fourier transform ion cyclotron resonance technique. The NMR and HRMS spectra of compounds **10a**–**10l** and **14a**-**14n** are presented in Figures S3–S80.

#### 3.1.1. General Synthetic Procedure for Intermediates **2a**–**2l**

A mixture of *p*-hydroxybenzaldehyde derivatives **1a**–**1c** (10.0 mmol), methyl bromoacetate, methyl bromopropionate, methyl bromobutyrate, or methyl bromovalerate (10.0 mmol), and K_2_CO_3_ (20.0 mmol) in DMF (15 mL) was stirred at room temperature for 12 h. The reaction was quenched with H_2_O (30 mL), and extracted with ethyl acetate (3 × 30 mL). The combined organic layer was washed with H_2_O (4 × 50 mL), and DMF dissolved in water was separated. The organic layer was dried over anhydrous Na_2_SO_4_, concentrated with rotary evaporation, and purified with silica gel column chromatography to obtain the desired compounds **2a**–**2l**.

#### 3.1.2. General Synthetic Procedure for Intermediates **3a**–**3l**

To a solution of aldehyde derivatives **2a**–**2l** (5.0 mmol) in THF (20 mL) and MeOH (15 mL) was added borohydride (5.0 mmol) portion wise at 0 °C. The mixture was stirred at 0 °C for 1 h and quenched with 1 N HCl after completion of the reaction (TLC examination). The mixture was extracted with ethyl acetate (3 × 30 mL), and organic layers were combined and washed with H_2_O (2 × 30 mL) and saturated brine (2 × 30 mL) prior to drying over anhydrous Na_2_SO_4_. After filtration and concentration using a rotary evaporator under reduced pressure, a residue of **3a**–**3l** was obtained, which was used in the next step without further purification.

#### 3.1.3. General Synthetic Procedure for Intermediates **4a**–**4l**

To the crude intermediates **4a**–**4l** (5.0 mmol) in dichloromethane (30 mL) was added phosphorus tribromide (2.5 mmol) dissolved in dichloromethane (5 mL) at −5 °C. The mixture was stirred at 0 °C for 1 h and quenched with cold water (20 mL) after the reaction completion (TLC examination). The mixture was stirred for 2 h at room temperature and extracted with dichloromethane (3 × 20 mL). The combined organic layers were washed with saturated brine (2 × 30 mL) and dried over anhydrous Na_2_SO_4_. After filtration and concentration using a rotary evaporator under reduced pressure, the residue was purified with silica gel column chromatography to obtain the intermediates **4a**–**4l**.

#### 3.1.4. Synthetic Procedure for Intermediate 4-fluoro-4′-methyl-[1,1′-biphenyl]-2-ol (**8a**)

A mixture of 2-bromo-5-fluorophenol **5a** (10.0 mmol), benzyl bromide (10.0 mmol), and K_2_CO_3_ (20.0 mmol) in acetone (30 mL) was stirred at 60 °C for 12 h. The reaction was cooled, and the insoluble material was filtered after the reaction completion (TLC examination). The filtrate was evaporated under reduced pressure and the residues was purified with silica gel column chromatography to obtain the intermediate **6a** (2.3 g, 82%).

Intermediate **6a** (5.0 mmol) and 4-tolylboronic acids (5.85 mmol) were dissolved in a mixture of 1 N sodium carbonate aq. (20 mL), ethanol (10 mL), and toluene (20 mL). After nitrogen substitution, Pd(PPh_3_)_4_ (0.5 mmol) was added as a catalyst. The reaction mixture was stirred at 80 °C under a nitrogen atmosphere for 12 h. After the reaction was complete (TLC examination), the reaction mixture was cooled, and diluted with ethyl acetate (30 mL). The insoluble material of the mixture was filtered off through celite. The organic layer of the filtrate was washed with water (2 × 30 mL) and brine (2 × 30 mL), dried over anhydrous Na_2_SO_4_, and concentrated under reduced pressure. The residue was purified with silica gel column chromatography to afford the product **7a** (1.1 g, 75%) as a solid.

To a solution of **7a** (2.5 mmol) in methanol was add Pd-C (0.25 mmol) as a catalyst, and the mixture was stirred under hydrogen atmosphere at room temperature for 24 h. After the reaction was complete (TLC examination), the insoluble material of the mixture was filtered off through celite. The filtrate was evaporated under reduced pressure and the residues was purified with silica gel column chromatography to obtain the intermediate **8a** (0.39 g, 77%) as a solid. ^1^H NMR (500 MHz, CDCl_3_) 7.33 (d, *J* = 7.8 Hz, 2H), 7.29–7.25 (m, 1H), 7.14 (d, *J* = 7.7 Hz, 2H), 7.07 (dd, *J* = 11.4, 2.3 Hz, 1H), 6.81 (td, *J* = 8.3, 2.3 Hz, 1H), 2.30 (s, 2H).

#### 3.1.5. General Synthetic Procedure for Target Compounds **10a**–**10l**

A mixture of **8a** (1.0 mmol), intermediates **4a**–**4l** (1.0 mmol), and K_2_CO_3_ (2.0 mmol) in acetone (15 mL) was stirred at 60 °C for 12 h. The reaction was cooled, and the insoluble material was filtered after the reaction completion (TLC examination). The filtrate was evaporated under reduced pressure and the residues **9a**–**9l** were used in the next step without further purification.

To a solution of comprising compound **9a**–**9l** (1.0 mmol) in THF (10 mL), CH_3_OH (5 mL), and H_2_O (5 mL) was added NaOH solution (2 N, 2.0 mmol) at room temperature. The reaction mixture was stirred for 2 h and acidified with HCl (1 N) to a pH of 3 after hydrolysis was complete (TLC examination). The mixture was extracted with ethyl acetate (3 × 20 mL) and the combined organic layers were washed with H_2_O (2 × 30 mL) and saturated brine (2 × 30 mL). The organic layer was dried over anhydrous Na_2_SO_4_, concentrated by rotary evaporation, and purified with silica gel column chromatography to obtain the target compounds **10a**–**10l**.

*2-(4-(((4-fluoro-4′-methyl-[1,1′-biphenyl]-2-yl)oxy)methyl)phenoxy)acetic acid* (**10a**): Colorless solid 0.20 g, yield 53% of two steps; m.p. 164~166 °C; ^1^H NMR (500 MHz, DMSO-*d_6_*) δ 13.01 (s, 1H), 7.36 (d, *J* = 7.7 Hz, 2H), 7.34–7.26 (m, 4H), 7.18 (d, *J* = 7.6 Hz, 2H), 7.09 (d, *J* = 11.3 Hz, 1H), 6.89 (d, *J* = 8.2 Hz, 2H), 6.84 (t, *J* = 8.2 Hz, 1H), 5.05 (s, 2H), 4.66 (s, 2H), 2.31 (s, 3H); ^13^C NMR (125 MHz, DMSO-*d_6_*) δ 170.60, 163.59, 157.92, 156.82, 136.53, 134.84, 131.86, 129.56, 129.06, 126.94, 114.83, 107.69, 101.78, 70.12, 64.97, 21.18; HRMS (ES^+^) for C_22_H_19_FO_4_ (M + Na)^+^: calcd 389.1165; found, 389.1161.

*3-(4-(((4-fluoro-4′-methyl-[1,1′-biphenyl]-2-yl)oxy)methyl)phenoxy)propanoic acid* (**10b**): Colorless solid 0.19 g, yield 51% of two steps; m.p. 120~122 °C; ^1^H NMR (500 MHz, DMSO-*d_6_*) δ 12.37 (s, 1H), 7.36 (d, *J* = 8.0 Hz, 2H), 7.33–7.26 (m, 3H), 7.17 (d, *J* = 7.9 Hz, 2H), 7.09 (dd, *J* = 11.5, 2.4 Hz, 1H), 6.91 (d, *J* = 8.6 Hz, 2H), 6.84 (td, *J* = 8.4, 2.4 Hz, 1H), 5.05 (s, 1H), 4.16 (t, *J* = 6.0 Hz, 1H), 2.68 (t, *J* = 6.0 Hz, 1H), 2.31 (s, 2H); ^13^C NMR (125 MHz, DMSO-*d_6_*) δ 172.69, 163.60, 161.67, 158.52, 156.85, 136.53, 134.84, 131.83, 129.69, 129.07, 126.92, 114.78, 107.50, 101.76, 101.56, 70.19, 64.06, 34.57, 21.17; HRMS (ES^+^) for C_23_H_21_FO_4_ (M + Na)^+^: calcd 403.1322; found, 403.1328.

*4-(4-(((4-fluoro-4′-methyl-[1,1′-biphenyl]-2-yl)oxy)methyl)phenoxy)butanoic acid* (**10c**): Colorless solid 0.22 g, yield 57% of two steps; m.p. 106~108 °C; ^1^H NMR (500 MHz, DMSO-*d_6_*) δ 12.13 (s, 1H), 7.36 (d, *J* = 8.0 Hz, 2H), 7.32–7.26 (m, 3H), 7.17 (d, *J* = 8.0 Hz, 2H), 7.09 (dd, *J* = 11.5, 2.4 Hz, 1H), 6.91 (d, *J* = 8.6 Hz, 2H), 6.84 (td, *J* = 8.4, 2.4 Hz, 1H), 5.05 (s, 2H), 3.97 (t, *J* = 6.4 Hz, 2H), 2.37 (t, *J* = 7.3 Hz, 2H), 2.31 (s, 3H), 1.98–1.89 (m, 2H); ^13^C NMR (125 MHz, DMSO-*d_6_*) δ 174.54, 163.59, 161.66, 158.69, 156.85, 136.52, 134.85, 131.83, 129.68, 129.04, 126.93, 114.80, 107.66, 107.50, 101.77, 70.21, 67.04, 30.58, 24.71, 21.17; HRMS (ES^+^) for C_24_H_23_FO_4_ (M + Na)^+^: calcd 417.1478; found, 417.1480.

*5-(4-(((4-fluoro-4′-methyl-[1,1′-biphenyl]-2-yl)oxy)methyl)phenoxy)pentanoic acid* (**10d**): Colorless solid 0.21 g, yield 52% of two steps; m.p. 124~126 °C; ^1^H NMR (500 MHz, DMSO-*d_6_*) δ 12.03 (s, 1H), 7.36 (d, *J* = 8.0 Hz, 2H), 7.33–7.26 (m, 3H), 7.17 (d, *J* = 8.0 Hz, 2H), 7.09 (dd, *J* = 11.5, 2.4 Hz, 1H), 6.90 (d, *J* = 8.6 Hz, 2H), 6.84 (td, *J* = 8.4, 2.4 Hz, 1H), 5.04 (s, 2H), 3.95 (t, *J* = 6.2 Hz, 2H), 2.31 (s, 3H), 2.28 (t, *J* = 7.3 Hz, 2H), 1.77–1.69 (m, 2H), 1.68–1.60 (m, 2H); ^13^C NMR (125 MHz, DMSO-*d_6_*) δ 174.82, 163.59, 161.66, 158.81, 156.85, 136.52, 134.86, 131.83, 129.67, 129.04, 128.80, 126.93, 114.78, 114.61, 107.65, 101.76, 70.22, 67.58, 33.76, 28.59, 21.69, 21.17; HRMS (ES^+^) for C_25_H_25_FO_4_ (M + Na)^+^: calcd 431.1635; found, 431.1637.

*2-(4-(((4-fluoro-4′-methyl-[1,1′-biphenyl]-2-yl)oxy)methyl)-2-methylphenoxy)acetic acid* (**10e**): Colorless solid 0.20 g, yield 52% of two steps; m.p. 128~130 °C; ^1^H NMR (500 MHz, DMSO-*d_6_*) δ 12.97 (s, 1H), 7.37 (d, *J* = 8.0 Hz, 2H), 7.29 (dd, *J* = 8.3, 7.2 Hz, 1H), 7.18 (d, *J* = 8.0 Hz, 2H), 7.14 (d, *J* = 10.1 Hz, 2H), 7.08 (dd, *J* = 11.4, 2.4 Hz, 1H), 6.84 (td, *J* = 8.4, 2.4 Hz, 1H), 6.79 (d, *J* = 8.2 Hz, 1H), 5.01 (s, 2H), 4.68 (s, 2H), 2.32 (s, 3H), 2.17 (s, 3H); ^13^C NMR (125 MHz, DMSO-*d_6_*) δ 170.71, 163.60, 161.66, 156.89, 156.02, 136.54, 134.87, 131.81, 130.60, 129.61, 129.07, 129.03, 127.03, 126.70, 126.34, 111.54, 107.71, 101.85, 70.27, 65.22, 21.18, 16.51; HRMS (ES^+^) for C_23_H_21_FO_4_ (M + Na)^+^: calcd 403.1322; found, 403.1328.

*3-(4-(((4-fluoro-4′-methyl-[1,1′-biphenyl]-2-yl)oxy)methyl)-2-methylphenoxy)propanoic acid* (**10f**): Colorless solid 0.20 g, yield 52% of two steps; m.p. 125~127 °C; ^1^H NMR (500 MHz, DMSO-*d_6_*) δ 12.34 (s, 1H), 7.37 (d, *J* = 7.5 Hz, 2H), 7.29 (t, *J* = 7.5 Hz, 1H), 7.23–7.12 (m, 4H), 7.08 (d, *J* = 10.9 Hz, 1H), 6.91 (d, *J* = 8.1 Hz, 1H), 6.84 (t, *J* = 7.6 Hz, 1H), 5.01 (s, 2H), 4.16 (t, *J* = 5.2 Hz, 2H), 2.69 (t, *J* = 5.0 Hz, 2H), 2.31 (s, 3H), 2.09 (s, 3H); ^13^C NMR (125 MHz, DMSO-*d_6_*) δ 172.75, 163.60, 161.66, 156.91, 156.59, 136.54, 134.86, 131.79, 130.51, 129.60, 129.02, 128.70, 127.01, 126.94, 126.25, 111.72, 107.69, 101.84, 70.35, 64.38, 34.75, 21.17, 16.27; HRMS (ES^+^) for C_24_H_23_FO_4_ (M + Na)^+^: calcd 417.1478; found, 417.1482.

*4-(4-(((4-fluoro-4′-methyl-[1,1′-biphenyl]-2-yl)oxy)methyl)-2-methylphenoxy)butanoic acid* (**10g**): Colorless solid 0.22 g, yield 55% of two steps; m.p. 99~101 °C; ^1^H NMR (500 MHz, DMSO-*d*_6_) δ 12.13 (s, 1H), 7.41–7.34 (m, 2H), 7.29 (dd, *J* = 8.5, 7.0 Hz, 1H), 7.18 (d, *J* = 7.8 Hz, 2H), 7.14 (d, *J* = 8.8 Hz, 2H), 7.08 (dd, *J* = 11.5, 2.5 Hz, 1H), 6.88 (d, *J* = 8.2 Hz, 1H), 6.84 (td, *J* = 8.4, 2.5 Hz, 1H), 5.01 (s, 2H), 3.97 (t, *J* = 6.2 Hz, 2H), 2.40 (t, *J* = 7.3 Hz, 2H), 2.32 (s, 3H), 2.12 (s, 3H), 2.00–1.91 (m, 2H); ^13^C NMR (125 MHz, DMSO-*d*_6_) δ 174.60, 156.79, 136.54, 134.87, 131.77, 130.53, 129.61, 129.03, 128.45, 126.98, 126.18, 111.41, 107.60, 101.74, 70.36, 67.11, 30.74, 24.82, 21.18, 16.40; HRMS (ES^+^) for C_25_H_25_FO_4_ (M + Na)^+^: calcd 431.1635; found, 431.1637.

*5-(4-(((4-fluoro-4′-methyl-[1,1′-biphenyl]-2-yl)oxy)methyl)-2-methylphenoxy)pentanoic acid* (**10h**): Colorless solid 0.24 g, yield 57% of two steps; m.p. 114~116 °C; ^1^H NMR (500 MHz, DMSO-*d_6_*) δ 12.03 (s, 1H), 7.36 (d, *J* = 8.0 Hz, 2H), 7.28 (dd, *J* = 8.3, 7.1 Hz, 1H), 7.18 (d, *J* = 7.9 Hz, 2H), 7.14 (d, *J* = 10.1 Hz, 2H), 7.08 (dd, *J* = 11.4, 2.4 Hz, 1H), 6.88 (d, *J* = 8.2 Hz, 1H), 6.83 (td, *J* = 8.4, 2.5 Hz, 1H), 5.01 (s, 2H), 3.95 (t, *J* = 6.1 Hz, 2H), 2.31 (s, 3H), 2.29 (t, *J* = 7.3 Hz, 2H), 2.12 (s, 3H), 1.78–1.70 (m, 2H), 1.70–1.63 (m, 2H); ^13^C NMR (125 MHz, DMSO-*d_6_*) δ 174.85, 163.59, 161.66, 156.92, 156.81, 136.53, 134.88, 131.79, 130.51, 129.60, 129.02, 128.35, 127.02, 126.94, 126.12, 111.45, 107.67, 101.84, 70.38, 67.66, 33.79, 28.70, 21.77, 21.17, 16.40; HRMS (ES^+^) for C_26_H_27_FO_4_ (M + Na)^+^: calcd 445.1791; found, 445.1792.

*2-(2-fluoro-4-(((4-fluoro-4′-methyl-[1,1′-biphenyl]-2-yl)oxy)methyl)phenoxy)acetic acid* (**10i**): Colorless solid 0.22 g, yield 58% of two steps; m.p. 90~92 °C; ^1^H NMR (500 MHz, DMSO-*d_6_*) δ 13.09 (s, 1H), 7.37 (d, *J* = 8.0 Hz, 2H), 7.32–7.28 (m, 1H), 7.26–7.17 (m, 3H), 7.12 (d, *J* = 8.7 Hz, 1H), 7.11–7.03 (m, 2H), 6.86 (td, *J* = 8.4, 2.3 Hz, 1H), 5.06 (s, 2H), 4.77 (s, 2H), 2.32 (s, 3H); ^13^C NMR (125 MHz, DMSO-*d_6_*) δ 170.22, 163.59, 161.65, 156.63, 152.67, 150.73, 145.72, 136.61, 134.78, 131.89, 130.53,129.59, 129.06, 127.03, 124.17, 115.94, 115.12, 107.89, 101.82, 69.52, 65.49, 21.18; HRMS (ES^+^) for C_22_H_18_F_2_O_4_ (M + Na)^+^: calcd 407.1071; found, 407.1072.

*3-(2-fluoro-4-(((4-fluoro-4′-methyl-[1,1′-biphenyl]-2-yl)oxy)methyl)phenoxy)propanoic acid* (**10g**): Colorless solid 0.21 g, yield 54% of two steps; m.p. 94~96 °C; ^1^H NMR (500 MHz, DMSO-*d_6_*) δ 12.43 (s, 1H), 7.37 (d, *J* = 7.9 Hz, 2H), 7.33–7.27 (m, 1H), 7.21 (d, *J* = 11.3 Hz, 2H), 7.16 (m, 3H), 7.09 (m, *J* = 11.3, 2.0 Hz, 1H), 6.86 (td, *J* = 8.4, 2.1 Hz, 1H), 5.06 (s, 2H), 4.23 (t, *J* = 6.0 Hz, 2H), 2.72 (t, *J* = 6.0 Hz, 2H), 2.32 (s, 3H); ^13^C NMR (125 MHz, DMSO-*d_6_*) δ 172.51, 163.59, 161.65, 156.63, 152.72, 150.78, 146.31, 136.61, 134.76, 131.87, 130.16, 129.59, 129.05, 126.98, 124.48, 115.89, 115.19, 107.86, 101.78, 69.55, 65.24, 34.46, 21.17; HRMS (ES^+^) for C_23_H_20_F_2_O_4_ (M + Na)^+^: calcd 421.1227; found, 421.1233.

*4-(2-fluoro-4-(((4-fluoro-4′-methyl-[1,1′-biphenyl]-2-yl)oxy)methyl)phenoxy)butanoic acid* (**10k**): Colorless solid 0.23 g, yield 57% of two steps; m.p. 130~132 °C; ^1^H NMR (500 MHz, DMSO-*d_6_*) δ 12.16 (s, 1H), 7.37 (d, *J* = 8.0 Hz, 2H), 7.33–7.27 (m, 1H), 7.25–7.17 (m, 3H), 7.17–7.11 (m, 2H), 7.08 (dd, *J* = 11.4, 2.3 Hz, 1H), 6.86 (td, *J* = 8.4, 2.4 Hz, 1H), 5.06 (s, 2H), 4.05 (t, *J* = 6.4 Hz, 2H), 2.39 (t, *J* = 7.3 Hz, 2H), 2.32 (s, 3H), 2.03–1.91 (m, 2H); ^13^C NMR (125 MHz, DMSO-*d_6_*) δ 174.44, 163.59, 161.65, 156.65, 152.89, 150.95, 146.45, 136.61, 134.78, 131.87, 130.04, 129.59, 129.05, 127.02, 124.43, 115.86, 115.31, 107.87, 101.81, 69.59, 68.32, 30.41, 24.63, 21.17; HRMS (ES^+^) for C_24_H_22_F_2_O_4_ (M + Na)^+^: calcd 435.1384; found, 435.1385.

*5-(2-fluoro-4-(((4-fluoro-4′-methyl-[1,1′-biphenyl]-2-yl)oxy)methyl)phenoxy)pentanoic acid* (**10l**): Colorless solid 0.24 g, yield 56% of two steps; m.p. 112~114 °C; ^1^H NMR (500 MHz, DMSO-*d_6_*) δ 12.04 (s, 1H), 7.37 (d, *J* = 8.1 Hz, 2H), 7.30 (dd, *J* = 8.4, 7.1 Hz, 1H), 7.24–7.17 (m, 3H), 7.16–7.11 (m, 2H), 7.08 (dd, *J* = 11.4, 2.4 Hz, 1H), 5.06 (s, 2H), 4.03 (t, *J* = 6.3 Hz, 2H), 2.32 (s, 3H), 2.29 (t, *J* = 7.3 Hz, 2H), 1.82–1.70 (m, 2H), 1.70–1.61 (m, 2H); ^13^C NMR (125 MHz, DMSO-*d_6_*) δ 174.80, 163.59, 161.65, 156.65, 152.87, 150.93, 146.58, 136.60, 134.79, 131.87, 129.88, 129.59, 129.05, 127.02, 124.43, 115.84, 115.23, 107.86, 101.81, 69.60, 68.84, 33.70, 28.50, 21.56, 21.17; HRMS (ES^+^) for C_25_H_24_F_2_O_4_ (M + Na)^+^: calcd 449.1540; found, 449.1548.

#### 3.1.6. General Synthetic Procedure for Intermediates **12a**–**12l**

The intermediates **12a**–**12n** were obtained as described for intermediate **8a**.

*4-fluoro-2′-methyl-[1,1′-biphenyl]-2-ol* (**12a**): ^1^H NMR (500 MHz, CDCl_3_) δ 7.33–7.30 (m, 2H), 7.27 (dd, *J* = 7.7, 4.5 Hz, 1H), 7.19 (dt, *J* = 7.1, 1.2 Hz, 1H), 7.04 (dd, *J* = 8.4, 6.5 Hz, 1H), 6.74–6.65 (m, 2H), 2.15 (s, 3H).

*4-fluoro-3′-methyl-[1,1′-biphenyl]-2-ol* (**12b**): ^1^H NMR (500 MHz, CDCl_3_) δ 7.36 (td, *J* = 7.3, 1.0 Hz, 1H), 7.22–7.17 (m, 3H), 7.15 (dd, *J* = 8.3, 6.6 Hz, 1H), 6.72–6.65 (m, 2H), 2.40 (s, 3H).

*2′,4-difluoro-[1,1′-biphenyl]-2-ol* (**12c**): ^1^H NMR (500 MHz, CDCl_3_) δ 7.41–7.36 (m, 1H), 7.34 (td, *J* = 7.5, 1.9 Hz, 1H), 7.27–7.22 (m, 1H), 7.21–7.15 (m, 2H), 6.72 (dtd, *J* = 9.3, 4.0, 2.6 Hz, 2H).

*4,4′-difluoro-[1,1′-biphenyl]-2-ol* (**12d**): ^1^H NMR (500 MHz, CDCl_3_) δ 7.43–7.37 (m, 2H), 7.21–7.13 (m, 3H), 6.73–6.68 (m, 2H).

*4-fluoro-4′-methoxy-[1,1′-biphenyl]-2-ol* (**12e**): ^1^H NMR (500 MHz, CDCl_3_) δ 7.36–7.31 (m, 2H), 7.14 (dd, *J* = 8.3, 6.5 Hz, 1H), 7.05–6.99 (m, 2H), 6.73–6.66 (m, 2H), 3.86 (s, 3H).

*4-fluoro-2′,4′-dimethyl-[1,1′-biphenyl]-2-ol* (**12f**): ^1^H NMR (500 MHz, CDCl_3_) δ 9.80 (s, 1H), 7.04 (d, *J* = 1.8 Hz, 1H), 7.02–6.97 (m, 2H), 6.95 (d, *J* = 7.7 Hz, 1H), 6.69 (dd, *J* = 10.9, 2.6 Hz, 1H), 6.65 (td, *J* = 8.5, 2.6 Hz, 1H), 2.29 (s, 3H), 2.06 (s, 3H).

*4-fluoro-3′,4′-dimethyl-[1,1′-biphenyl]-2-ol* (**12g**): ^1^H NMR (500 MHz, CDCl_3_) δ 7.23 (d, *J* = 7.6 Hz, 1H), 7.16 (d, *J* = 2.0 Hz, 1H), 7.15–7.10 (m, 2H), 6.70–6.64 (m, 2H), 2.30 (s, 6H).

*4-fluoro-3′,5′-dimethyl-[1,1′-biphenyl]-2-ol* (**12h**): ^1^H NMR (500 MHz, CDCl_3_) δ 7.12 (dd, *J* = 8.3, 6.5 Hz, 1H), 7.02–6.99 (m, 1H), 6.99 (d, *J* = 1.4 Hz, 2H), 6.69–6.62 (m, 2H), 2.33 (s, 6H).

*2′,4′-dichloro-4-fluoro-[1,1′-biphenyl]-2-ol* (**12i**): ^1^H NMR (500 MHz, CDCl_3_) δ 7.53 (d, *J* = 2.1 Hz, 1H), 7.33 (dd, *J* = 8.2, 2.1 Hz, 1H), 7.25 (d, *J* = 8.2 Hz, 1H), 7.11–7.06 (m, 1H), 6.75–6.67 (m, 2H).

*4-fluoro-4′-methoxy-2′-methyl-[1,1′-biphenyl]-2-ol* (**12j**): ^1^H NMR (500 MHz, CDCl_3_) δ 9.69 (d, *J* = 3.3 Hz, 1H), 7.67 (dd, *J* = 8.8, 2.7 Hz, 1H), 7.36–7.26 (m, 2H), 7.08 (d, *J* = 8.3 Hz, 1H), 6.85 (d, *J* = 2.7 Hz, 1H), 6.81 (dd, *J* = 8.3, 2.7 Hz, 1H), 3.85 (s, 3H), 2.08 (s, 3H).

*2′-chloro-4-fluoro-4′-methoxy-[1,1′-biphenyl]-2-ol* (**12k**): ^1^H NMR (500 MHz, CDCl_3_) δ 7.22 (d, *J* = 8.5 Hz, 1H), 7.11–7.06 (m, 2H), 6.91 (dd, *J* = 8.5, 2.6 Hz, 1H), 6.74–6.67 (m, 2H), 3.85 (s, 3H).

*2′,4-difluoro-4′-methoxy-[1,1′-biphenyl]-2-ol* (**12l**): ^1^H NMR (500 MHz, CDCl_3_) δ 7.24 (t, *J* = 8.6 Hz, 1H), 7.19–7.12 (m, 1H), 6.81 (dd, *J* = 8.5, 2.6 Hz, 1H), 6.76 (dd, *J* = 11.6, 2.5 Hz, 1H), 6.74–6.68 (m, 2H), 3.85 (s, 3H).

*2′-chloro-4-fluoro-4′-methyl-[1,1′-biphenyl]-2-ol* (**12m**): ^1^H NMR (500 MHz, CDCl_3_) δ 7.35 (s, 1H), 7.20 (d, *J* = 7.7 Hz, 1H), 7.17 (dd, *J* = 7.9, 1.7 Hz, 1H), 7.10 (dd, *J* = 8.3, 6.5 Hz, 1H), 6.74–6.68 (m, 2H), 2.39 (s, 3H).

*2′,4-difluoro-4′-methyl-[1,1′-biphenyl]-2-ol* (**12n**): ^1^H NMR (500 MHz, CDCl_3_) δ 7.20 (t, *J* = 7.8 Hz, 1H), 7.17–7.13 (m, 1H), 7.04 (ddd, *J* = 7.7, 1.7, 0.8 Hz, 1H), 7.02–6.97 (m, 1H), 6.72–6.67 (m, 2H), 2.38 (s, 3H).

#### 3.1.7. General Synthetic Procedure for Intermediates **13a**–**13n**

The intermediates **13a**–**13n** were obtained as described for intermediates **9a**–**9l**, which were used in the next step without further purification.

#### 3.1.8. General Synthetic Procedure for Target Compounds **14a**–**14n**

The target compounds **14a**–**14n** were obtained as described for target compounds **10a**–**10l**.

*4-(2-fluoro-4-(((4-fluoro-2′-methyl-[1,1′-biphenyl]-2-yl)oxy)methyl)phenoxy)butanoic acid* (**14a**): Colorless solid 0.23 g, yield 57% of two steps; m.p. 83~85 °C; ^1^H NMR (500 MHz, DMSO-*d*_6_) δ 7.25 (d, *J* = 4.4 Hz, 2H), 7.22–7.18 (m, 1H), 7.14 (d, *J* = 7.7 Hz, 1H), 7.12–7.06 (m, 3H), 7.01 (t, *J* = 8.3 Hz, 2H), 6.85 (td, *J* = 8.4, 2.5 Hz, 1H), 5.03 (s, 2H), 4.02 (t, *J* = 6.5 Hz, 2H), 2.36 (t, *J* = 7.3 Hz, 2H), 2.05 (s, 3H), 1.96–1.88 (m, 2H); ^13^C NMR (125 MHz, DMSO-*d*_6_) δ 174.07, 163.29, 161.35, 156.26, 152.39, 150.45, 145.92, 137.45, 136.19, 131.60, 130.04, 129.57, 127.41, 126.83, 125.51, 123.74, 115.06, 114.81, 107.06, 101.05, 68.88, 67.85, 29.99, 24.18, 19.69; HRMS (ES^+^) for C_24_H_22_F_2_O_4_ (M + Na)^+^: calcd 435.1384; found, 435.1388.

*4-(2-fluoro-4-(((4-fluoro-3′-methyl-[1,1′-biphenyl]-2-yl)oxy)methyl)phenoxy)butanoic acid* (**14b**): Colorless solid 0.22 g, yield 54% of two steps; m.p. 87~89 °C; ^1^H NMR (500 MHz, DMSO-*d*_6_) δ 7.38–7.30 (m, 2H), 7.29–7.19 (m, 3H), 7.18–7.06 (m, 4H), 6.87 (dt, *J* = 9.2, 4.7 Hz, 1H), 5.06 (s, 2H), 4.05 (t, *J* = 6.5 Hz, 2H), 2.38 (t, *J* = 7.2 Hz, 2H), 2.32 (s, 3H), 2.03–1.90 (m, 2H); ^13^C NMR (125 MHz, DMSO-*d*_6_) δ 174.55, 163.76, 161.82, 156.68, 152.97, 151.03, 146.43, 137.46, 131.92, 130.63, 130.11, 128.45, 128.10, 127.21, 126.83, 124.39, 115.78, 115.33, 107.88, 101.80, 69.62, 68.38, 30.46, 24.66, 21.48; HRMS (ES^+^) for C_24_H_22_F_2_O_4_ (M + Na)^+^: calcd 435.1384; found, 435.1386.

*4-(4-(((2′,4-difluoro-[1,1′-biphenyl]-2-yl)oxy)methyl)-2-fluorophenoxy)butanoic acid* (**14c**): Colorless solid 0.23 g, yield 55% of two steps; m.p. 94~96 °C; ^1^H NMR (500 MHz, DMSO-*d*_6_) δ 7.44–7.38 (m, 1H), 7.36 (td, *J* = 7.6, 1.8 Hz, 1H), 7.33–7.22 (m, 3H), 7.22–7.05 (m, 4H), 6.89 (td, *J* = 8.4, 2.5 Hz, 1H), 5.07 (s, 2H), 4.04 (t, *J* = 6.5 Hz, 2H), 2.38 (t, *J* = 7.3 Hz, 2H), 2.05–1.89 (m, 2H); ^13^C NMR (125 MHz, DMSO-*d*_6_) δ 174.48, 164.28, 162.34, 160.89, 158.93, 157.07, 152.86, 150.92, 146.35, 132.42, 130.06, 125.39, 124.70, 124.11, 121.44, 115.74, 115.42, 115.24, 107.66, 101.55, 69.42, 68.28, 30.40, 24.60; HRMS (ES^+^) for C_23_H_19_F_3_O_4_ (M + Na)^+^: calcd 439.1133; found, 439.1130.

*4-(4-(((4,4′-difluoro-[1,1′-biphenyl]-2-yl)oxy)methyl)-2-fluorophenoxy)butanoic acid* (**14d**): Colorless solid 0.24 g, yield 57% of two steps; m.p. 89~91 °C; ^1^H NMR (500 MHz, DMSO-*d*_6_) δ 7.56–7.48 (m, 1H), 7.33 (dd, *J* = 8.4, 6.9 Hz, 1H), 7.25–7.16 (m, 3H), 7.16–7.09 (m, 3H), 6.88 (td, *J* = 8.4, 2.5 Hz, 1H), 5.07 (s, 2H), 4.05 (t, *J* = 6.4 Hz, 2H), 2.38 (t, *J* = 7.3 Hz, 2H), 2.00–1.90 (m, 2H); ^13^C NMR (125 MHz, DMSO-*d*_6_) δ 174.46, 163.80, 162.75, 161.86, 160.81, 156.56, 152.89, 150.95, 146.41, 133.99, 132.00, 131.70, 129.93, 126.02, 124.39, 115.73, 115.48, 107.89, 101.82, 69.65, 68.32, 30.41, 24.62; HRMS (ES^+^) for C_23_H_19_F_3_O_4_ (M + Na)^+^: calcd 439.1133; found, 439.1140.

*4-(2-fluoro-4-(((4-fluoro-4′-methoxy-[1,1′-biphenyl]-2-yl)oxy)methyl)phenoxy)butanoic acid* (**14e**): Colorless solid 0.22 g, yield 53% of two steps; m.p. 103~105 °C; ^1^H NMR (500 MHz, DMSO-*d*_6_) δ 12.14 (s, 1H), 7.44–7.37 (m, 2H), 7.28 (dd, *J* = 8.4, 6.9 Hz, 1H), 7.22–7.16 (m, 1H), 7.15–7.09 (m, 2H), 7.06 (dd, *J* = 11.4, 2.5 Hz, 1H), 6.97–6.91 (m, 2H), 6.84 (td, *J* = 8.3, 2.5 Hz, 1H), 5.05 (s, 2H), 4.04 (t, *J* = 6.4 Hz, 2H), 3.77 (s, 3H), 2.37 (t, *J* = 7.3 Hz, 2H), 2.03–1.89 (m, 2H); ^13^C NMR (125 MHz, DMSO-*d*_6_) δ 174.46, 163.43, 161.49, 158.80, 156.53, 152.91, 150.97, 146.39, 131.71, 130.85, 130.08, 129.94, 126.80, 124.37, 115.74, 115.36, 113.94, 107.78, 101.74, 69.57, 68.34, 55.56, 30.43, 24.64; HRMS (ES^+^) for C_24_H_22_F_2_O_5_ (M + Na)^+^: calcd 451.1333; found, 451.1334.

*4-(2-fluoro-4-(((4-fluoro-2′,4′-dimethyl-[1,1′-biphenyl]-2-yl)oxy)methyl)phenoxy)butanoic acid* (**14f**): Colorless solid 0.24 g, yield 56% of two steps; m.p. 84~86 °C; ^1^H NMR (500 MHz, DMSO-*d*_6_) δ 7.09 (dd, *J* = 8.6, 6.7 Hz, 2H), 7.07–7.04 (m, 2H), 7.04–6.99 (m, 3H), 6.97 (d, *J* = 7.7 Hz, 1H), 6.83 (td, *J* = 8.4, 2.4 Hz, 1H), 5.01 (s, 2H), 4.03 (t, *J* = 6.4 Hz, 2H), 2.37 (t, *J* = 7.3 Hz, 2H), 2.29 (s, 3H), 2.02 (s, 3H), 1.99–1.88 (m, 2H); ^13^C NMR (125 MHz, DMSO-*d*_6_) δ 174.45, 163.60, 161.67, 156.80, 152.83, 146.38, 136.83, 136.34, 134.96, 132.15, 130.50, 130.04, 127.25, 126.54, 124.31, 115.63, 115.29, 107.42, 101.44, 69.34, 68.31, 30.41, 24.62, 21.14, 20.05; HRMS (ES^+^) for C_25_H_24_F_2_O_4_ (M + Na)^+^: calcd 449.1540; found, 449.1541.

*4-(2-fluoro-4-(((4-fluoro-3′,4′-dimethyl-[1,1′-biphenyl]-2-yl)oxy)methyl)phenoxy)butanoic acid* (**14g**): Colorless solid 0.23 g, yield 53% of two steps; m.p. 116~118 °C; ^1^H NMR (500 MHz, DMSO-*d*_6_) δ 12.19 (s, 1H), 7.38–7.31 (m, 2H), 7.31–7.25 (m, 1H), 7.25–7.15 (m, 4H), 7.13 (dd, *J* = 11.3, 2.6 Hz, 1H), 6.90 (td, *J* = 8.3, 2.5 Hz, 1H), 5.10 (s, 2H), 4.10 (t, *J* = 6.4 Hz, 2H), 2.43 (t, *J* = 7.3 Hz, 2H), 2.28 (s, 6H), 2.04–1.95 (m, 2H). ^13^C NMR (125 MHz, DMSO-*d*_6_) δ 174.45, 163.53, 161.60, 156.61, 152.93, 150.99, 146.38, 136.02, 135.20, 131.71, 130.97, 130.09, 129.59, 126.98, 124.40, 115.80, 115.30, 107.75, 101.69, 69.58, 68.35, 30.40, 24.62, 19.77, 19.50; HRMS (ES^+^) for C_25_H_24_F_2_O_4_ (M + Na)^+^: calcd 449.1540; found, 449.1545.

*4-(2-fluoro-4-(((4-fluoro-3′,5′-dimethyl-[1,1′-biphenyl]-2-yl)oxy)methyl)phenoxy)butanoic acid* (**14h**): Colorless solid 0.26 g, yield 61% of two steps; m.p. 96~98 °C; ^1^H NMR (500 MHz, DMSO-*d*_6_) δ 7.31 (dd, *J* = 8.5, 6.9 Hz, 1H), 7.23 (d, *J* = 13.2 Hz, 1H), 7.14 (d, *J* = 6.6 Hz, 2H), 7.12–7.03 (m, 3H), 6.94 (s, 1H), 6.85 (td, *J* = 8.4, 2.5 Hz, 1H), 5.05 (s, 2H), 4.05 (t, *J* = 6.4 Hz, 2H), 2.38 (t, *J* = 7.3 Hz, 2H), 2.28 (s, 6H), 1.99–1.90 (m, 2H); ^13^C NMR (125 MHz, DMSO-*d*_6_) δ 174.45, 163.64, 161.71, 156.64, 152.97, 151.03, 146.35, 137.46, 137.27, 131.71, 130.15, 128.77, 127.62, 127.27, 124.22, 115.67, 115.30, 107.77, 101.74, 69.55, 68.38, 30.40, 24.61, 21.32; HRMS (ES^+^) for C_25_H_24_F_2_O_4_ (M + Na)^+^: calcd 449.1540; found, 449.1543.

*4-(4-(((2′,4′-dichloro-4-fluoro-[1,1′-biphenyl]-2-yl)oxy)methyl)-2-fluorophenoxy)butanoic acid* (**14i**): Colorless solid 0.29 g, yield 62% of two steps; m.p. 109~111 °C; ^1^H NMR (500 MHz, DMSO-*d*_6_) δ 12.16 (s, 1H), 7.69 (d, *J* = 2.2 Hz, 1H), 7.46 (dd, *J* = 8.3, 2.2 Hz, 1H), 7.36 (d, *J* = 8.2 Hz, 1H), 7.22 (t, *J* = 7.6 Hz, 1H), 7.18–7.00 (m, 4H), 6.89 (td, *J* = 8.5, 2.4 Hz, 1H), 5.05 (s, 2H), 4.04 (t, *J* = 6.5 Hz, 2H), 2.37 (t, *J* = 7.4 Hz, 2H), 2.06–1.83 (m, 2H); ^13^C NMR (125 MHz, DMSO-*d*_6_) δ 174.46, 164.38, 162.43, 156.93, 152.81, 150.87, 146.43, 135.93, 134.53, 133.72, 133.39, 132.23, 129.77, 129.03, 127.63, 124.30, 115.67, 115.24, 107.67, 101.60, 69.51, 68.27, 30.40, 24.60; HRMS (ES^+^) for C_23_H_18_Cl_2_F_2_O_4_ (M + Na)^+^: calcd 489.0448; found, 489.0450.

*4-(2-fluoro-4-(((4-fluoro-4′-methoxy-2′-methyl-[1,1′-biphenyl]-2-yl)oxy)methyl)phenoxy)butanoic acid* (**14j**): Colorless solid 0.26 g, yield 58% of two steps; m.p. 87~89 °C; ^1^H NMR (500 MHz, DMSO-*d*_6_) δ 7.09 (t, *J* = 7.9 Hz, 2H), 7.06–6.99 (m, 4H), 6.86–6.79 (m, 2H), 6.76 (dd, *J* = 8.4, 2.7 Hz, 1H), 5.00 (s, 2H), 4.02 (t, *J* = 6.4 Hz, 2H), 3.75 (s, 3H), 2.36 (t, *J* = 7.3 Hz, 2H), 2.03 (s, 3H), 1.92 (t, *J* = 6.9 Hz, 2H); ^13^C NMR (125 MHz, DMSO-*d*_6_) δ 174.46, 163.56, 161.63, 158.93, 156.92, 152.85, 150.91, 146.36, 138.00, 132.35, 131.46, 130.18, 130.08, 127.04, 124.20, 115.34, 111.34, 107.39, 101.44, 69.32, 68.32, 55.42, 30.42, 24.62, 20.38; HRMS (ES^+^) for C_25_H_24_F_2_O_5_ (M + Na)^+^: calcd 465.1489; found, 465.1492.

*4-(4-(((2′-chloro-4-fluoro-4′-methoxy-[1,1′-biphenyl]-2-yl)oxy)methyl)-2-fluorophenoxy)butanoic acid* (**14k**): Colorless solid 0.25 g, yield 55% of two steps; m.p. 102~104 °C; ^1^H NMR (500 MHz, DMSO-*d*_6_) δ 7.23 (d, *J* = 8.5 Hz, 1H), 7.17 (dd, *J* = 8.4, 6.9 Hz, 1H), 7.15–7.10 (m, 2H), 7.10–7.02 (m, 3H), 6.95 (dd, *J* = 8.5, 2.5 Hz, 1H), 6.85 (td, *J* = 8.4, 2.5 Hz, 1H), 5.05 (s, 2H), 4.04 (t, *J* = 6.4 Hz, 2H), 3.79 (s, 3H), 2.38 (t, *J* = 7.3 Hz, 2H), 2.05–1.85 (m, 2H); ^13^C NMR (125 MHz, DMSO-*d*_6_) δ 174.47, 162.10, 159.77, 157.19, 152.89, 150.95, 146.38, 134.01, 133.01, 132.53, 128.99, 124.20, 115.54, 115.32, 114.80, 113.51, 107.39, 101.51, 69.42, 68.35, 56.07, 30.43, 24.65; HRMS (ES^+^) for C_24_H_21_ClF_2_O_5_ (M + Na)^+^: calcd 485.0943; found, 485.0948.

*4-(4-(((2′,4-difluoro-4′-methoxy-[1,1′-biphenyl]-2-yl)oxy)methyl)-2-fluorophenoxy)butanoic acid* (**14l**): Colorless solid 0.26 g, yield 59% of two steps; m.p. 116~118 °C; ^1^H NMR (500 MHz, DMSO-*d*_6_) δ 7.27 (d, *J* = 8.6 Hz, 1H), 7.25–7.22 (m, 1H), 7.15–7.10 (m, 2H), 7.10–7.03 (m, 2H), 6.90–6.84 (m, 2H), 6.82 (dd, *J* = 8.5, 2.6 Hz, 1H), 5.05 (s, 2H), 4.04 (t, *J* = 6.4 Hz, 2H), 3.79 (s, 3H), 2.37 (t, *J* = 7.3 Hz, 2H), 1.99–1.88 (m, 2H); ^13^C NMR (125 MHz, DMSO-*d*_6_) δ 174.53, 162.10, 160.62, 159.48, 157.15, 152.91, 150.98, 146.38, 132.66, 130.05, 124.14, 121.39, 117.37, 115.53, 115.35, 110.56, 107.59, 101.90, 101.54, 69.42, 68.36, 56.12, 30.48, 24.67; HRMS (ES^+^) for C_24_H_21_F_3_O_5_ (M + Na)^+^: calcd 469.1239; found, 469.1242.

*4-(4-(((2′-chloro-4-fluoro-4′-methyl-[1,1′-biphenyl]-2-yl)oxy)methyl)-2-fluorophenoxy)butanoic acid* (**14m**): Colorless solid 0.27 g, yield 60% of two steps; m.p. 105~107 °C; ^1^H NMR (500 MHz, DMSO-*d*_6_) δ 7.34 (s, 1H), 7.21–7.14 (m, 3H), 7.14–7.02 (m, 4H), 6.85 (td, *J* = 8.4, 2.5 Hz, 1H), 5.03 (s, 2H), 4.03 (t, *J* = 6.3 Hz, 2H), 2.37 (t, *J* = 7.4 Hz, 2H), 2.33 (s, 3H), 1.97–1.84 (m, 2H); ^13^C NMR (125 MHz, DMSO-*d*_6_) δ 174.50, 164.15, 162.21, 157.10, 152.90, 150.96, 146.45, 139.52, 133.96, 133.17, 132.36, 129.94, 128.15, 124.99, 115.68, 107.44, 101.51, 69.47, 68.33, 30.42, 24.67, 20.85; HRMS (ES^+^) for C_24_H_21_ClF_2_O_4_ (M + Na)^+^: calcd 469.0994; found, 469.0994.

*4-(4-(((2′,4-difluoro-4′-methyl-[1,1′-biphenyl]-2-yl)oxy)methyl)-2-fluorophenoxy)butanoic acid* (**14n**): Colorless solid 0.25 g, yield 57% of two steps; m.p. 92~94 °C; ^1^H NMR (500 MHz, DMSO-*d*_6_) δ 7.09 (dd, *J* = 8.6, 6.7 Hz, 2H), 7.07–7.04 (m, 2H), 7.04–6.99 (m, 3H), 6.97 (d, *J* = 7.7 Hz, 1H), 6.83 (td, *J* = 8.4, 2.4 Hz, 1H), 5.01 (s, 2H), 4.03 (t, *J* = 6.4 Hz, 2H), 2.37 (t, *J* = 7.3 Hz, 2H), 2.29 (s, 3H), 2.02 (s, 3H), 1.99–1.88 (m, 2H); ^13^C NMR (125 MHz, DMSO-*d*_6_) δ 174.47, 164.14, 162.20, 160.69, 158.74, 157.10, 152.86, 150.92, 146.38, 140.11, 132.52, 129.95, 125.28, 124.22, 122.32, 121.46, 116.13, 115.60, 115.23, 107.58, 101.49, 69.42, 68.30, 30.40, 24.61, 21.03; HRMS (ES^+^) for C_24_H_21_F_3_O_4_ (M + Na)^+^: calcd 453.1290; found, 453.1295.

### 3.2. Pharmacology Studies

#### 3.2.1. In Vitro GPR120 Agonistic Activity and Selectivity Studies

A calcium flux assay was performed to assess the agonist activity of the test compounds against the hGPR120 (mGPR120) and hGPR40 (mGPR40) receptors. CHO cells stably transfected with hGPR120 were seeded in a 96-well plate at a density of 20,000 cells per well and incubated for 24 h at 37 °C in a 5% CO_2_ incubator. The medium in the wells was gently discarded, and the wells were washed with Hank’s balanced salt solution (HBSS; 100 μL per well). Cells were then incubated in HBSS containing Fluo-4 AM (2.5 μg/mL), 0.1% fatty acid-free bovine serum albumin, and probenecid (2.5 mM) for 60 min at 37 °C. The cells were then washed thrice in HBSS and allowed to equilibrate for 10 min before conducting the assay. Test compounds dissolved in dimethyl sulfoxide were diluted in HBSS at various concentrations and added to Fluo-4 AM-containing cells, and intracellular Ca^2+^ concentrations were measured using FlexStation3 Molecular Devices. The EC_50_ value of each compound was calculated using GraphPad Prism software (version 5.0; GraphPad Software, San Diego, CA, USA). The calcium influx assay of the target compounds in hGPR40-expressing CHO cells was similar to that of hGPR120.

#### 3.2.2. Animals

Male C57BL/6 mice, eight-week aged, were purchased from Jinan Pengyue Experimental Animal Breeding Co., Ltd. (Jinan, China). The mice were housed in cages under a 12 h light/dark cycle from 7:00 to 19:00 at controlled temperatures (25–26 °C) and relative humidity (50 ± 10%) throughout the experimental period. All animals were allowed to eat and drink freely unless otherwise stated, and were allowed to acclimatize for 1 week before the experiment. All animal experimental protocols were performed following applicable institutional and governmental regulations concerning the ethical use of animals.

##### Pharmacokinetic Analysis of Compounds **10k** and **14d** in C57BL/6 Mice

Pharmacokinetic studies of compounds **10k** and **14d** were performed in male C57BL/6 mice, and TUG-891 was used as a positive control. Male C57BL/6 mice weighing 28–32 g were starved for 12 h and randomly divided into three groups (four mice per group). Compounds **10k**, **14d**, and TUG-891 were dissolved in 0.5% methylcellulose (0.5% MC) at a concentration of 1 mg/mL, and gavage was administered at a volume of 10 mL/kg. Blood samples were collected over a 24 h period post-dose into tubes containing EDTA-K_2_, and plasma was separated with centrifugation at 5.645 g for 10 min. Plasma was collected and precipitated using two volumes of acetonitrile containing an internal standard. This was followed by centrifugation at 15.680 g for 10 min after vortexing for 5 min. The supernatant was diluted with acetonitrile and 10 µL of the supernatant was analyzed by Waters LC-PDA-MS/MS to determine plasma drug levels. Pharmacokinetic parameters were determined using mean data from four mice at each time point. Statistical analysis of the data was performed using the DAS 2.1.1 statistical software program (BioVoice, Shanghai, China).

##### OGTT in Normal C57BL/6 Mice

Normal male C57BL/6 mice aged 9 weeks were used for the OGTT for compound **14d**. C57BL/6 mice were weighed and randomly divided into six groups (eight mice per group) after 12 h of fasting. The test compounds were dissolved in 0.5% MC and vortexed before the study initiation. Mice in each group were gavaged with vehicle (0.5% MC aqueous solution, 10 mL/kg), TUG-891 (30 mg/kg, 10 mL/kg), or compound **14d** (3, 10, 30, 100 mg/kg; 10 mL/kg), 30 min before oral glucose loading (3 g/kg, 10 mL/kg). The exact dose volume was calculated separately for each animal. Blood samples were collected via the tail tip 30 min before the compound dose, at t = 0 (immediately before glucose loading), and 15, 30, 60, and 120 min after glucose loading. Blood glucose levels were measured using blood glucose test strips (Sannuo GA-3 type; Changsha, China). Glucose values were entered into an Excel sheet and plotted using GraphPad Prism software.

##### Anti-Hyperglycemic Effects in DIO Mice

After 1 week of adaptation, male C57BL/6 mice were fed a high-fat diet (45% calories from fat, from Mediscience Ltd., Yangzhou, China) ad libitum for an additional 12 weeks to induce insulin resistance and were used as DIO mice. DIO mice were fasted overnight (12 h), weighed, and randomly divided into three groups (six mice per group). Thereafter, the DIO mice were orally administered a single dose of vehicle (0.5% MC, 10 mL/kg), TUG-891(20 mg/kg, 10 mL/kg), or **14d** (20 mg/kg, 10 mL/kg), 30 min before oral glucose loading (2 g/kg). Blood samples were collected with retro-orbital sinus puncture, and glucose levels were measured in accordance with the OGTT in normal mice. Plasma insulin levels were measured using a mouse insulin RIA kit (Beijing North Institute of Biological Technology, Beijing, China).

### 3.3. Molecular Modeling Study

Homologous modeling was performed to model the structure of the GPR120 receptor using Accelrys Discovery Studio 2020 (DS2020), as the structure of GPR120 has not yet been obtained experimentally. The sequence of human GPR120 was obtained from the UniProtKB database (identifier: Q5NUL3), and NCBI BLAST was used to screen the template proteins. Neurotensin receptor21 (PDB code 4XES), β_2_-adrenoceptor (PDB code 3P0G), and turkey β_1_ adrenoceptor20 (PDB code 6IBL) were selected as templates because of their high homology with the GPR120 receptor. The DS2020 modeler was used according to the manufacturer’s instructions to construct a homology model of the GPR120 receptor after sequence alignment. The model with the lowest DOPE score was evaluated for energy minimization and reliability. The reliable model was used for molecular docking analysis. The CDOCKER molecular docking module in DS2020 was used for molecular docking research of compound **14d**. The docking results were analyzed using the Discovery Studio software.

## 4. Conclusions

In summary, a series of aryloxyalkyl acid derivatives were designed, synthesized and evaluated for their biological activity. The chemical structures of these compounds were determined with ^1^H-NMR, ^13^C-NMR spectroscopy, and HRMS. Among these compounds, compound **14d** was to found have excellent GPR120 agonistic activity and selectivity, and demonstrated excellent pharmacokinetic properties, with high oral exposure and acceptable half-life. The results of the in vivo hypoglycemic evaluation showed that compound **14d** reduced the blood glucose levels in normal mice in a dose-dependent manner, and no hypoglycemic side effects were detected even at a dose of 100 mg/kg. Additionally, compound **14d** significantly increased insulin secretion and played an anti-diabetic role in DIO mice. Molecular simulations showed that compound **14d** bound well to the active site of GPR120 and formed hydrogen bonding interactions with ARG99. Collectively, these results revealed that compound **14d** may be a promising lead compound deserving of further biological evaluation and structural modifications.

## Figures and Tables

**Table 1:** In vitro activities and selectivity of target compounds **10a**–**10l**.

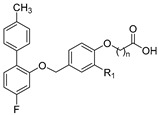					
**Compound**	**n**	**R_1_**	**hGPR120 ^a^** **(EC_50_, nM)**	**hGPR40 ^b^** **(EC_50_, µM)**	**Selectivity**
TUG-891			68.1	72.3	1061.7
**10a**	1	H	92.4	76.2	824.7
**10b**	2	H	218.3	NT	NT
**10c**	3	H	128.6	112.6	875.6
**10d**	4	H	103.2	143.6	1391.5
**10e**	1	CH_3_	138.5	141.2	1019.5
**10f**	2	CH_3_	267.8	NT	NT
**10g**	3	CH_3_	380.4	NT	NT
**10h**	4	CH_3_	205.6	NT	NT
**10i**	1	F	77.2	87.2	1129.5
**10j**	2	F	112.2	85.5	762.0
**10k**	3	F	57.6	74.3	1289.9
**10l**	4	F	96.8	94.7	978.3

NT: Not tested. ^a^ EC_50_ values for hGPR120 activity represent the mean of three determinations. ^b^ EC_50_ values for hGPR40 activity represent the mean of three determinations.

**Table 2:** In vitro activities and selectivity of target compounds **14a**–**14n**.

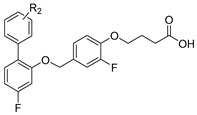				
**Compound**	**R_2_**	**hGPR120 ^a^** **(EC_50_, nM)**	**hGPR40 ^b^** **(EC_50_, µM)**	**Selectivity**
TUG-891	-	68.1	72.3	1061.7
**14a**	2-Me	90.4	68.5	757.7
**14b**	3-Me	82.5	93.2	1129.7
**14c**	2-F	37.5	64.8	1728.0
**14d**	4-F	168.5	84.6	502.1
**14e**	4-OMe	260.2	NT	NT
**14f**	2-Me, 4-OMe	170.2	102.8	604.0
**14g**	2-Cl, 4-OMe	269.5	NT	NT
**14h**	2-F, 4-OMe	421.5	NT	NT
**14i**	2,4-diMe	158.6	95.1	600.0
**14j**	3,4-diMe	150.3	82.3	547.6
**14k**	3,5-diMe	125.6	72.4	576.4
**14l**	2,4-diCl	136.5	90.8	665.2
**14m**	2-Cl, 4-Me	187.3	78.6	419.6
**14n**	2-F, 4-Me	312.7	NT	NT

NT: Not tested. ^a^ EC_50_ values for hGPR120 activity represent the mean of three determinations. ^b^ EC_50_ values for hGPR40 activity represent the mean of three determinations.

**Table 3:** In vitro activities and selectivity of compound **14d** for mGPR120 and mGPR40.

Compound	mGPR120 ^a^ (EC_50_, nM)	mGPR40 ^b^ (EC_50_, µM)	Selectivity
TUG-891	156.1	5.9	37.8
**14d**	83.2	12.7	152.6

^a^ EC_50_ values for mGPR120 activity represent the mean of three determinations. ^b^ EC_50_ values for mGPR40 activity represent the mean of three determinations.

**Table 4:** Pharmacokinetic profiles of compounds **10k** and **14d** in C57BL/6 mice ^a^.

Compound	Dose (mg/kg)	*C*_max_ (ng/mL)	*T*_max_ (min)	AUC (ng.h/mL)	*t*_1/2_ (h)
TUG-891	10.0	1892.3 ± 254.8	15.0	1410.7 ± 157.5	0.6 ± 0.2
**10k**	10.0	2368.5 ± 335.4 *	30.0	4251.6 ± 577.3 ***	1.1 ± 0.3 *
**14d**	10.0	2647.3 ± 287.5 **	30.0	5895.8 ± 583.6 ***^,#^	1.6 ± 0.4 **

^a^ Values are expressed as the mean *±* SD of four C57BL/6 mice. TUG-891 and compounds **10k** and **14d** were suspended in 0.5% MC aqueous solution. * *p ≤* 0.05 compared with TUG-891-treated by Student’s *t* test; ** *p ≤* 0.01 compared with TUG-891-treated by Student’s *t* test; *** *p ≤* 0.001 compared with TUG-891-treated by Student’s *t* test; ^#^ *p ≤* 0.05 compared with **10k**-treated by Student’s *t* test.

## Data Availability

The data presented in this study are available on request from the corresponding author.

## Supplementary Materials

The following supporting information can be downloaded at: https://www.mdpi.com/article/10.3390/molecules27249018/s1, Figure S1: The pharmacokinetic profile traces of compound **14d** and TUG-891; Figure S2: The dose-dependent curves of compound **14d** and TUG-891 for hGPR120 and mGPR120; Tabel S1: EC_50_, Hill Slope, and Hill Slope (95%) of TUG-891 and **14d**; Figures S3–S80: Copies of NMR and HRMS spectra of compounds **10a**–**10l** and **14a**–**14n**.

## Sample Availability

Samples of compounds **10a**–**10l** and **14a**–**14n** are available from the authors.
