# Supplementary material for: Discovery of Novel and Selective G-Protein Coupled Receptor 120 (GPR120) Agonists for the Treatment of Type 2 Diabetes Mellitus
Source: Molecules. 2022 Dec 17;27(24):9018. doi: 10.3390/molecules27249018 (PMC9781217; doi:10.3390/molecules27249018)
Supplement: Supplementary file 1 [file molecules-27-09018-s001.zip › molecules-2027788-supplementary.pdf]

Discovery of Novel and Selective G-Protein Coupled Receptor 120 (GPR120) Agonists for the Treatment of Type 2 Diabetes Mellitus

Xuekun Wang *, Xu Li, Shiting Wei, Min Wang, Yao Xu, Weidi Hu, Zhenzhen Gao, Renmin Liu, Shiben Wang * and Guoxia Ji *

School of Pharmaceutical Sciences, Liaocheng University, 1 Hunan Street, Liaocheng 252059, China; lx221024@126.com (X.L.); wangshiben@lcu.edu.cn (S.W.); 19157166806@163.com (M.W.); xy15665715885@163.com (Y.X.); 15269736603@163.com (W.H.); gaozhenzhen@lcu.edu.cn (Z.G.); liurenmin@lcu.edu.cn (R.L.)

* Correspondence: wangxuekun@lcu.edu.cn (X.W.); xuekunwang0610@126.com (S.W.); jiguoxia@lcu.edu.cn (G.J.); Tel.: +86-0635-823-9087 (X.W.)

The pharmacokinetic profile traces of compound **14d** and TUG-891 were shown in Figure S1.

The dose-dependent curves of compound 14d and TUG-891 for hGPR120 and mGPR120 were shown in Figure S2.

EC_{50}, Hill Slope, and Hill Slope (95%) of TUG-891 and **14d** were shown in Table S1.

The NMR spectra (500 MHz for ^{1}H NMR and 125 MHz for ^{13}C NMR spectra) were recorded using a Bruker AVANCE NEO 500 instrument (compounds were dissolved in DMSO$_{d6}$). Chemical shifts are shown as values relative to the internal standard (tetramethylsilane), and coupling constants (J values) are given in hertz (Hz). High resolution mass spectrometry was conducted using a UPLC G2-XS Qtof spectrometer (Waters) with the electrospray ionization Fourier transform ion cyclotron resonance (ESI-FTICR) technique. The NMR and HRMS spectra of compounds **10a–10l** and **14a-14n** are presented in Figures S3–S80.

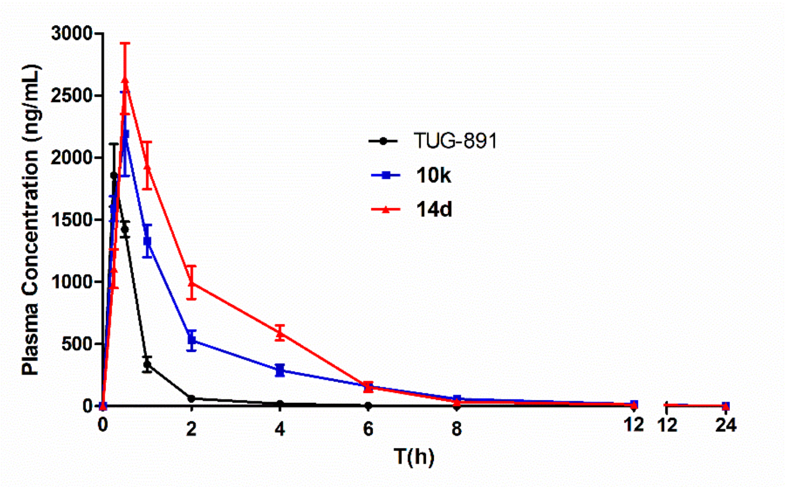

Figure S1. The pharmacokinetic profile traces of compound **14d** and TUG-891.

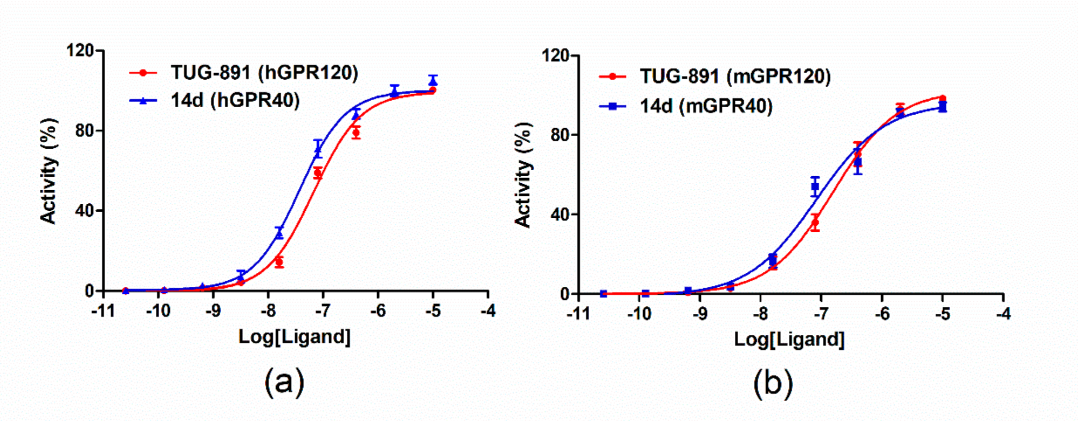

Figure S2. (a) The dose-dependent curves of compound **14d** and TUG-891 for hGPR120; (b) The dose-dependent curves of compound **14d** and TUG-891 for mGPR120.

Table S1. EC_{50}, Hill Slope, and Hill Slope (95%) of TUG-891 and **14d**.

Compound	Receptor	EC_{50} (nM)	Hill slope	Hill slope (95%CI)
TUG-891	hGPR120	68.1	1.0050	0.8356-1.1750
**14d**	hGPR120	37.5	0.9540	0.8259-1.0820
TUG-891	mGPR120	156.1	0.8310	06582-1.0040
**14d**	mGPR120	83.2	0.7745	0.5397-1.0090

EC_{50} value, Hill slope (nH) and 95% CI (95% confidence interval) are from the concentration response curve.

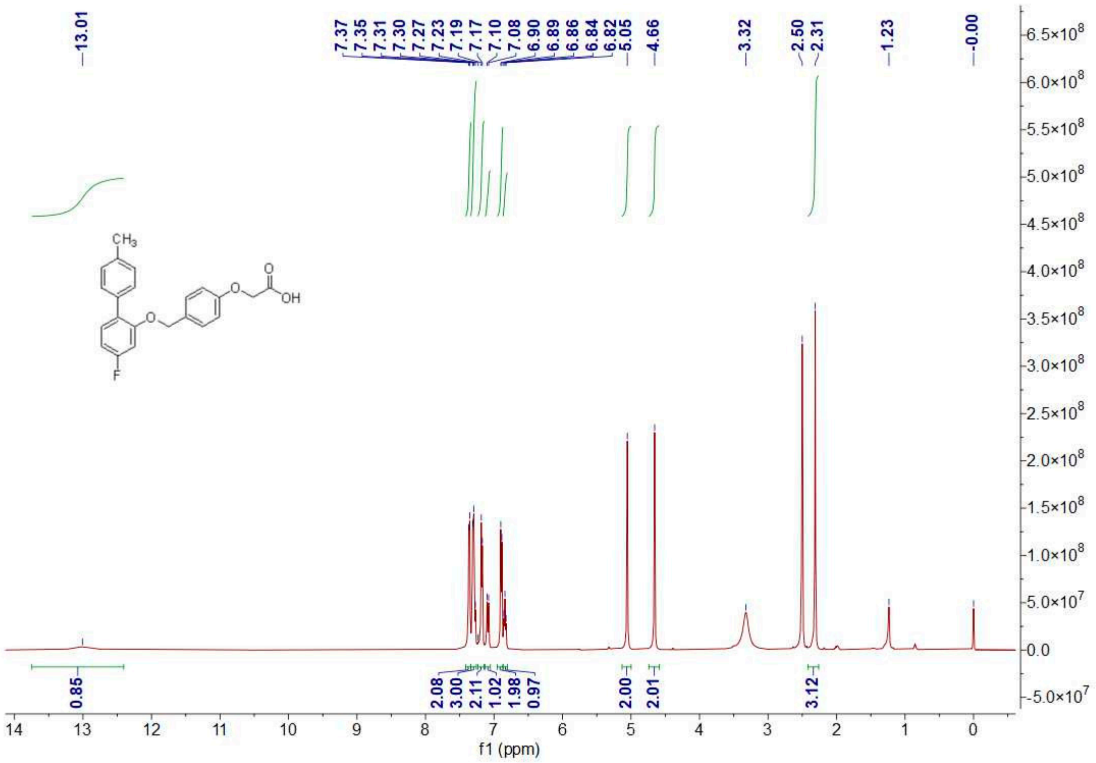

Figure S3. ^{1}H NMR spectrum **10a.**

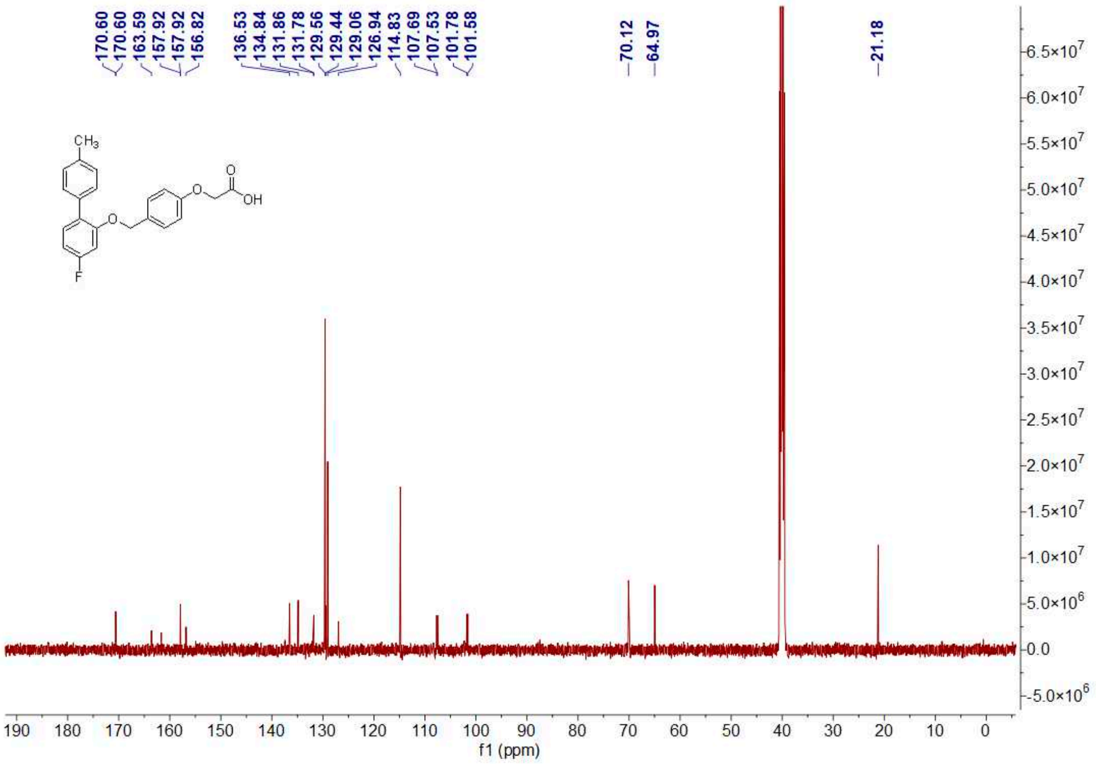

Figure S4. ^{13}C NMR spectrum **10a**.

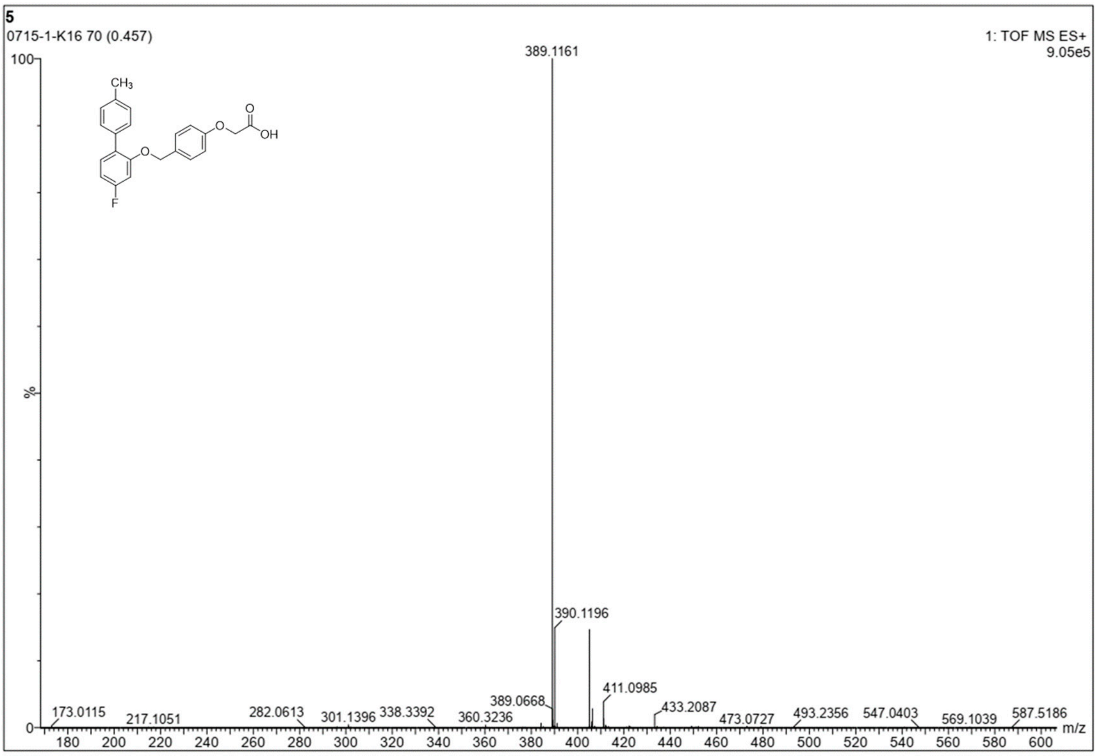

Figure S5. HRMS spectrum **10a.**

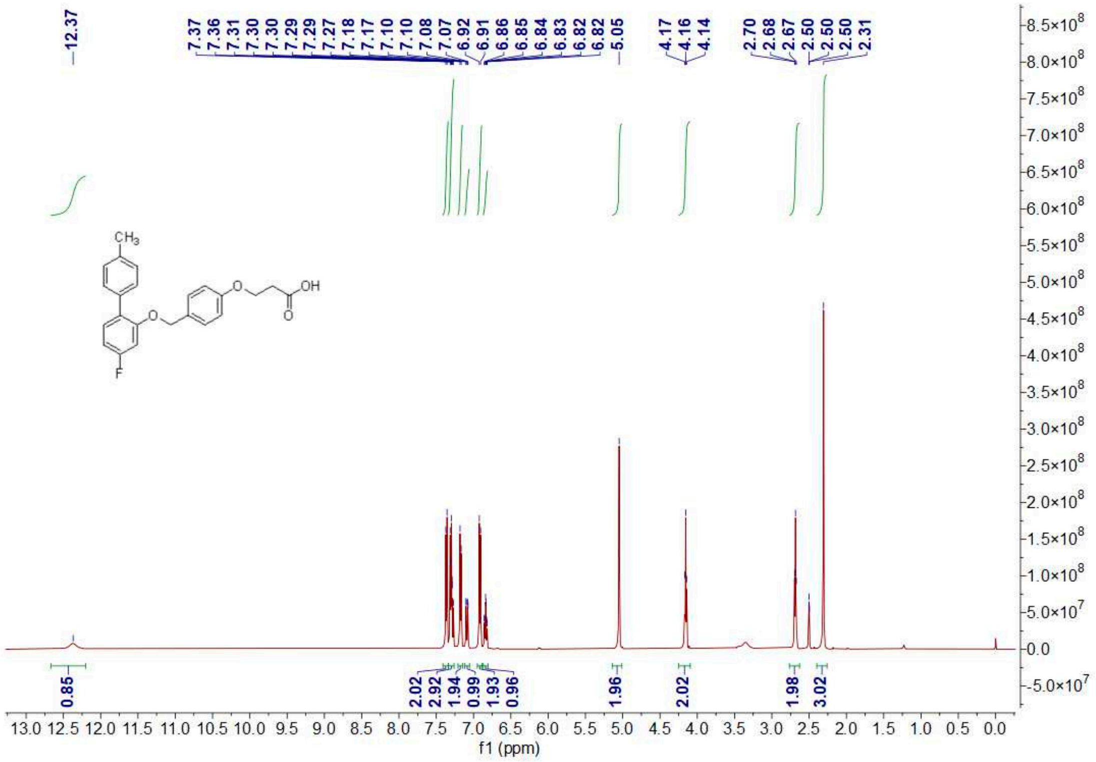

Figure S6. ^{1}H NMR spectrum **10b.**

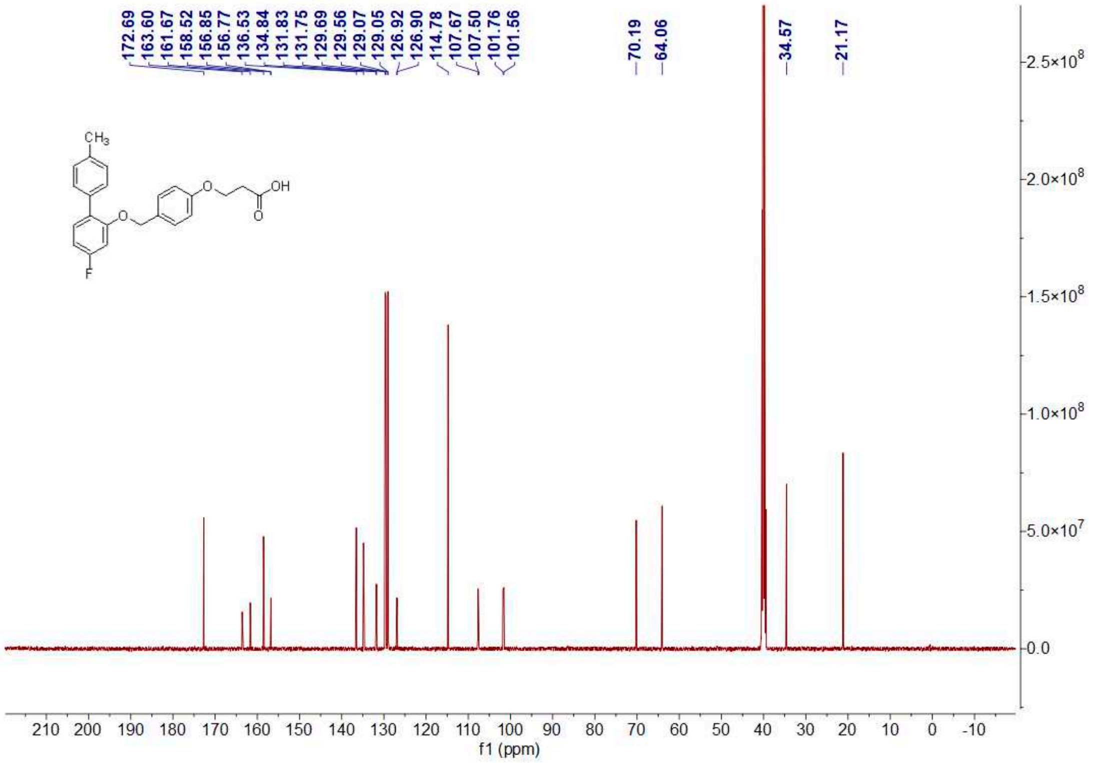

Figure S7. ^{13}C NMR spectrum **10b**.

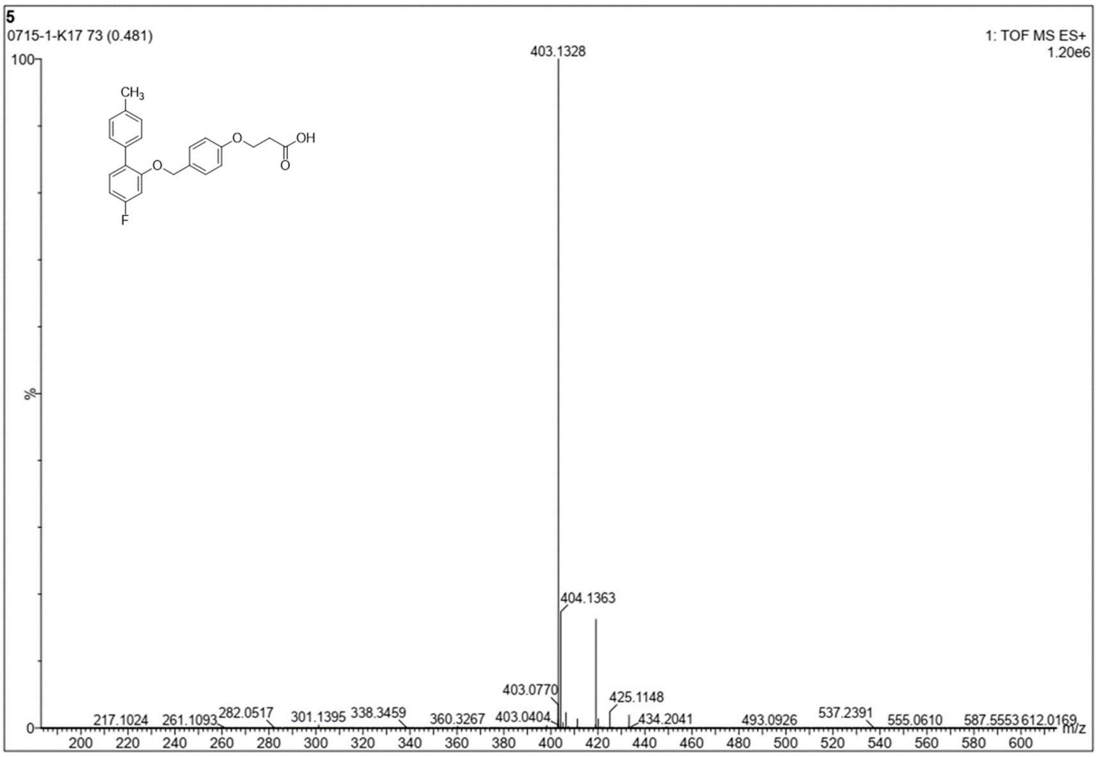

Figure S8. HRMS spectrum **10b.**

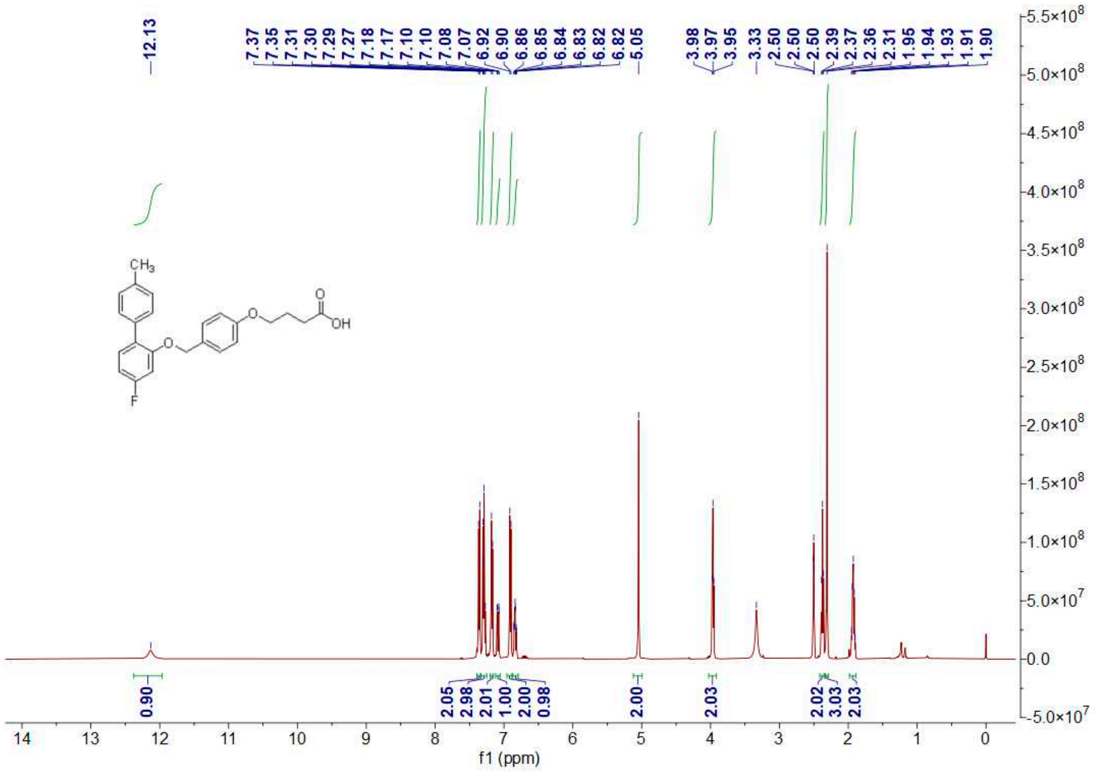

Figure S9. ^{1}H NMR spectrum **10c.**

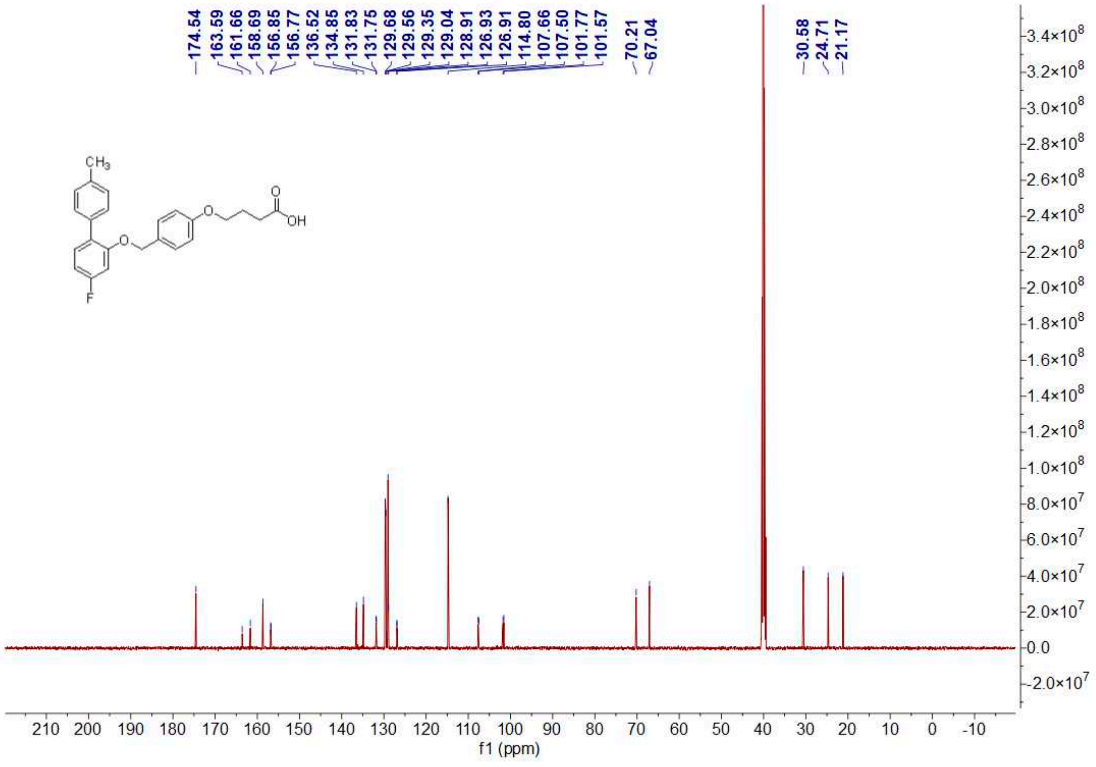

Figure S10. ^{13}C NMR spectrum **10c**.

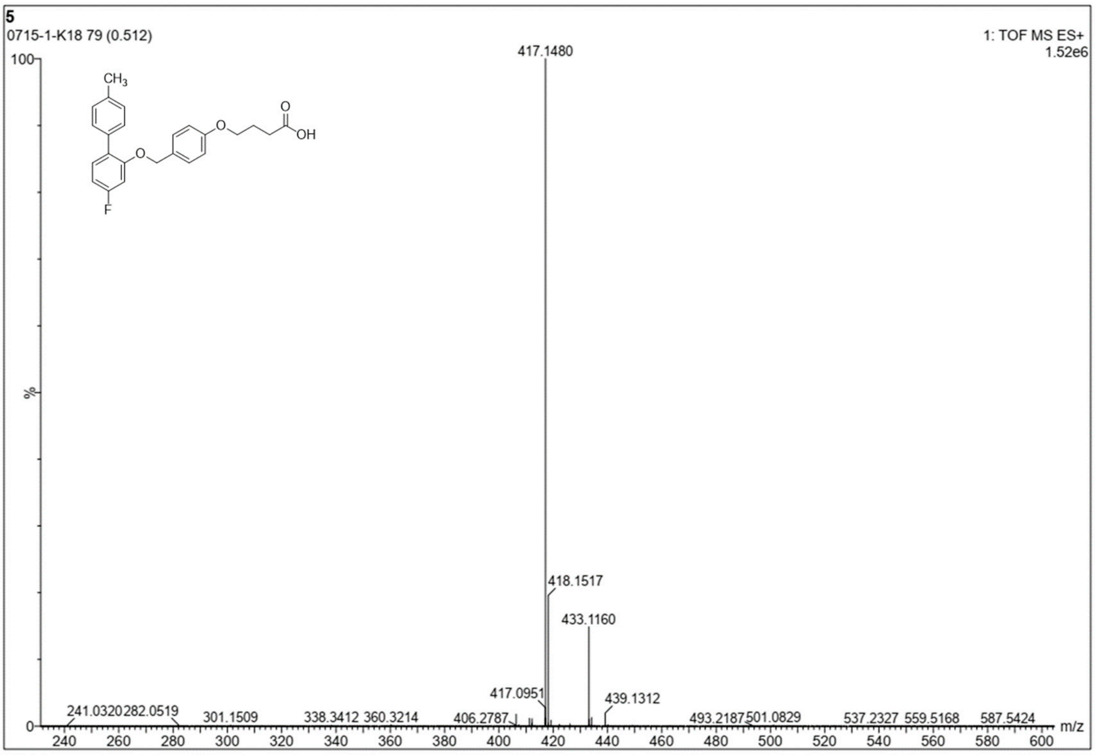

Figure S11. HRMS spectrum **10c.**

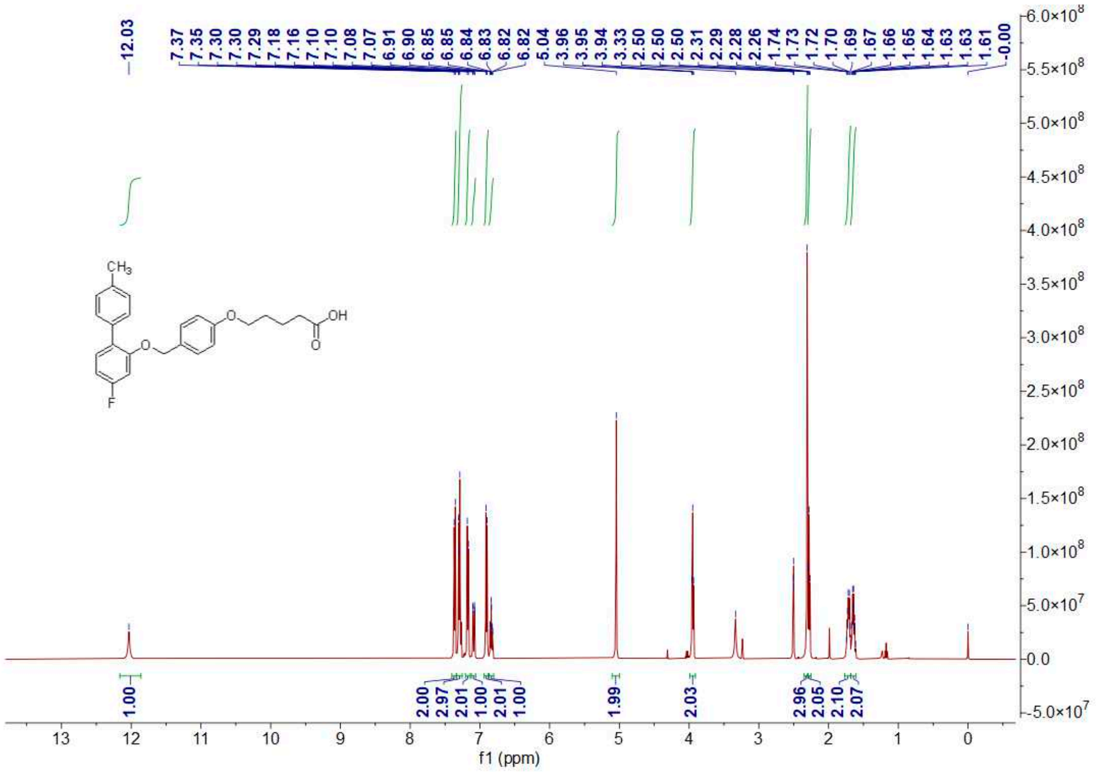

Figure S12. ^{1}H NMR spectrum **10d.**

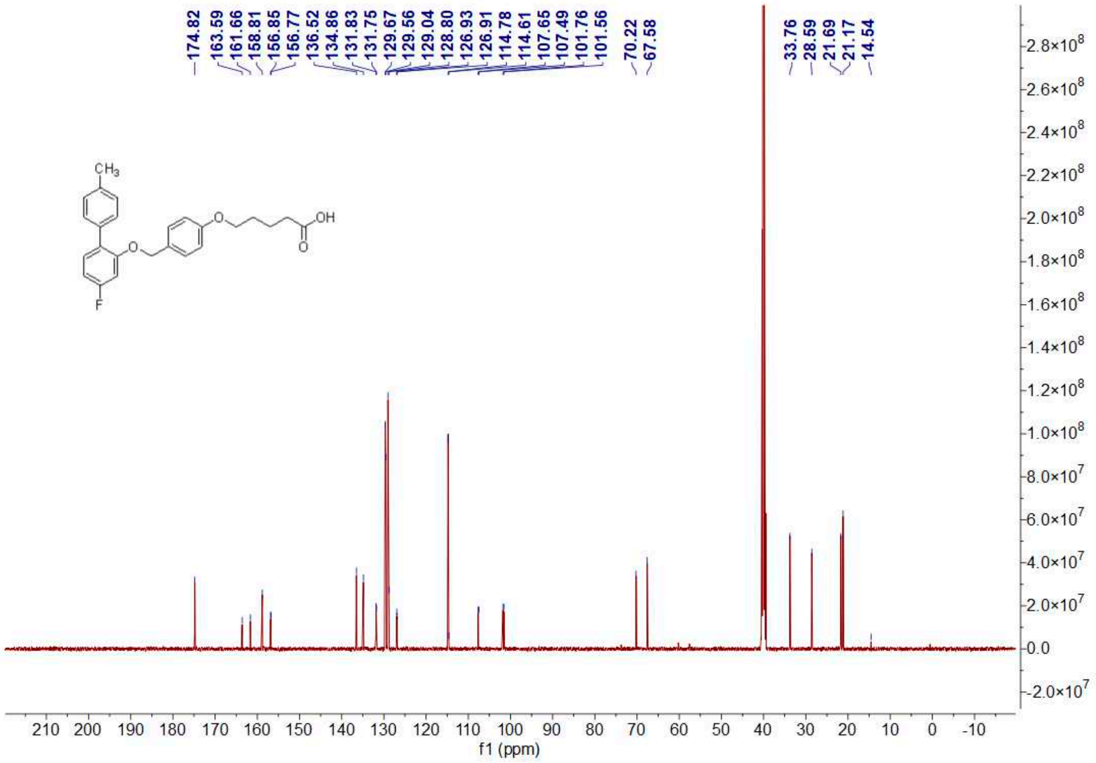

Figure S13. ^{13}C NMR spectrum **10d**.

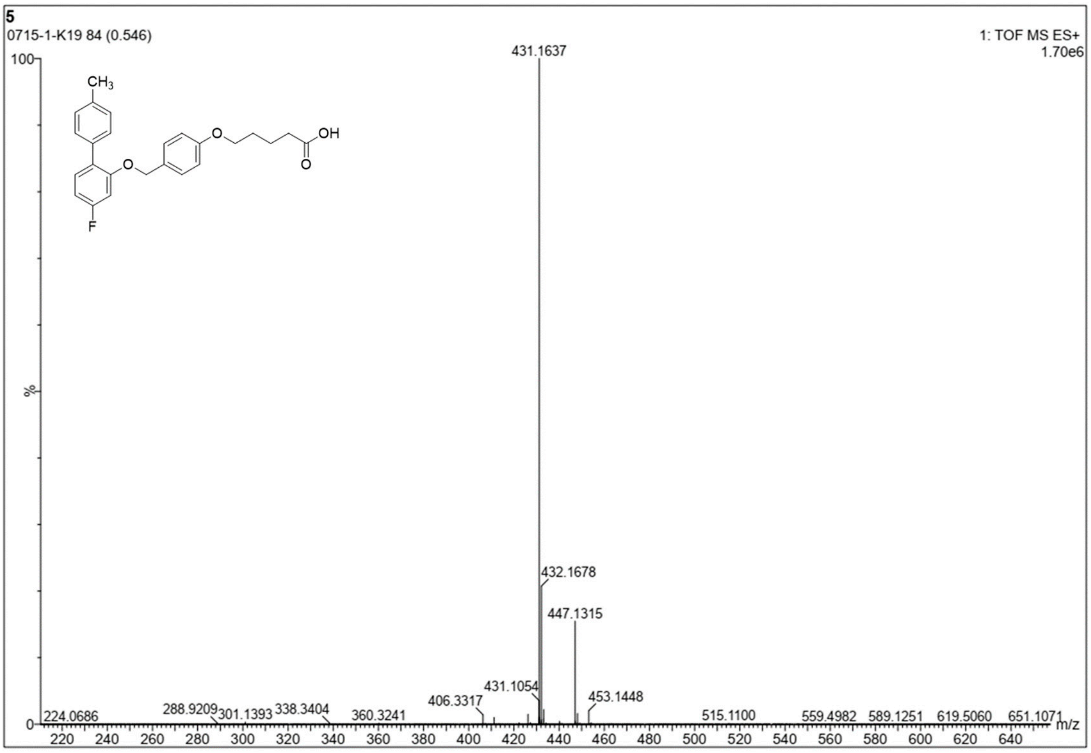

Figure S14. HRMS spectrum **10d.**

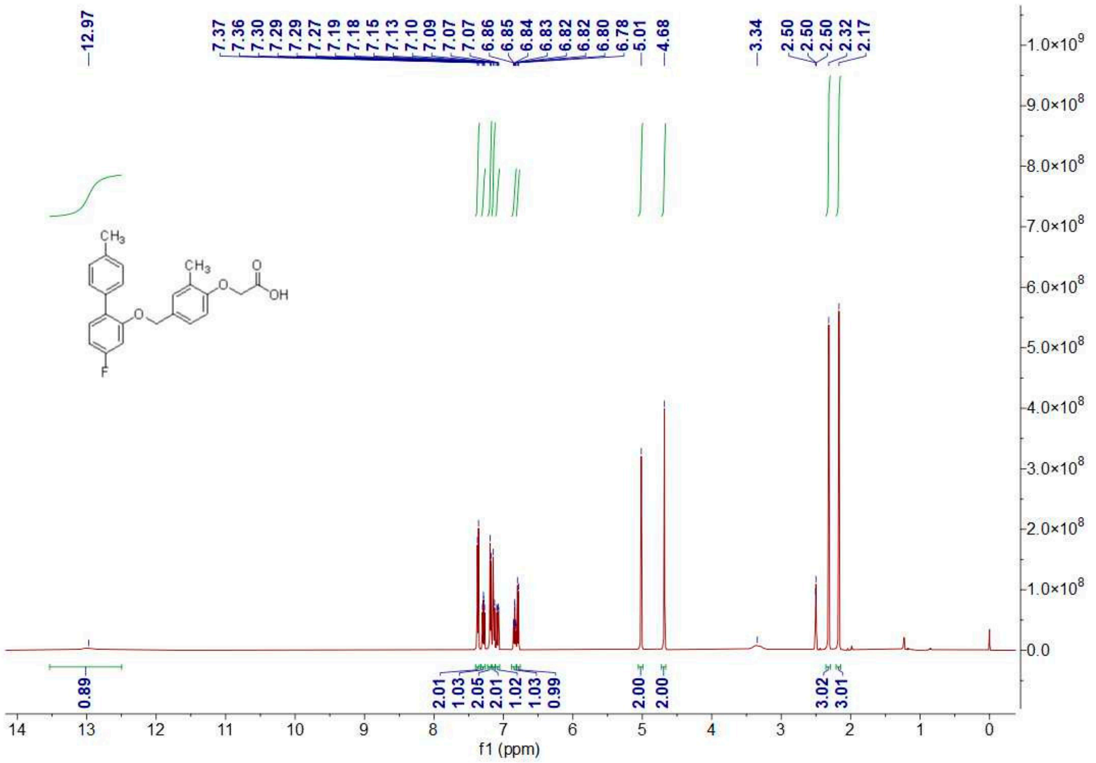

Figure S15. ^{1}H NMR spectrum **10e.**

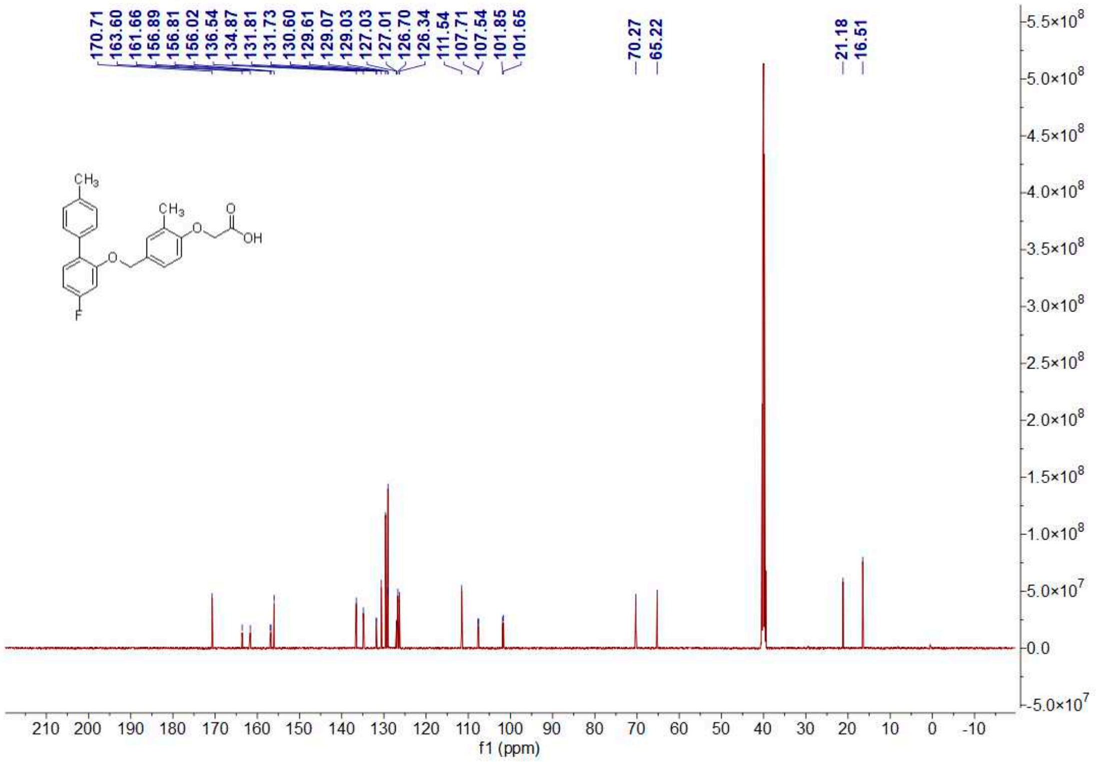

Figure S16. ^{13}C NMR spectrum **10e**.

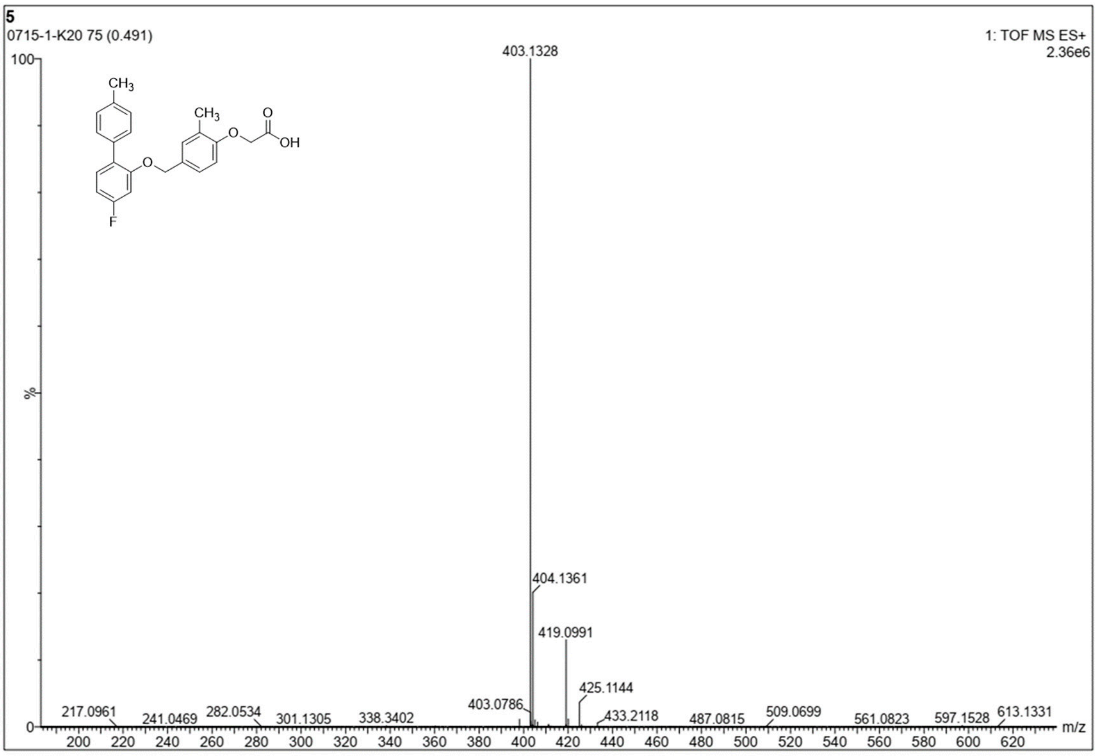

Figure S17. HRMS spectrum **10e.**

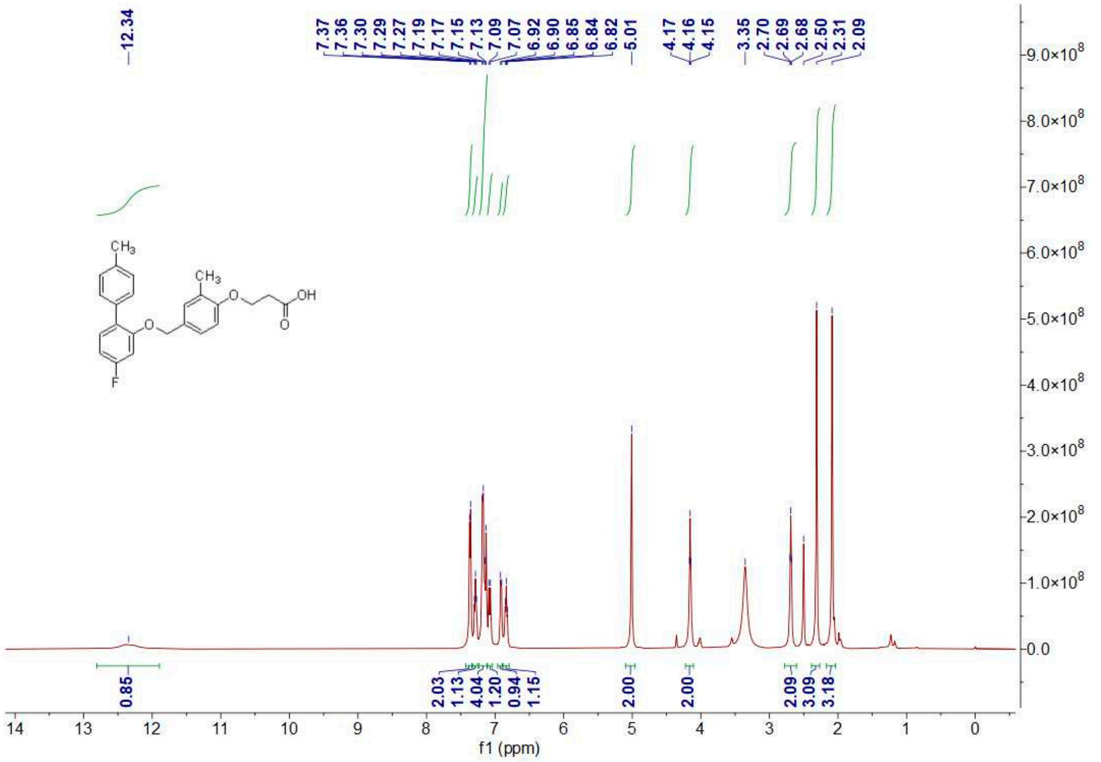

Figure S18. ^{1}H NMR spectrum **10f.**

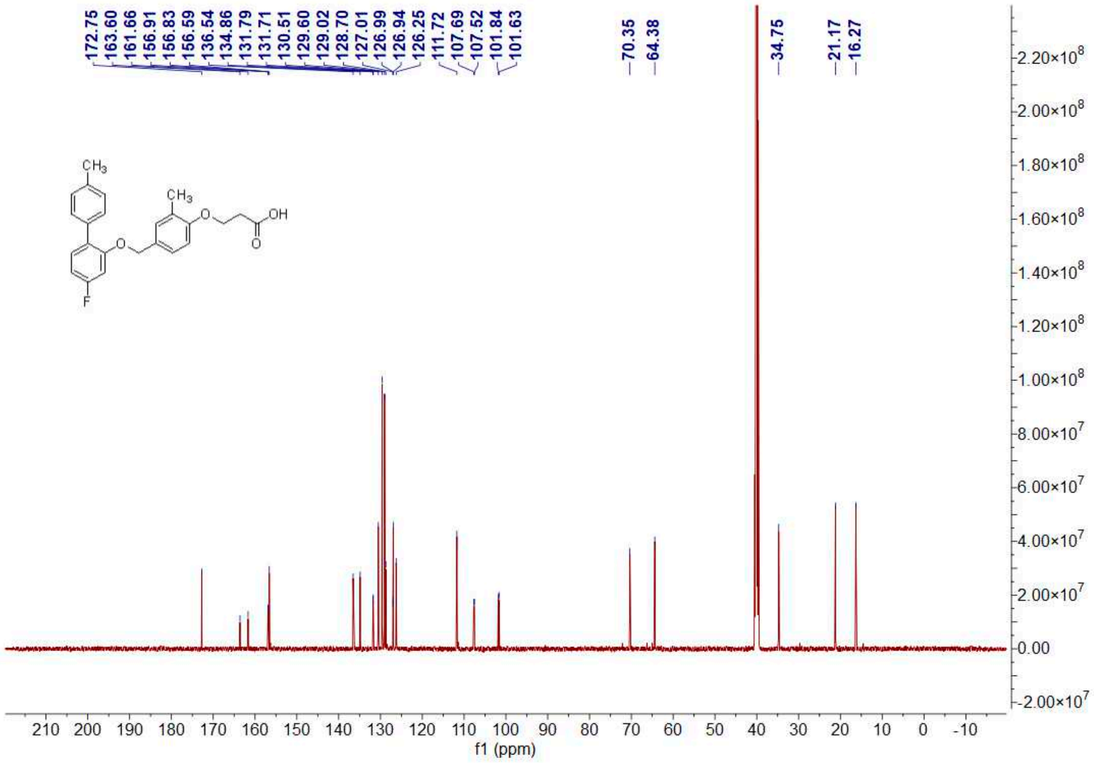

Figure S19. ^{13}C NMR spectrum **10f**.

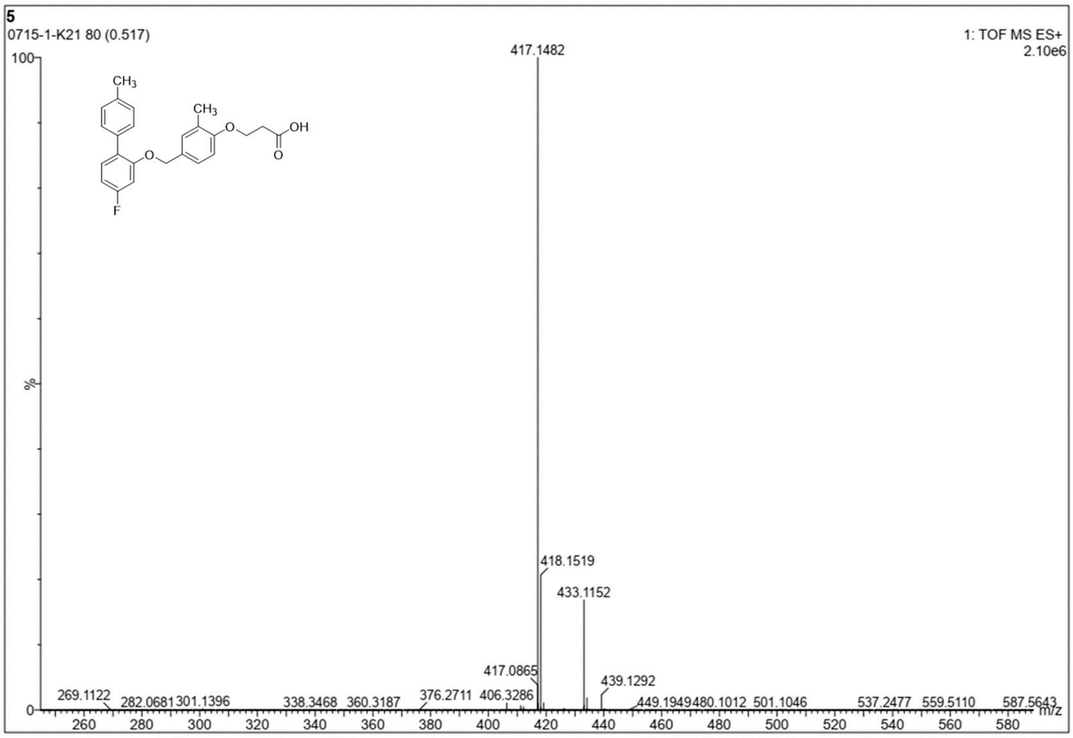

Figure S20. HRMS spectrum **10f.**

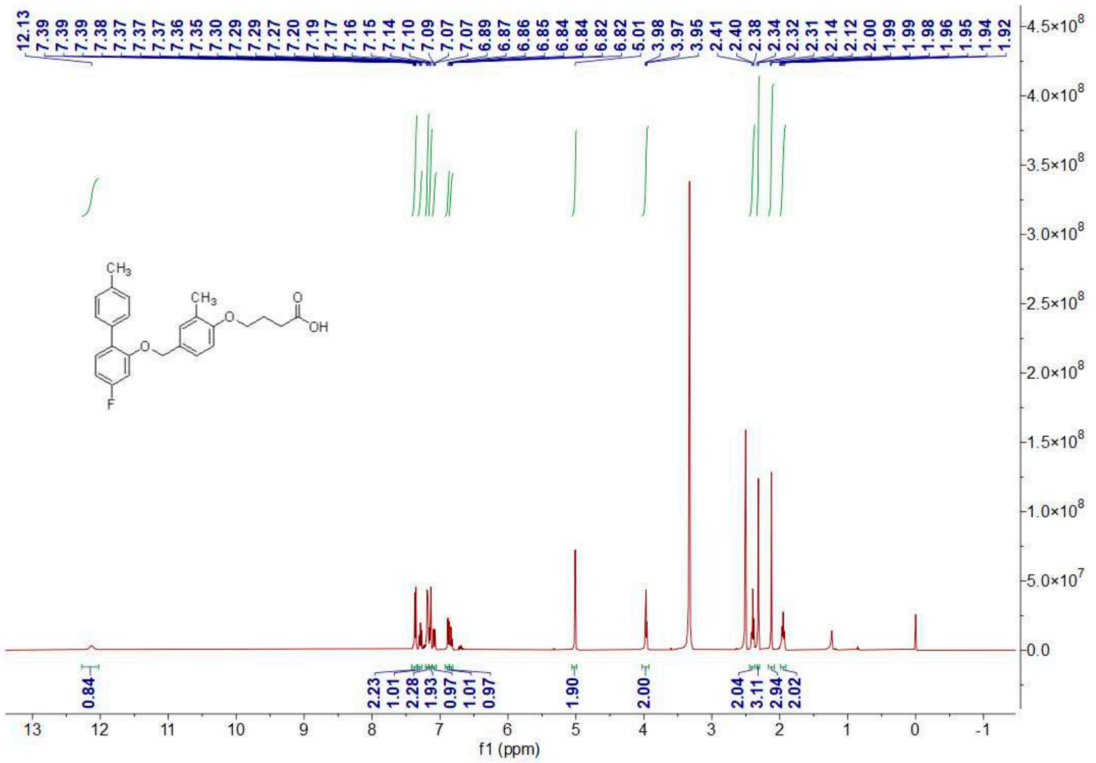

Figure S21. ^{1}H NMR spectrum **10g.**

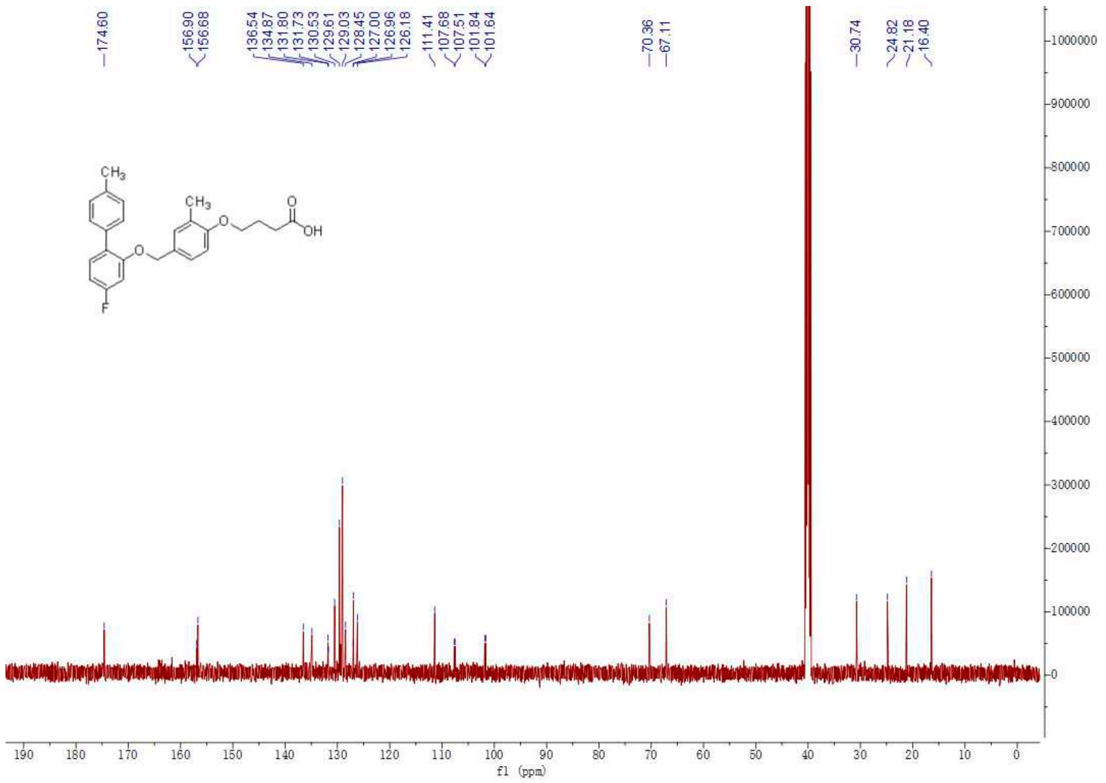

Figure S22. ^{13}C NMR spectrum **10g**.

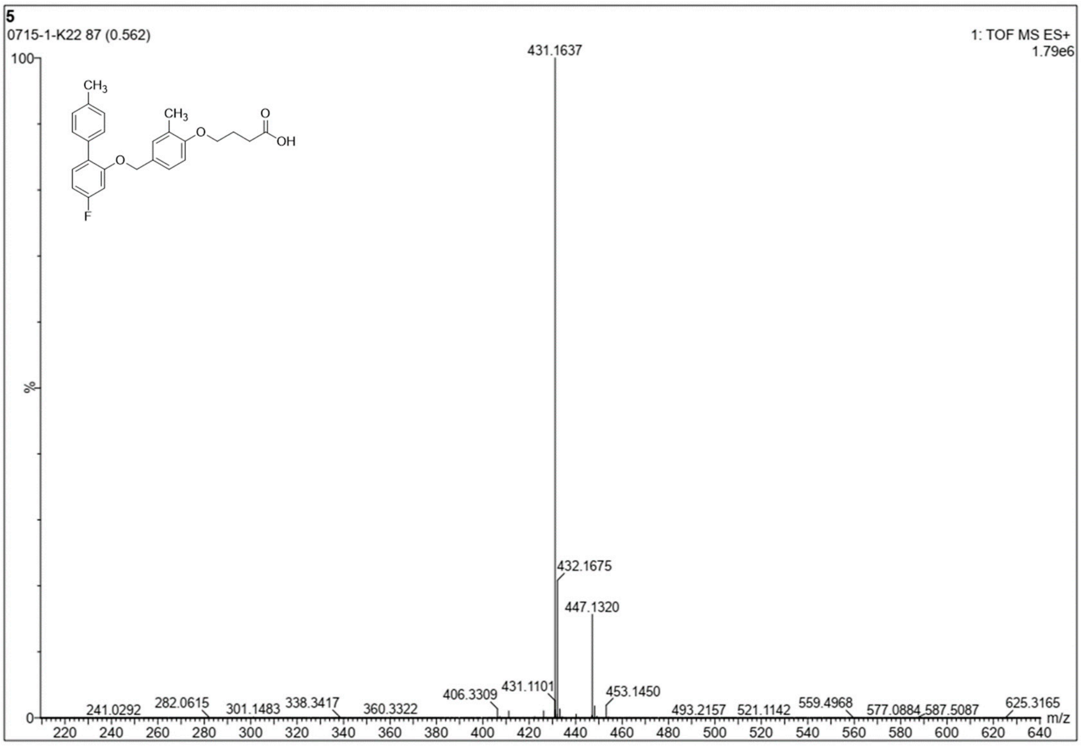

Figure S23. HRMS spectrum **10g.**

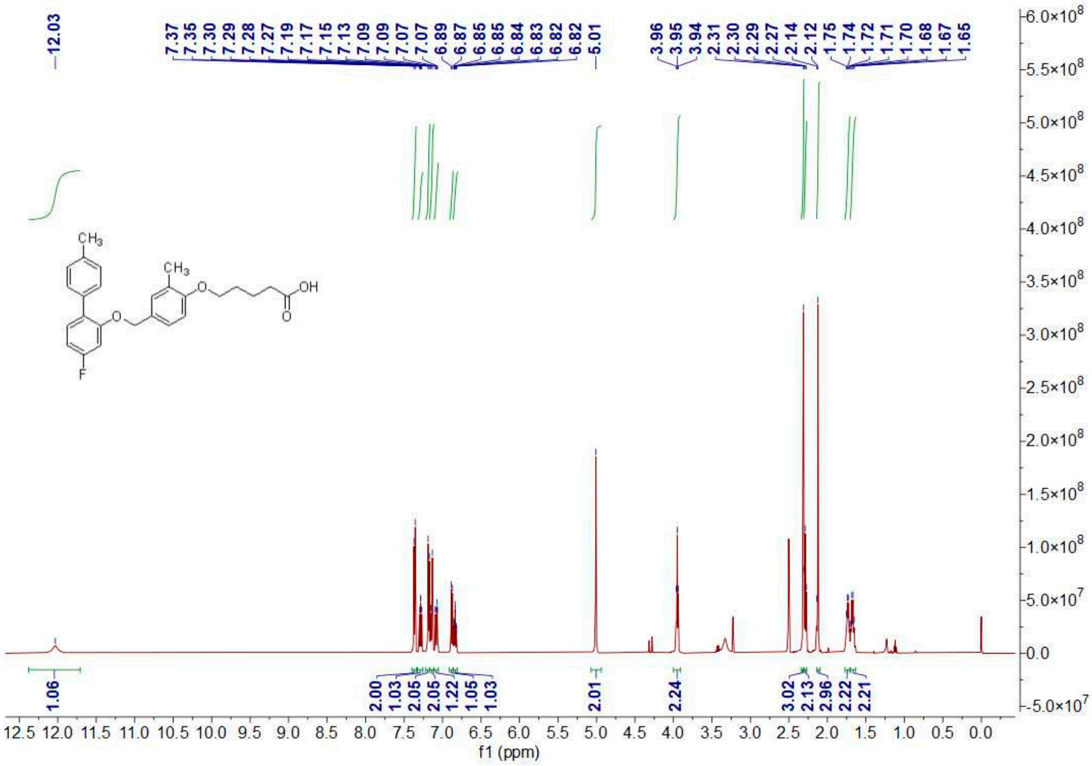

Figure S24. ^{1}H NMR spectrum **10h.**

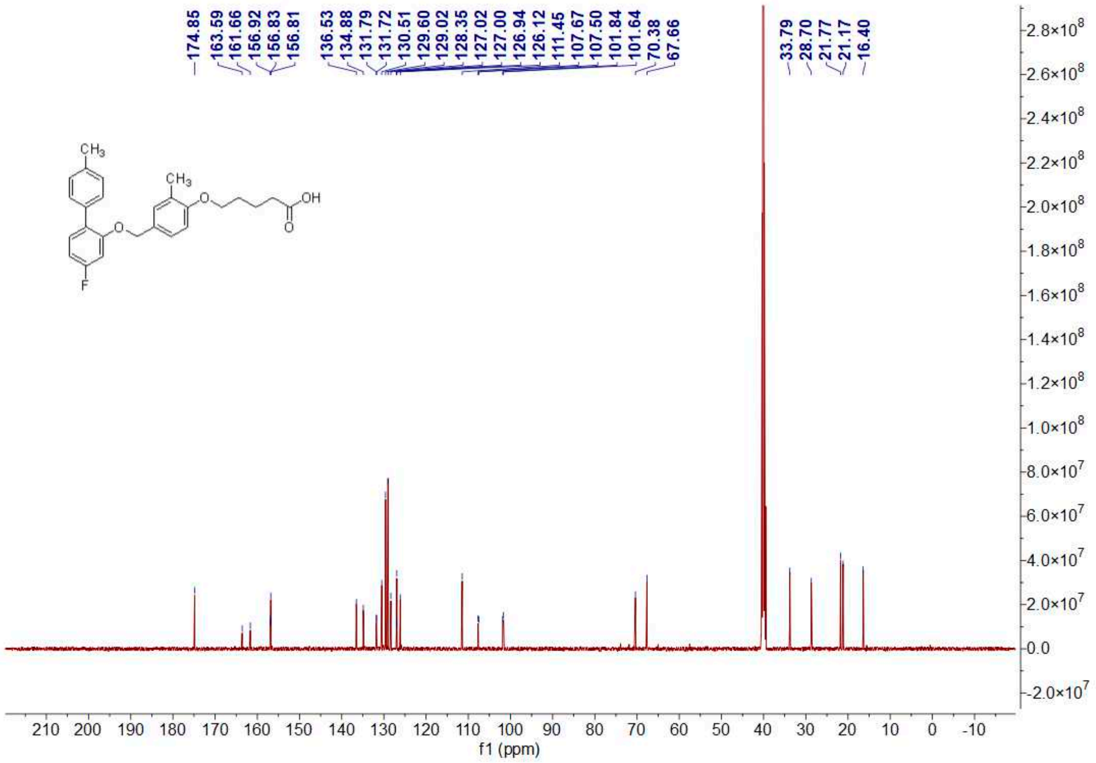

Figure S25. ${}^{13}C$ NMR spectrum **10h**.

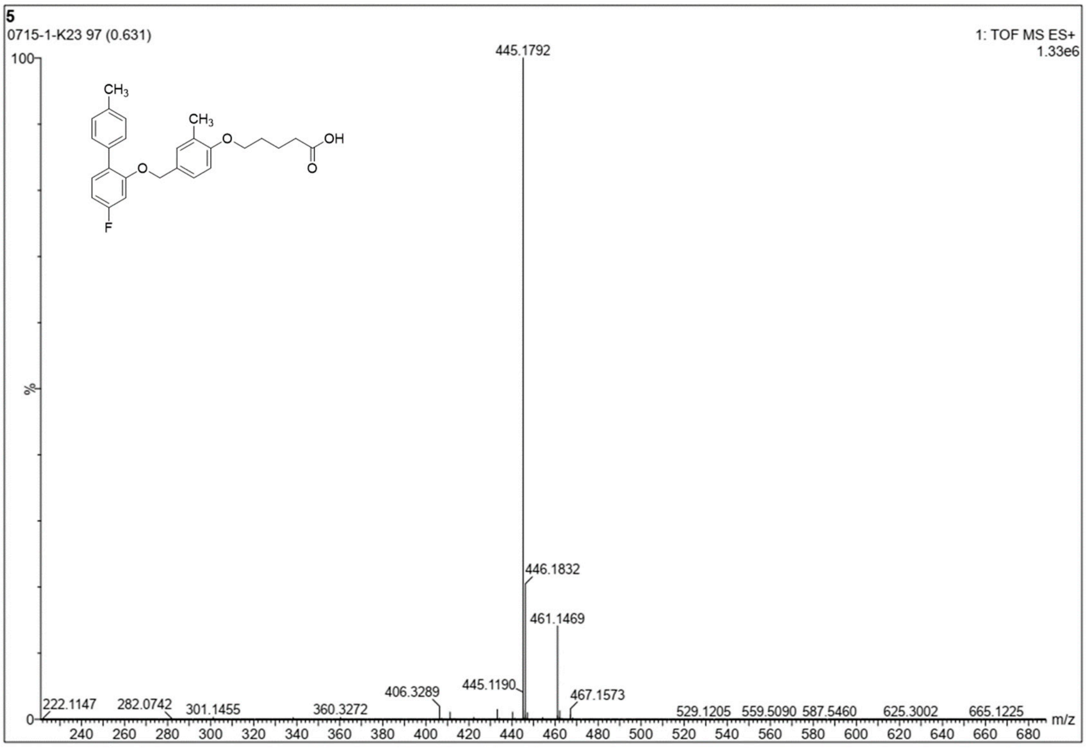

Figure S26. HRMS spectrum **10h.**

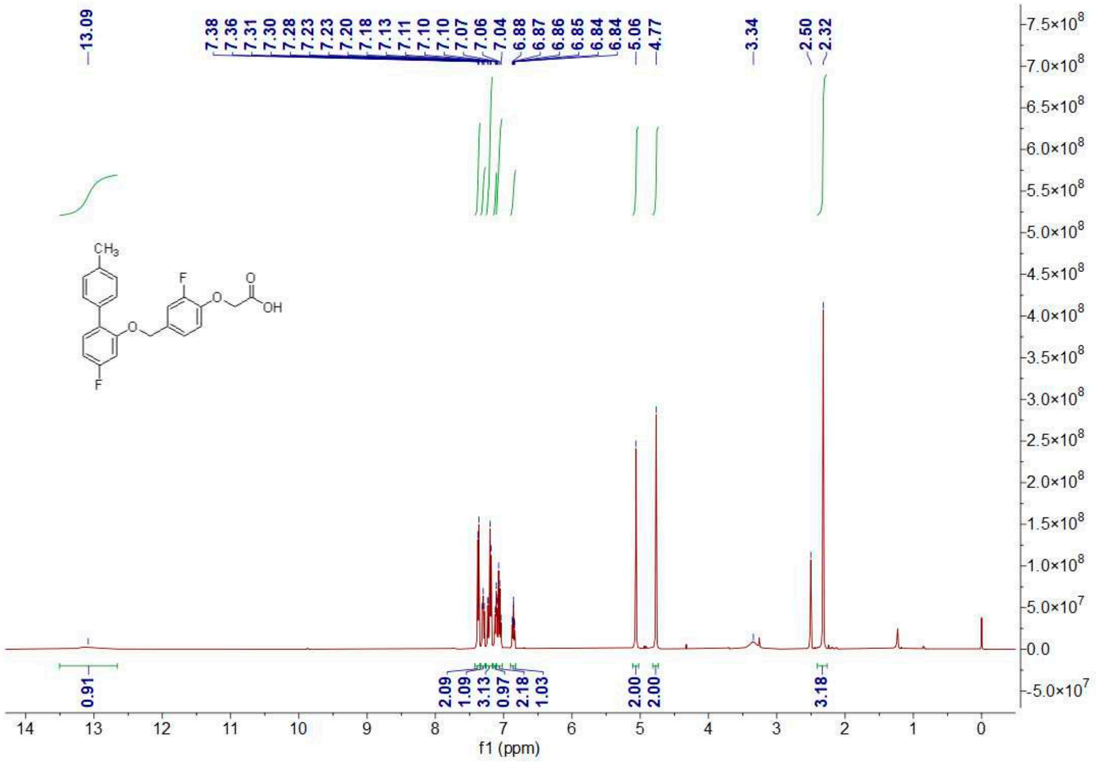

Figure S27. ^{1}H NMR spectrum **10i.**

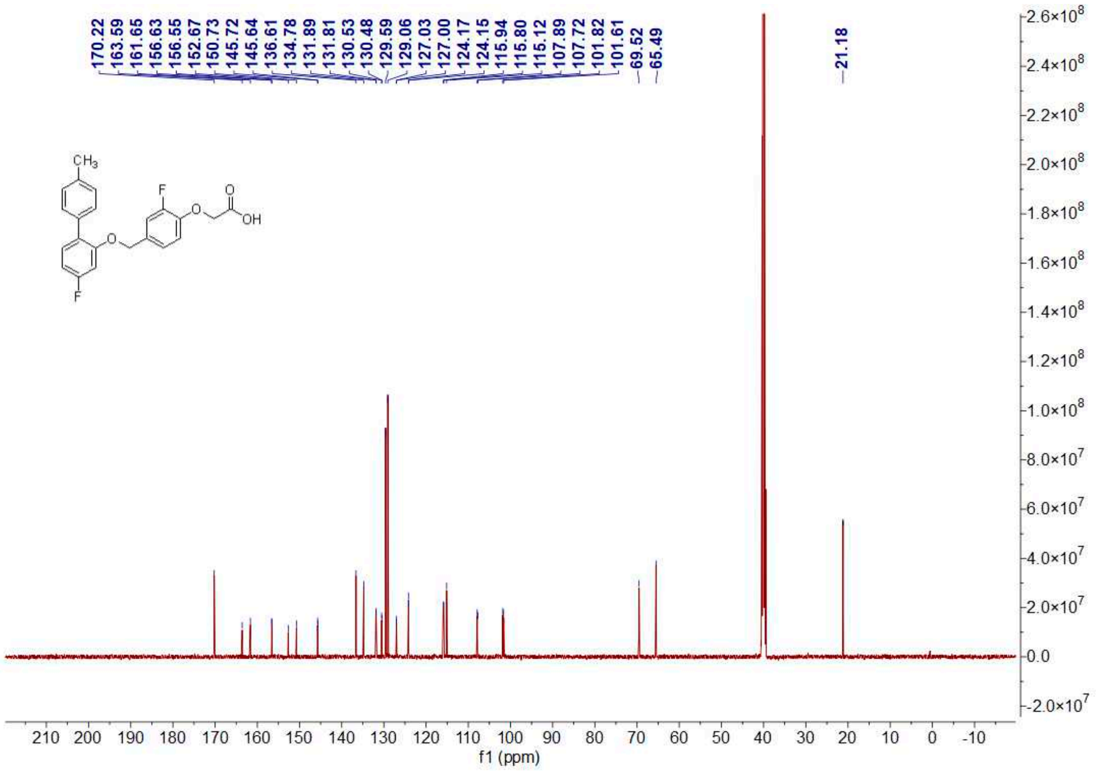

Figure S28. ^{13}C NMR spectrum **10i**.

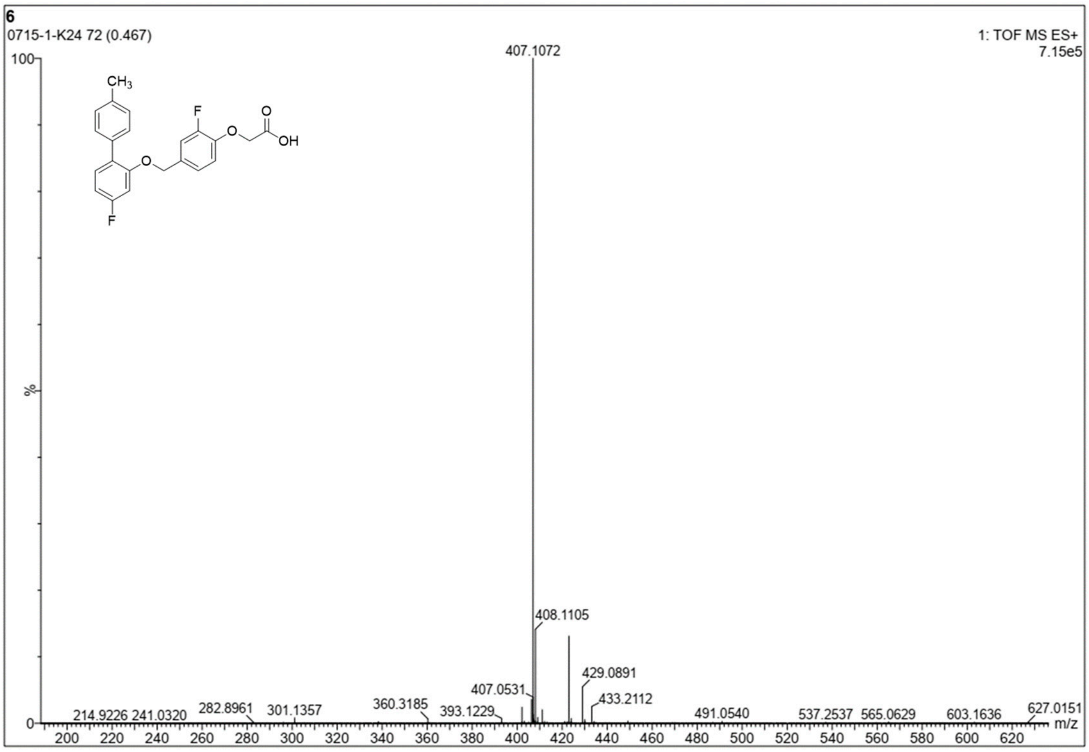

Figure S29. HRMS spectrum **10i.**

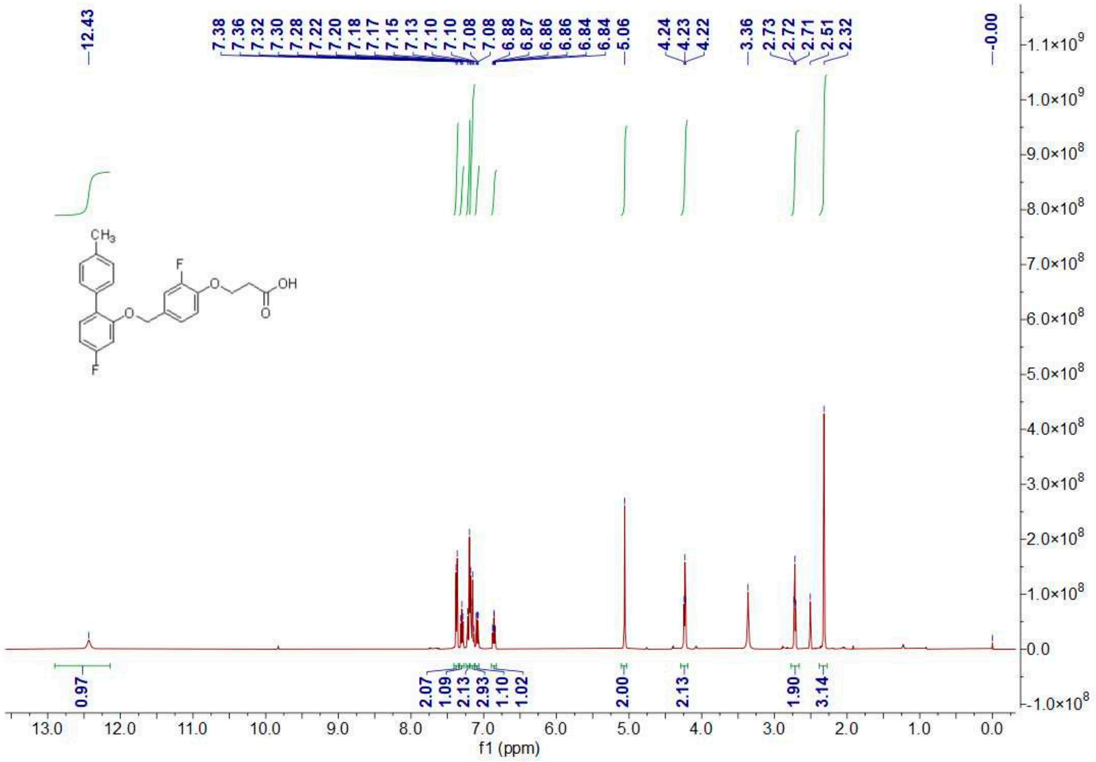

Figure S30. ^{1}H NMR spectrum **10j.**

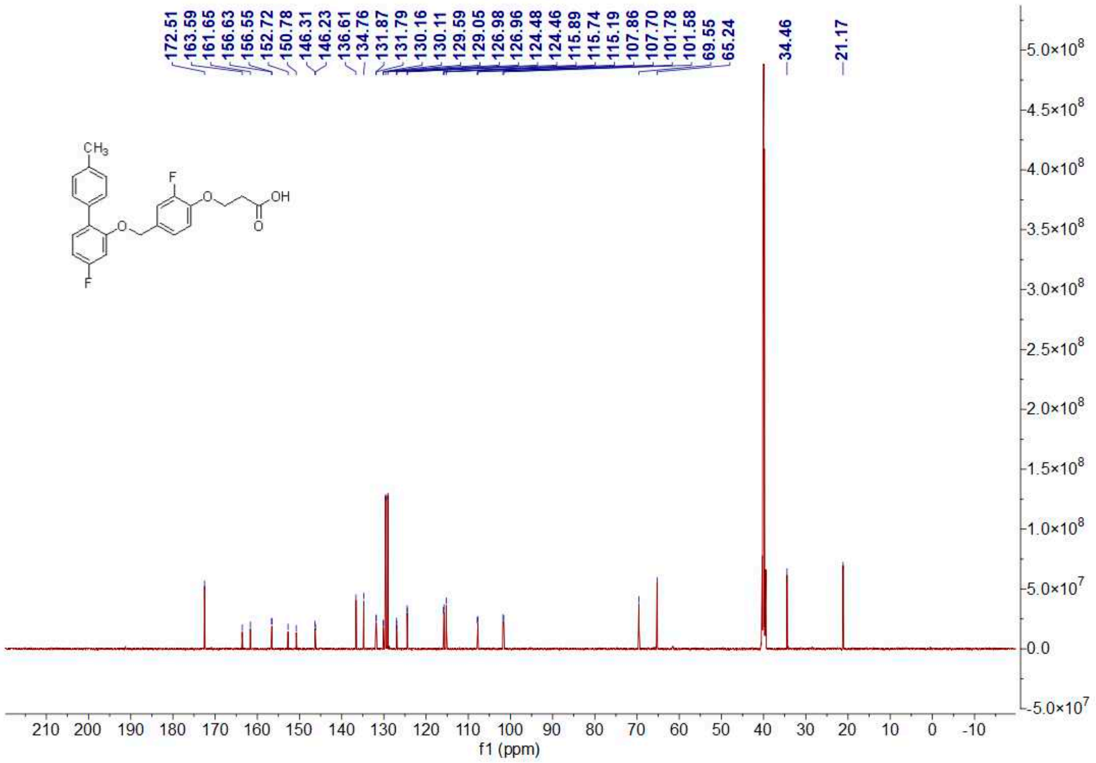

Figure S31. ^{13}C NMR spectrum **10j**.

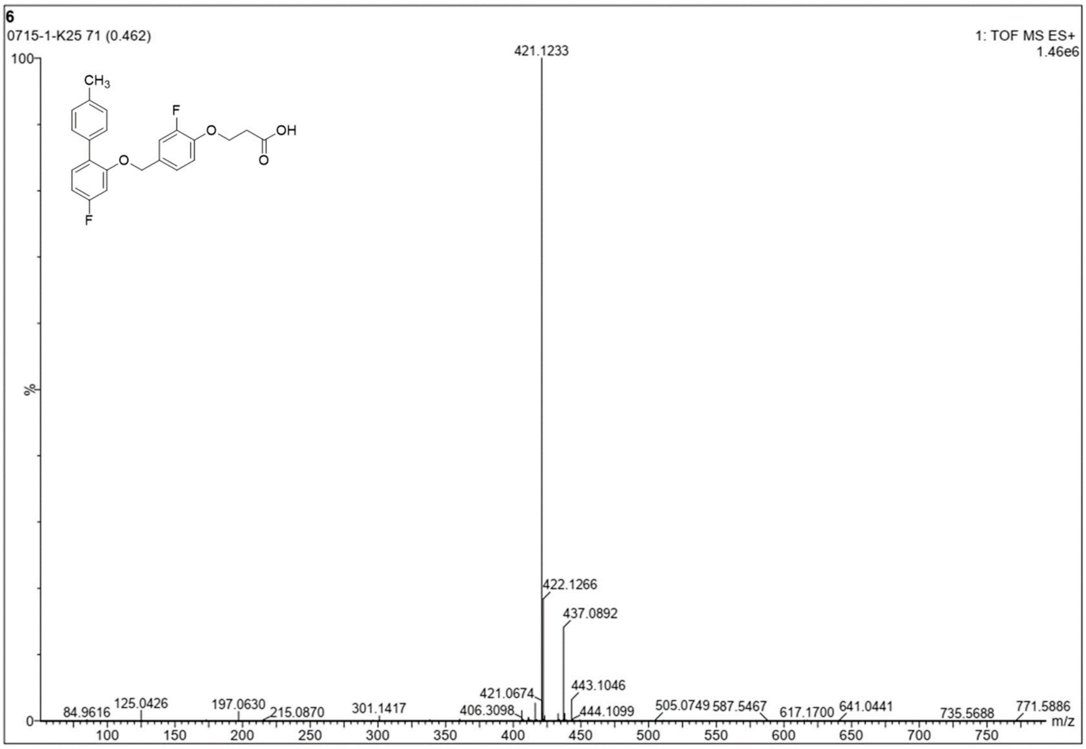

Figure S32. HRMS spectrum **10j.**

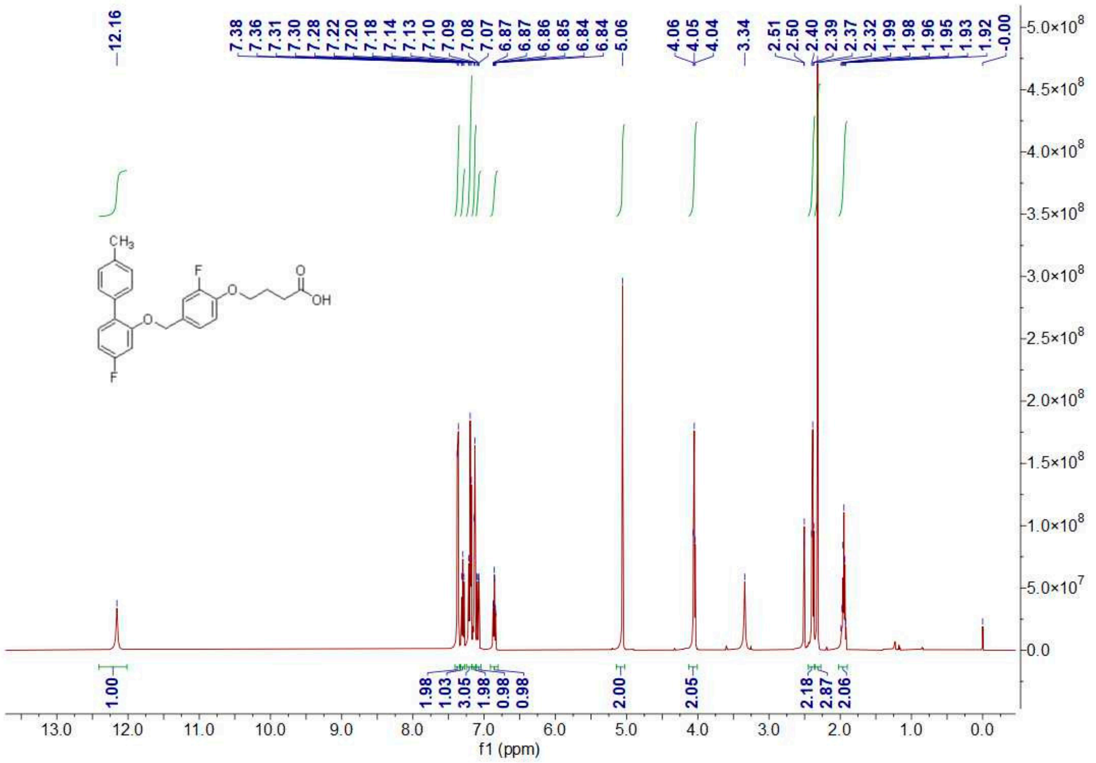

Figure S33. ^{1}H NMR spectrum **10k.**

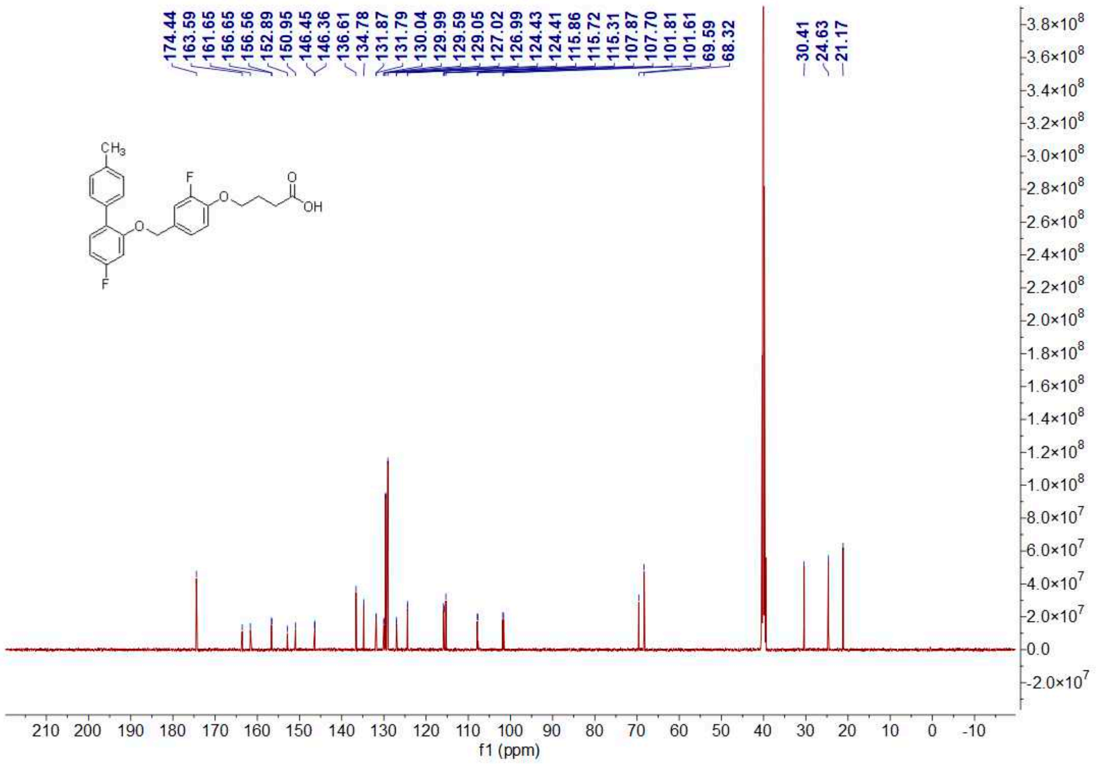

Figure S34. ^{13}C NMR spectrum **10k**.

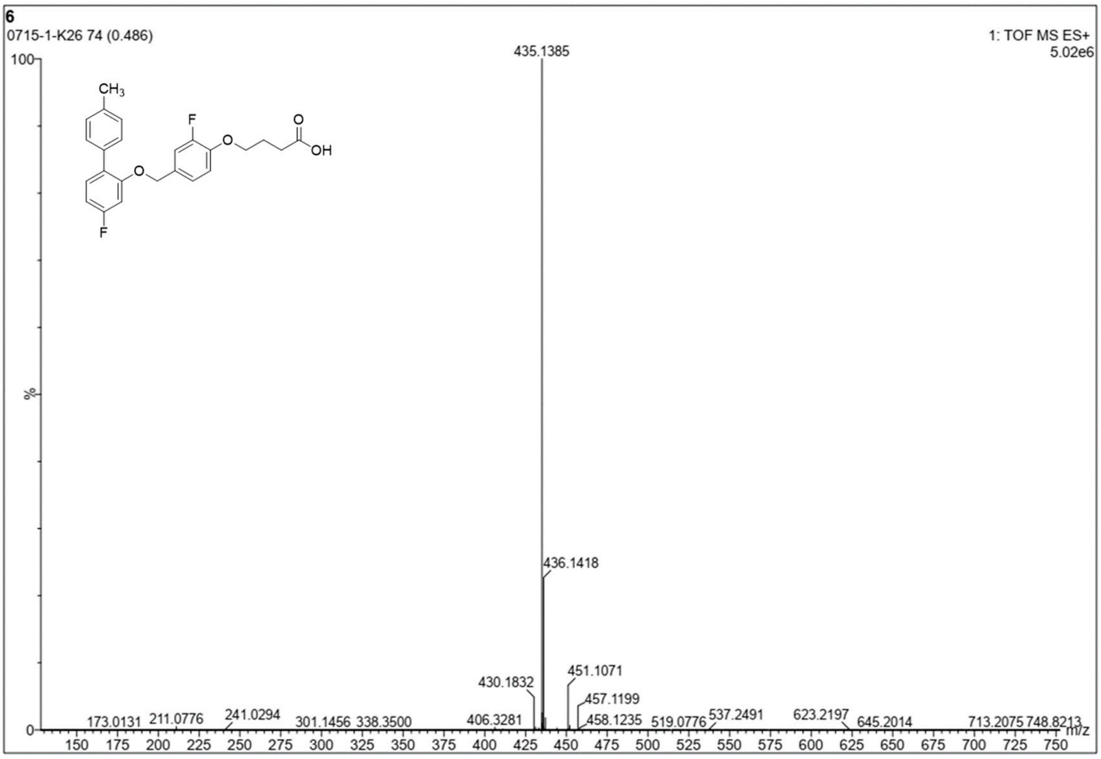

Figure S35. HRMS spectrum **10k.**

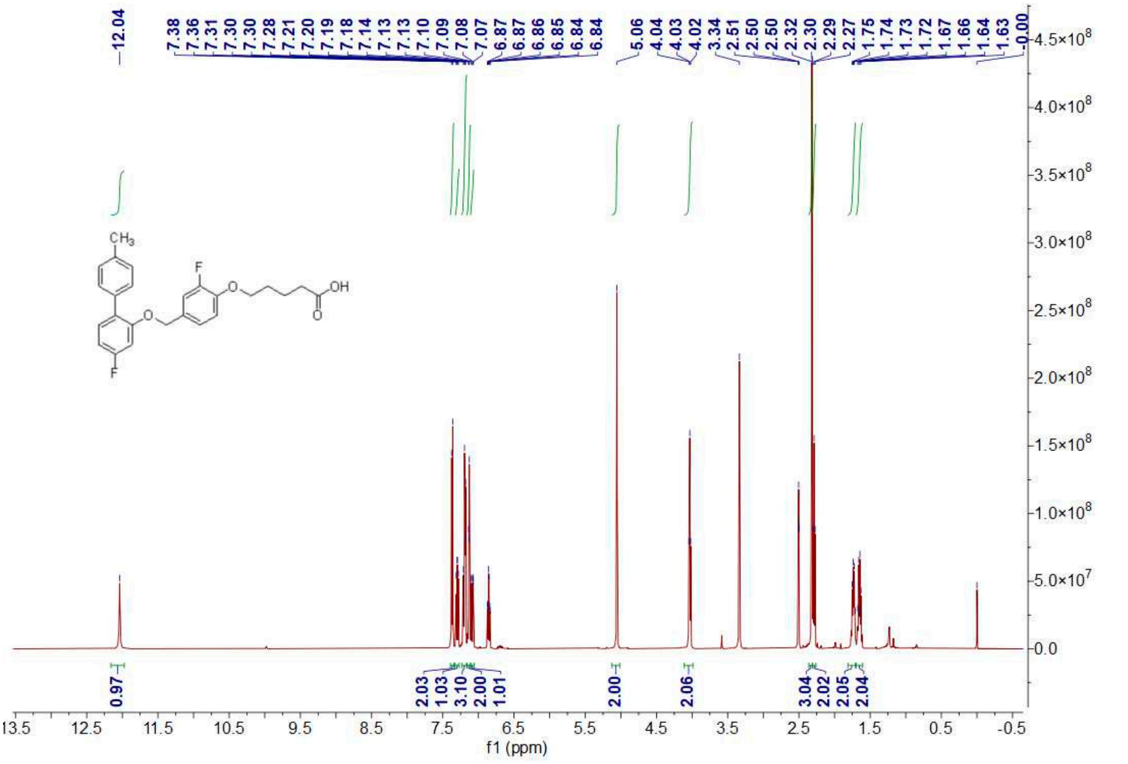

Figure S36. ^{1}H NMR spectrum **10l.**

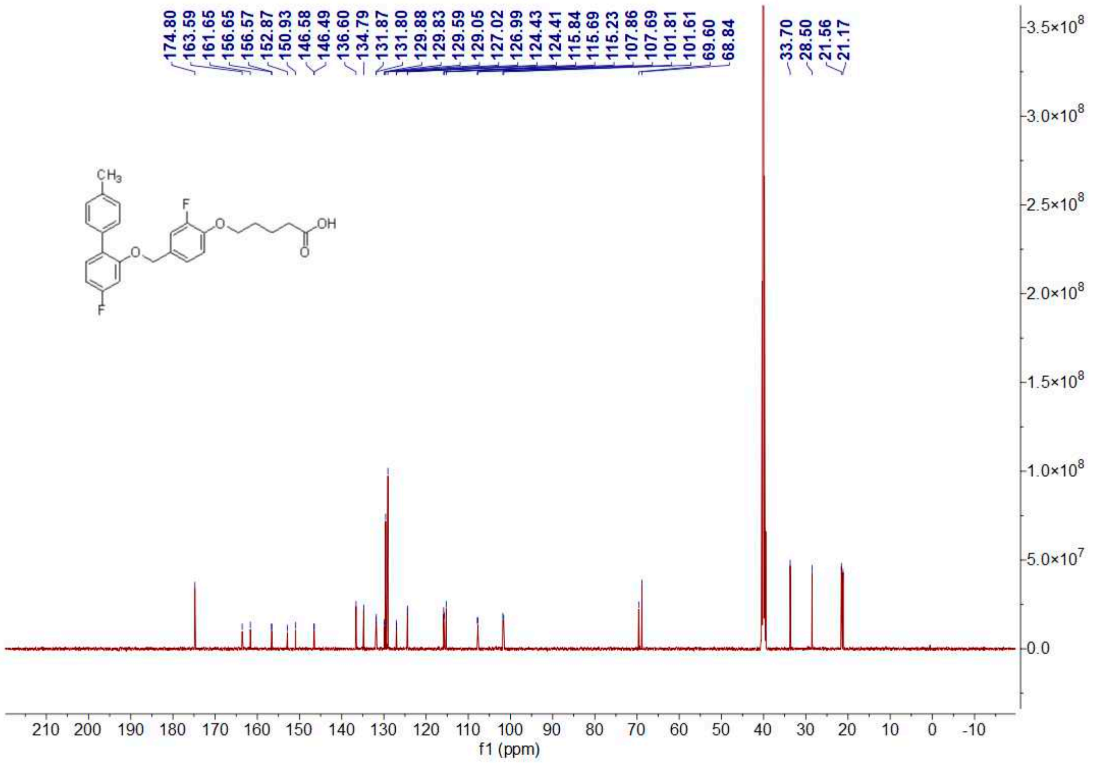

Figure S37. ^{13}C NMR spectrum **10l**.

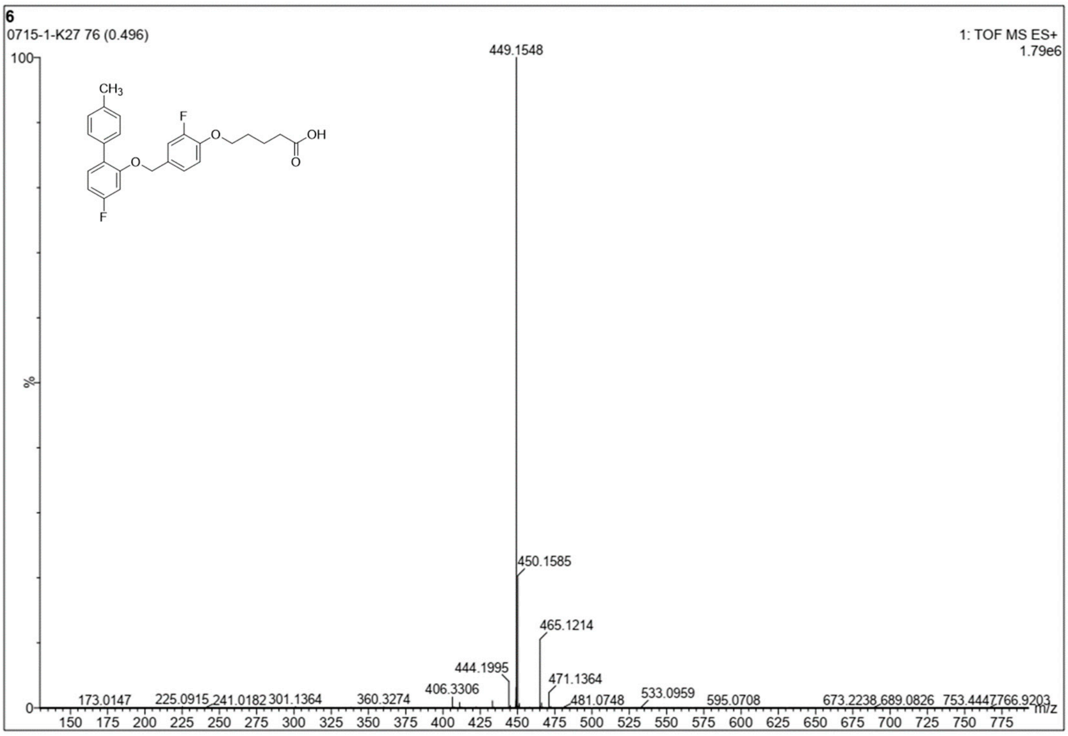

Figure S38. HRMS spectrum **10l.**

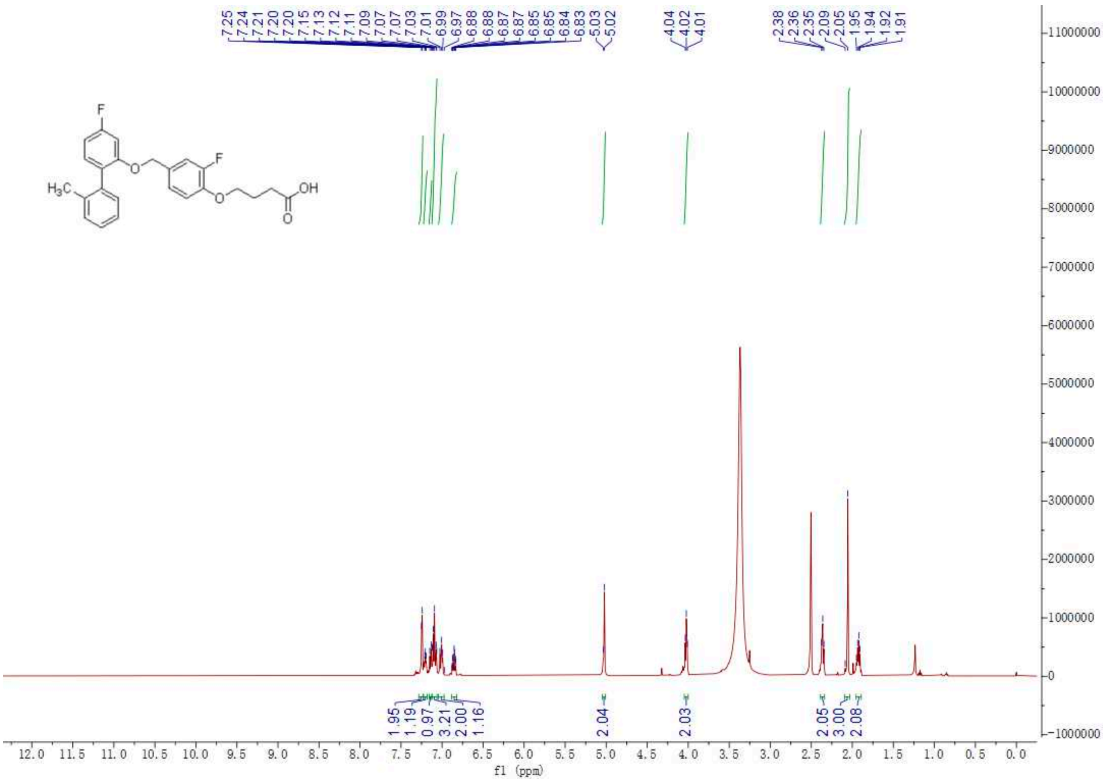

Figure S39. ^{1}H NMR spectrum **14a.**

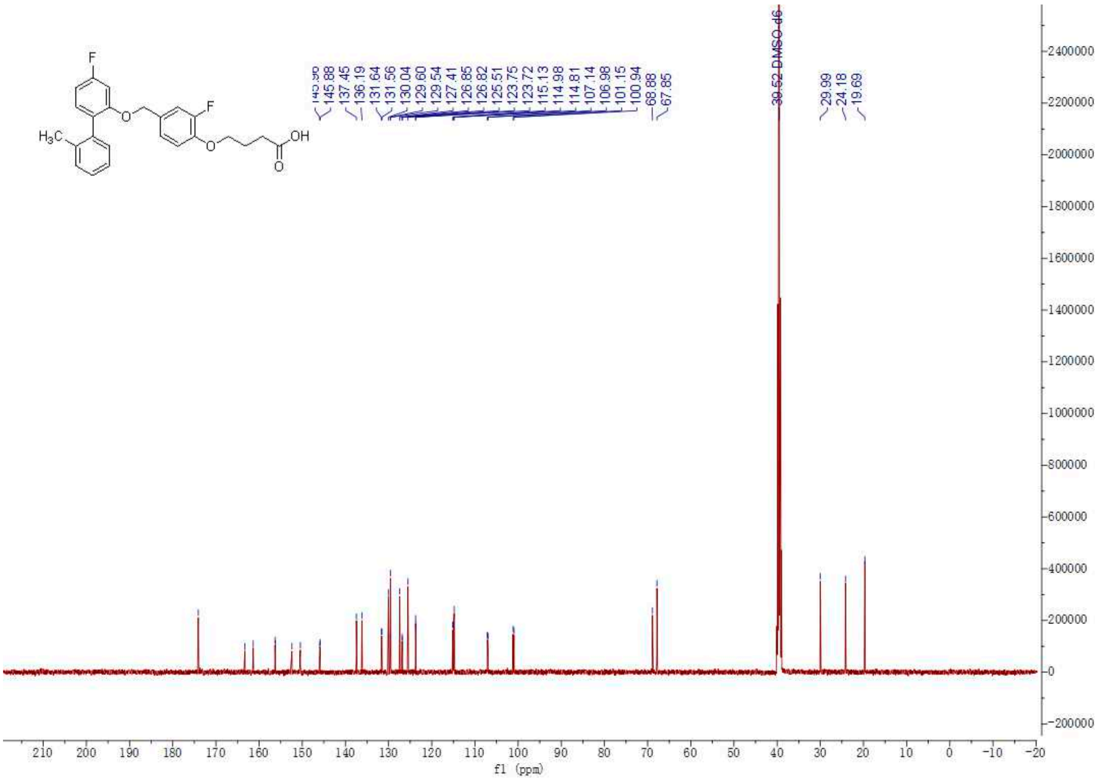

Figure S40. ^{13}C NMR spectrum **14a**.

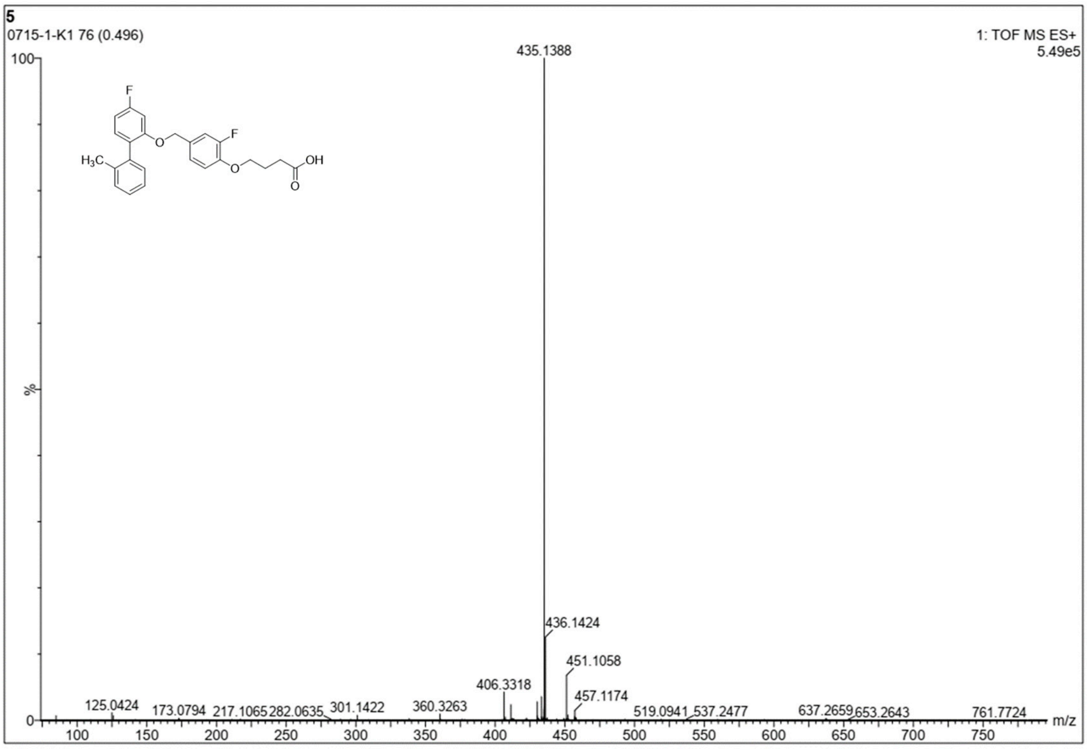

Figure S41. HRMS spectrum **14a.**

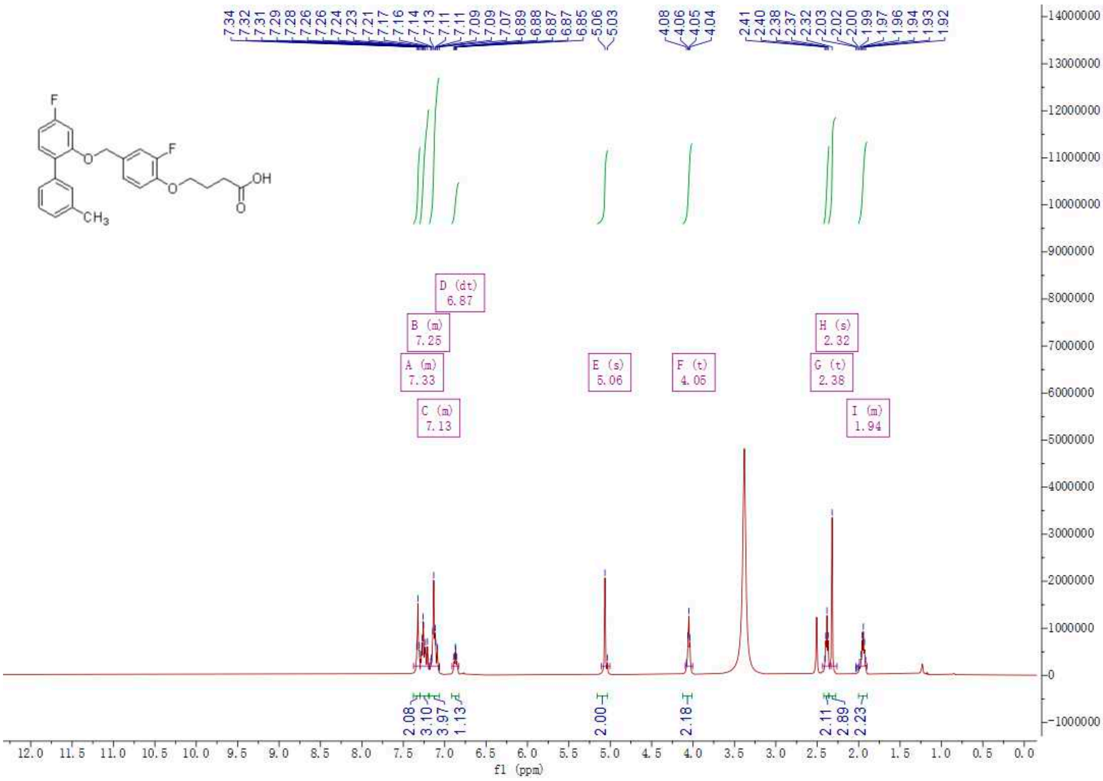

Figure S42. ^{1}H NMR spectrum **14b.**

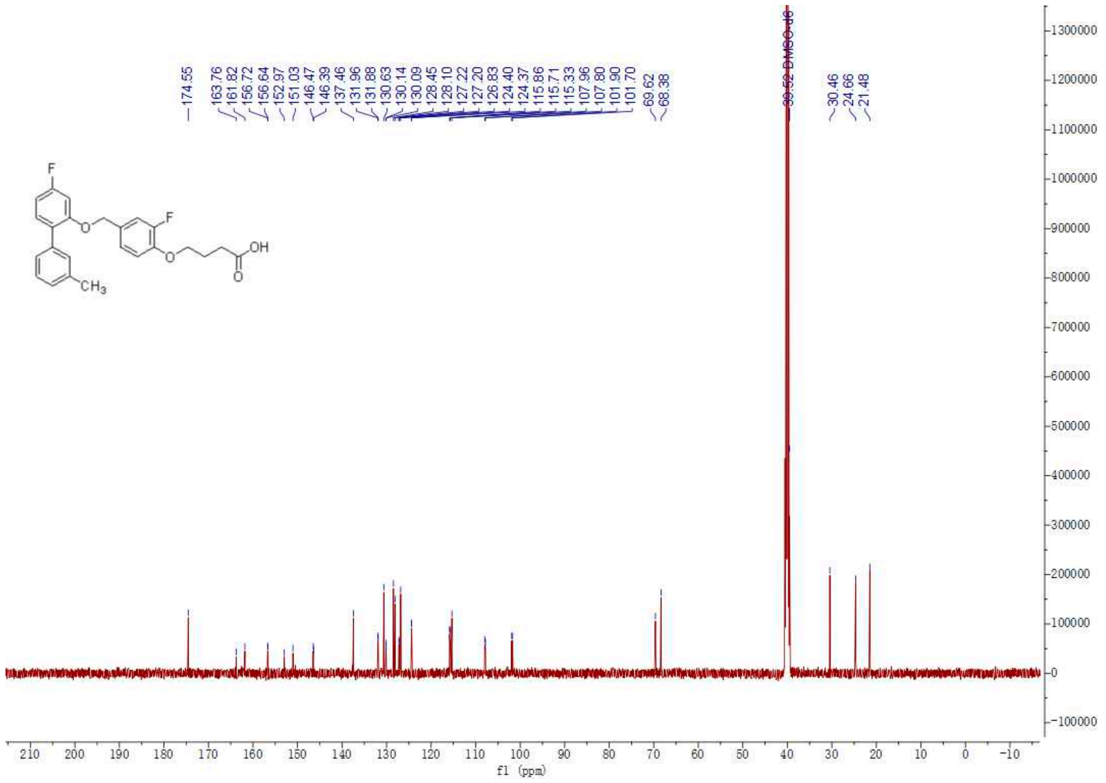

Figure S43. ^{13}C NMR spectrum **14b**.

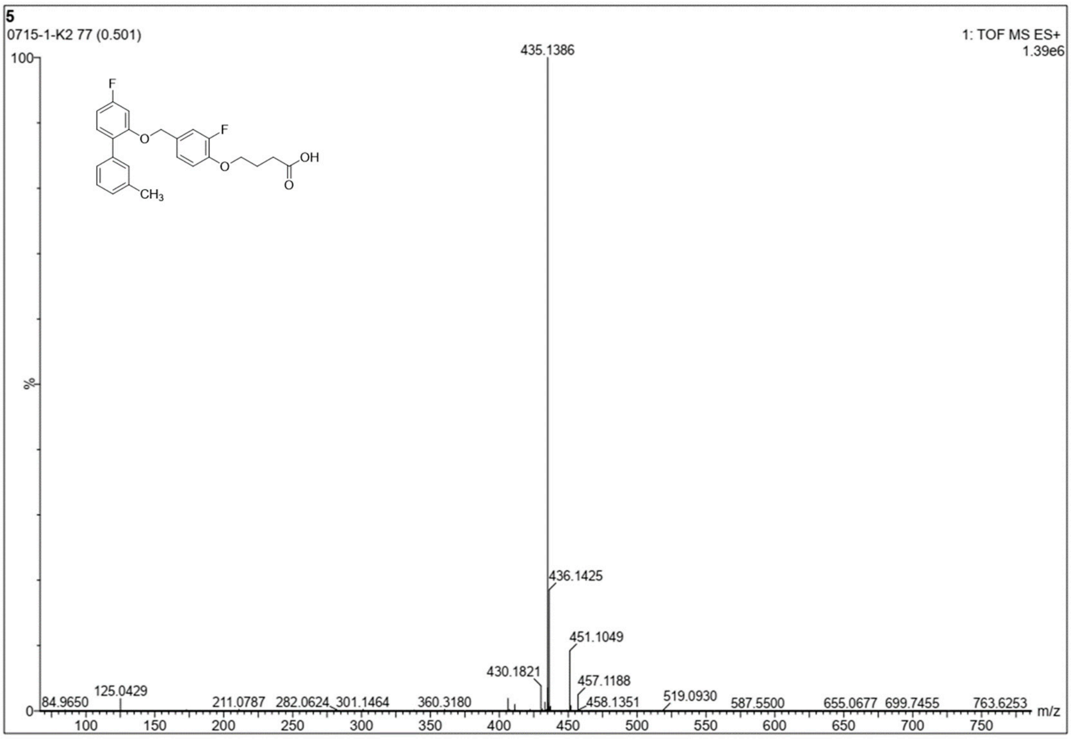

Figure S44. HRMS spectrum **14b.**

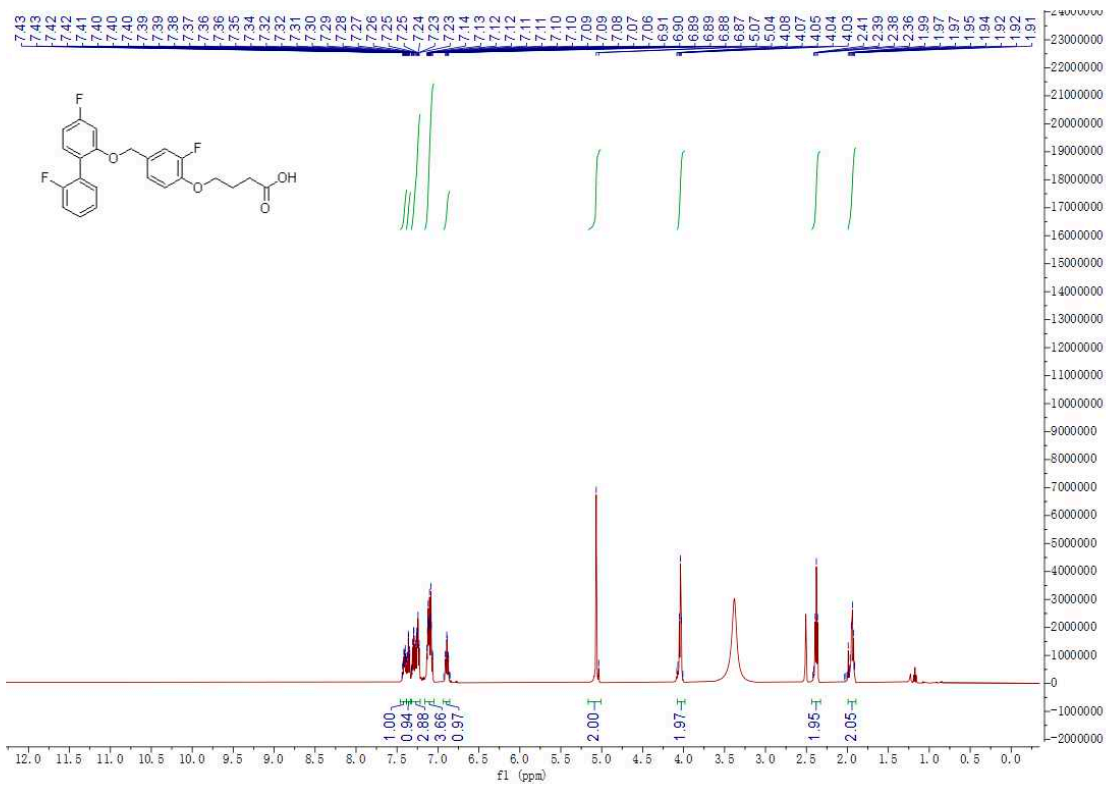

Figure S45. ^{1}H NMR spectrum **14c.**

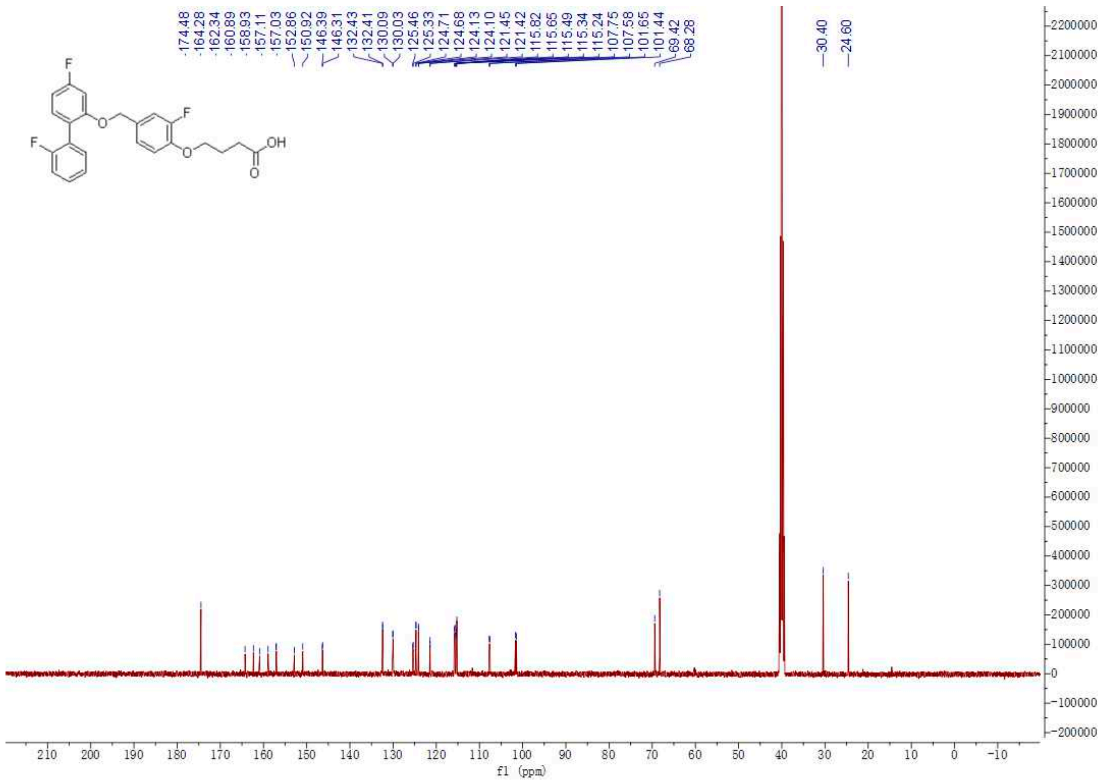

Figure S46. ^{13}C NMR spectrum **14c**.

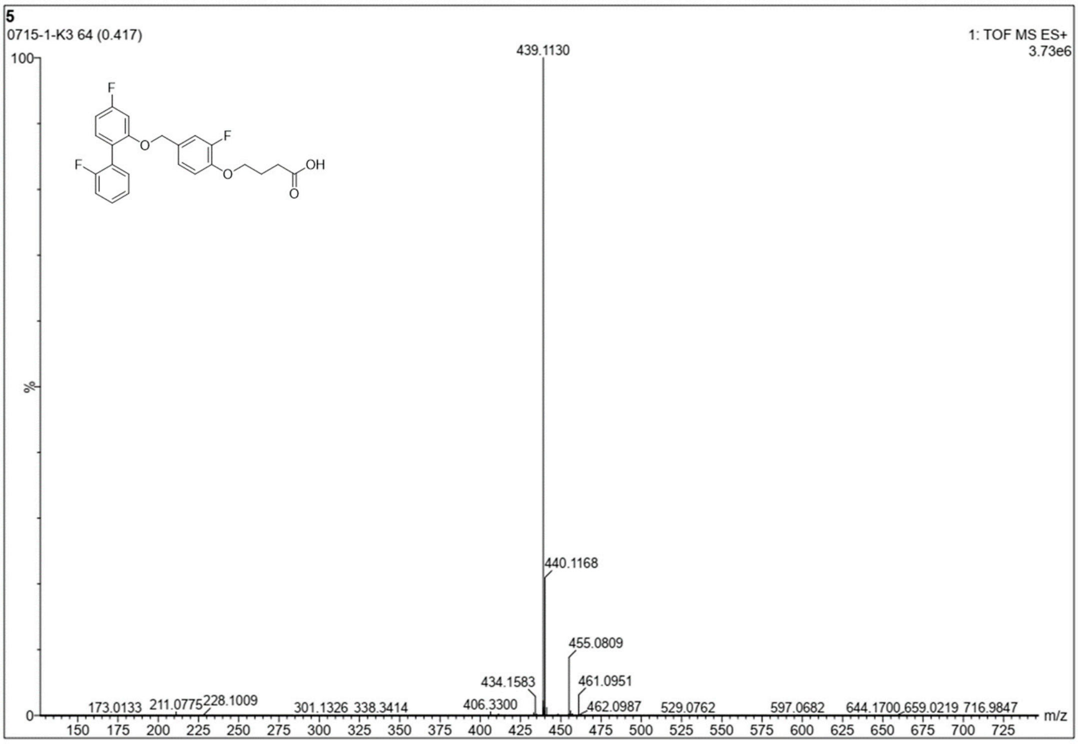

Figure S47. HRMS spectrum **14c.**

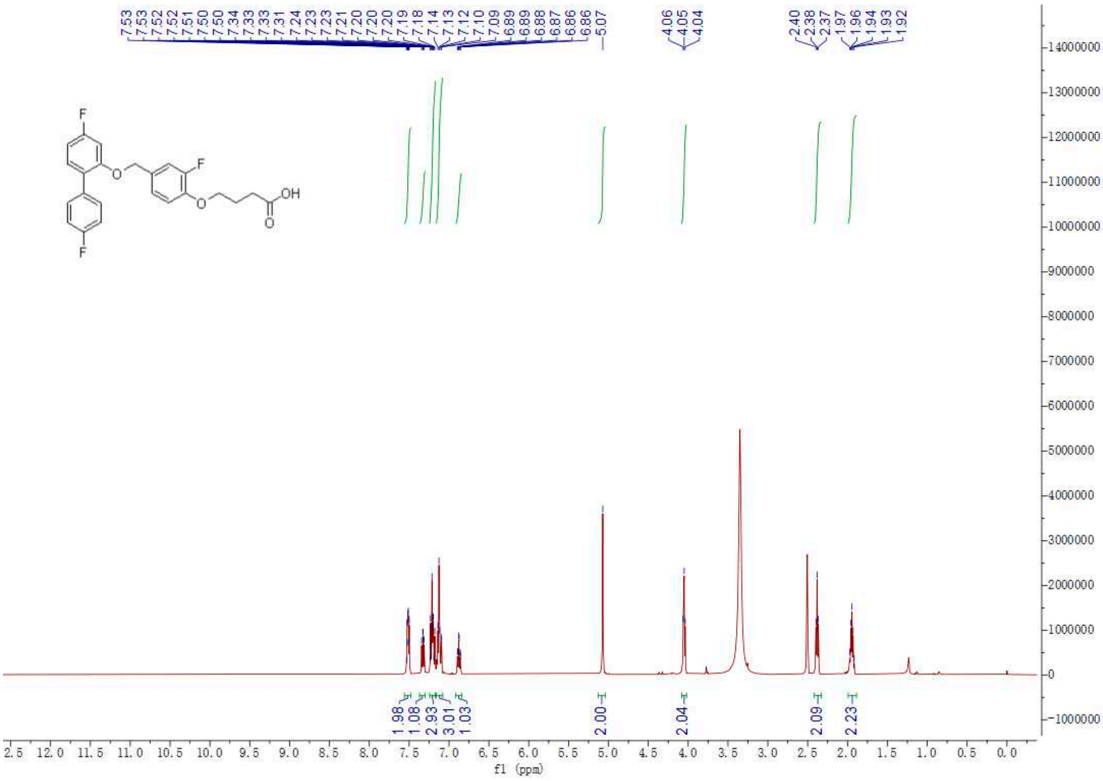

Figure S48. ^{1}H NMR spectrum **14d.**

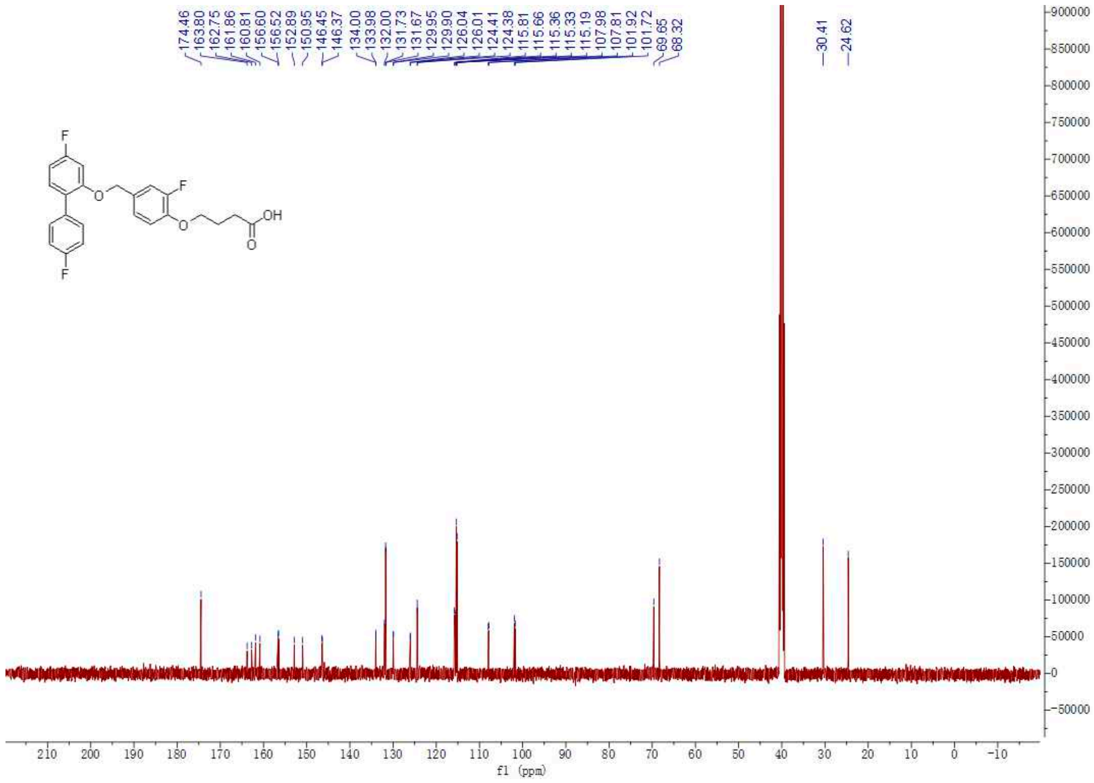

Figure S49. ^{13}C NMR spectrum **14d**.

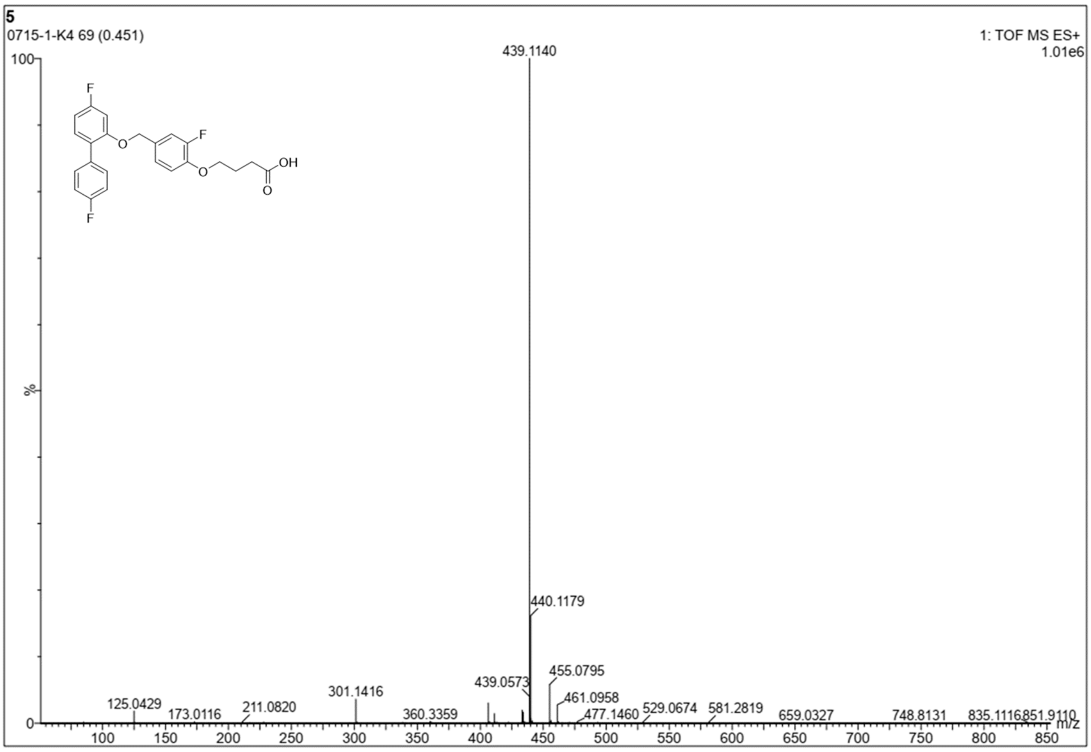

Figure S50. HRMS spectrum **14d.**

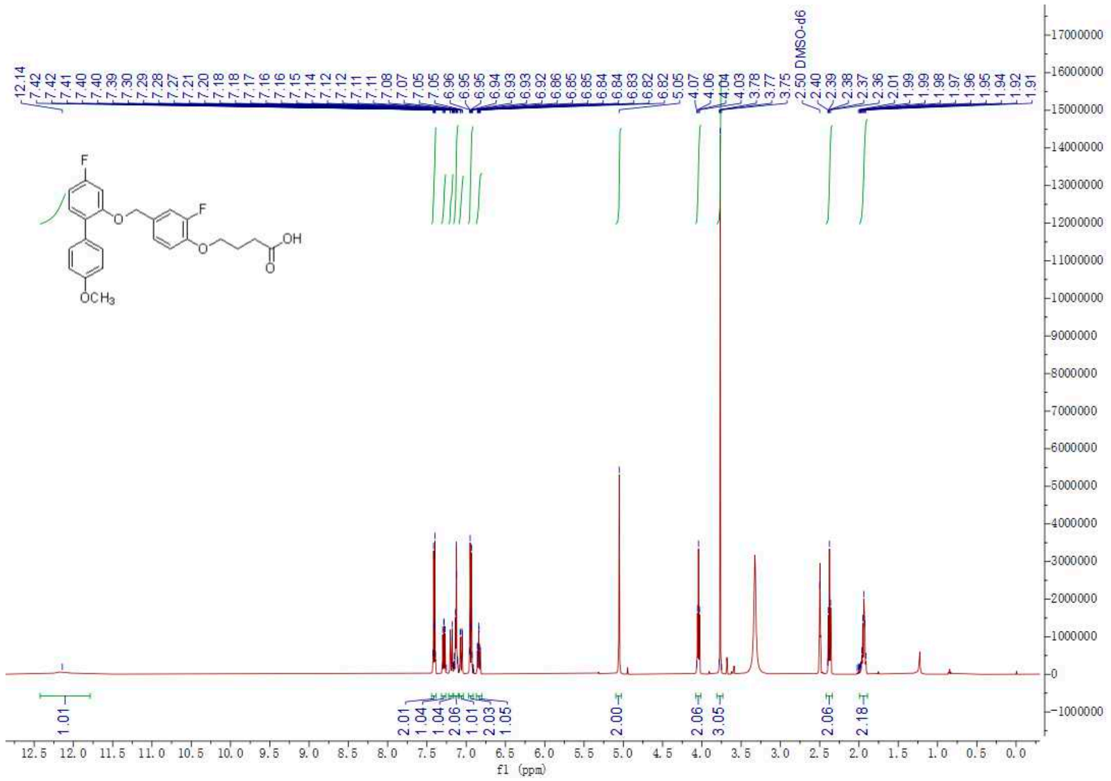

Figure S51. ^{1}H NMR spectrum **14e.**

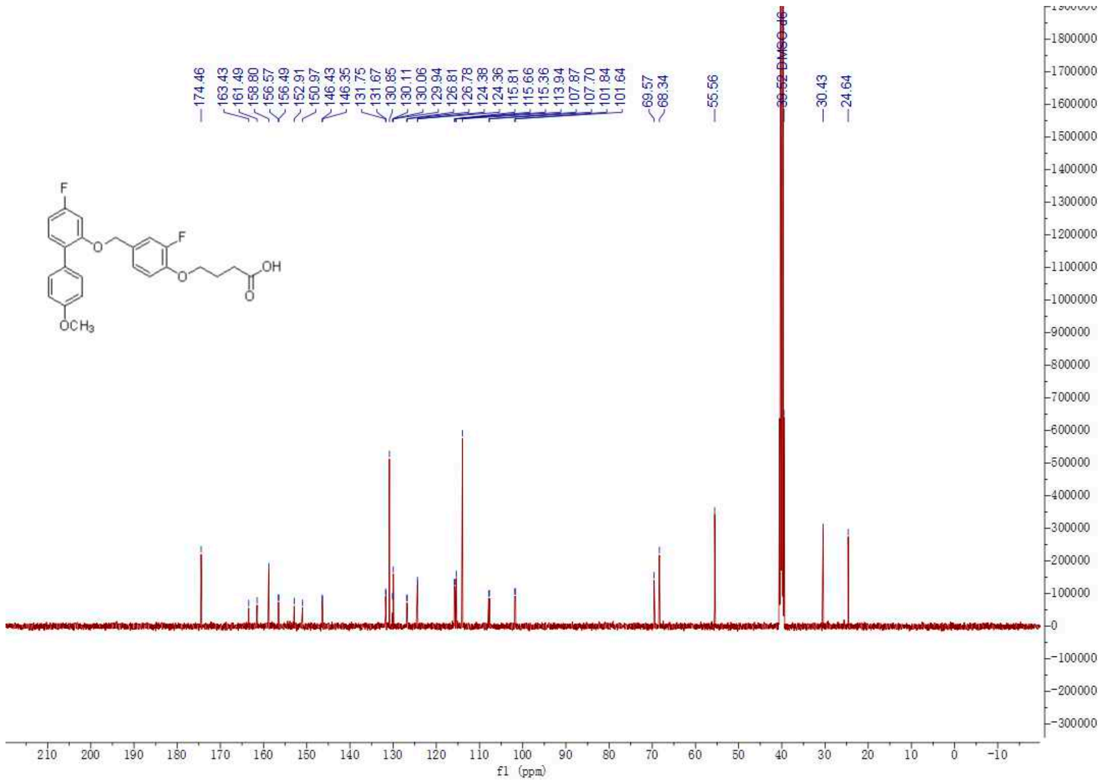

Figure S52. ^{13}C NMR spectrum **14e**.

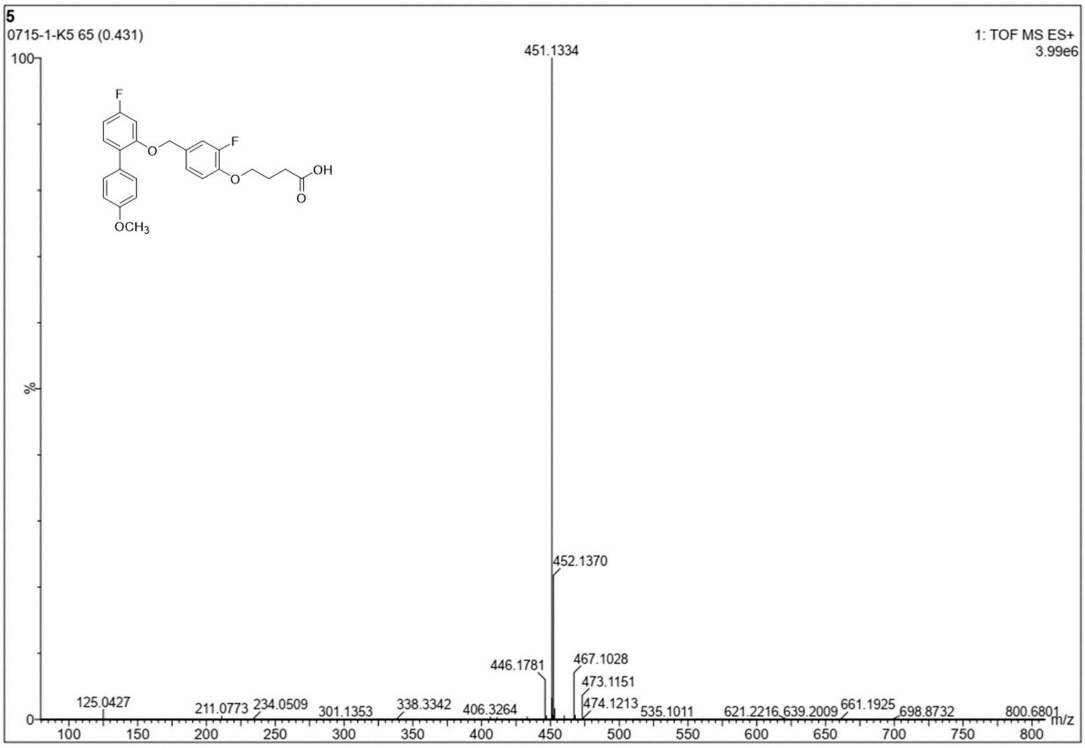

Figure S53. HRMS spectrum **14e.**

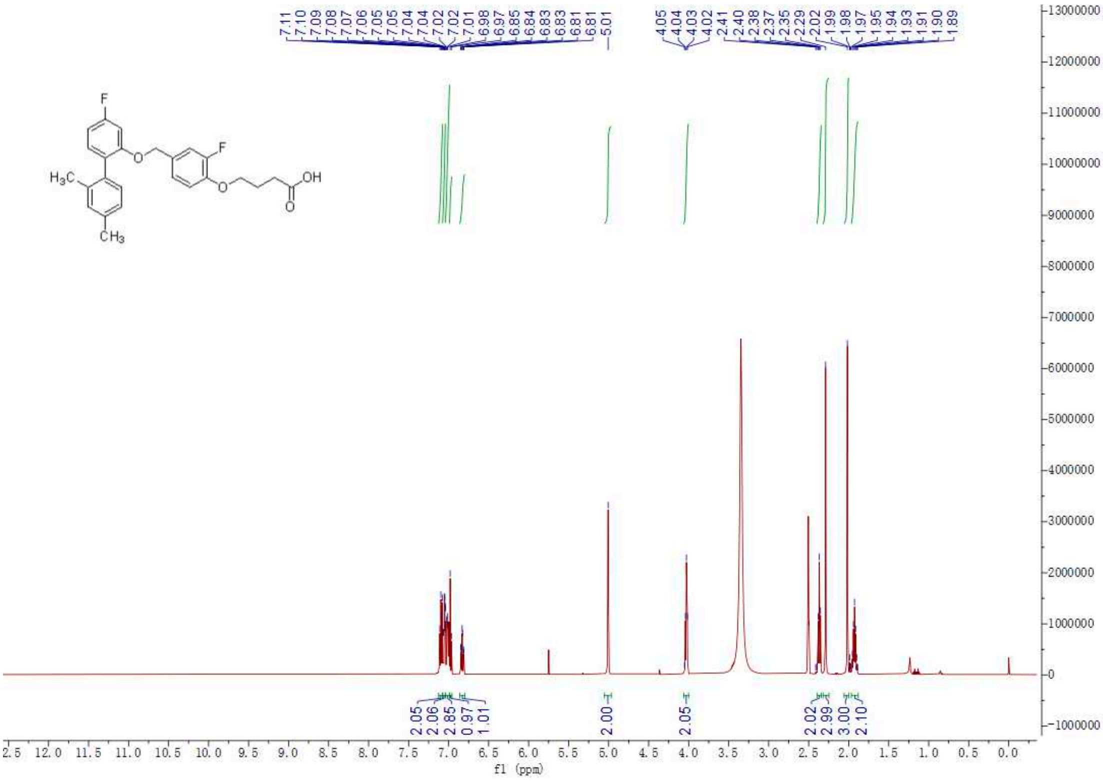

Figure S54. ^{1}H NMR spectrum **14f.**

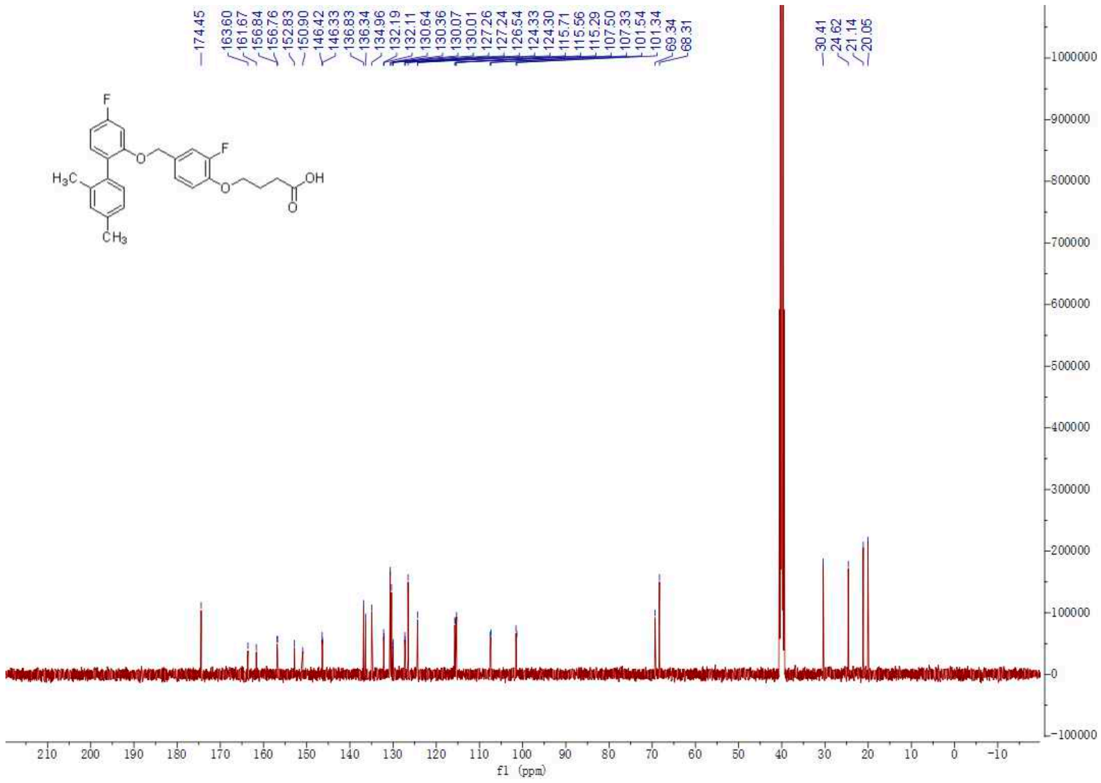

Figure S55. ^{13}C NMR spectrum **14f**.

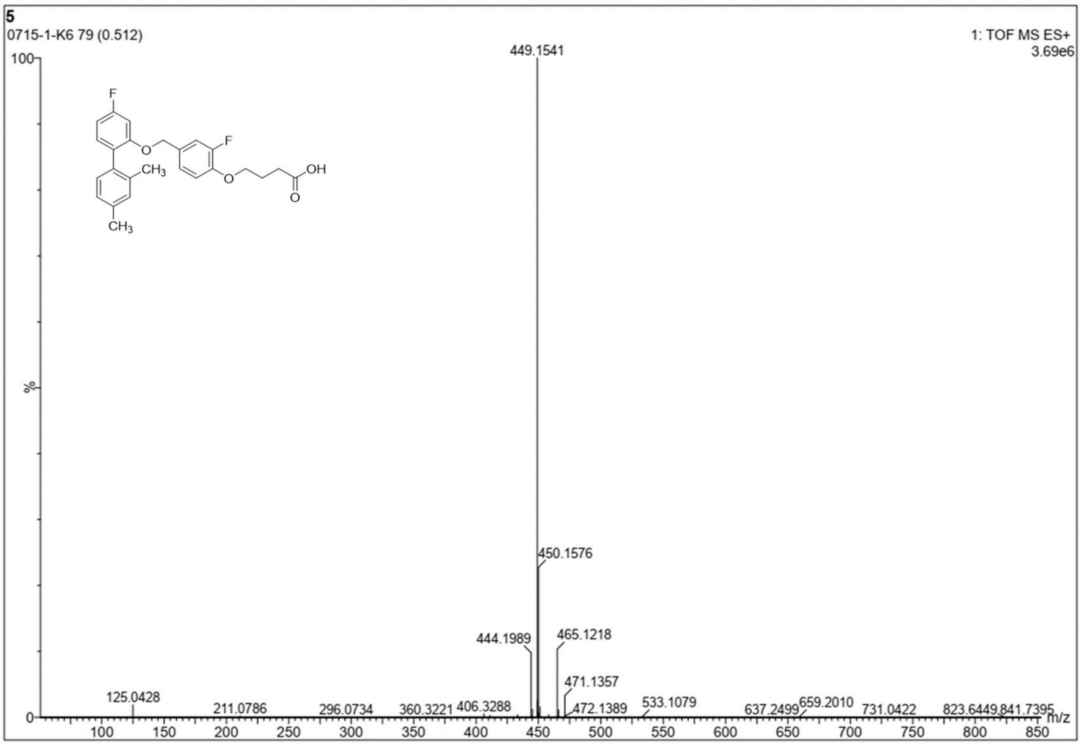

Figure S56. HRMS spectrum **14f.**

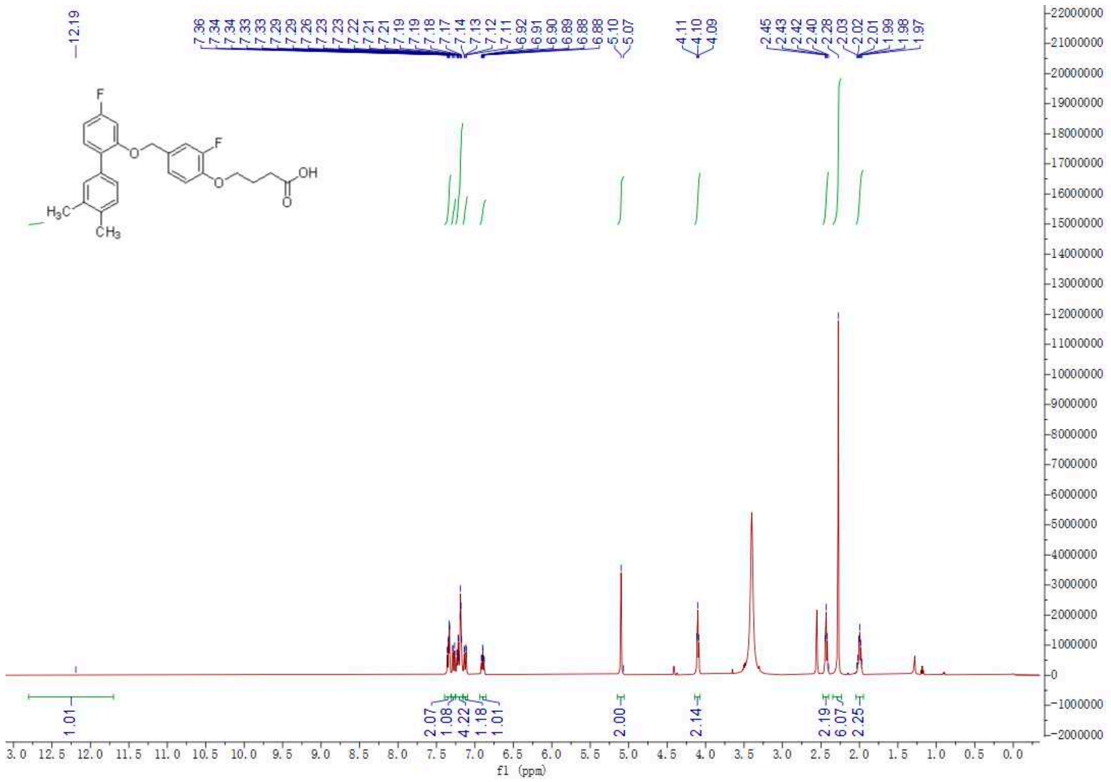

Figure S57. ^{1}H NMR spectrum **14g.**

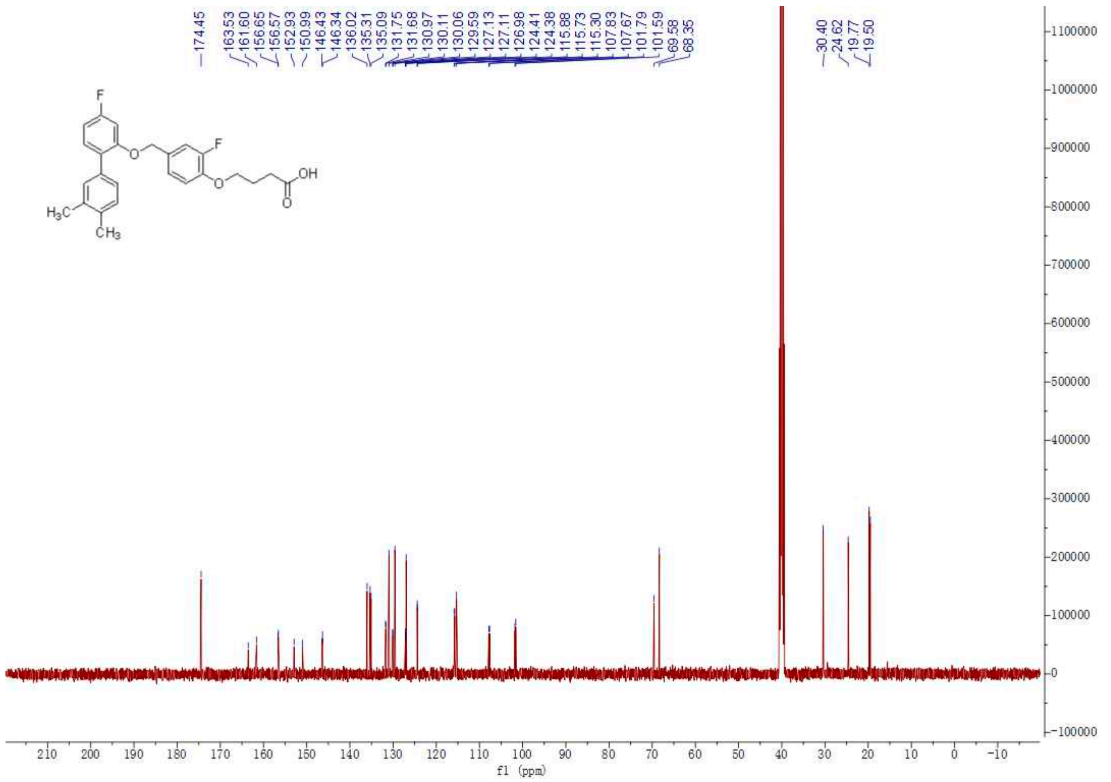

Figure S58. ^{13}C NMR spectrum **14g**.

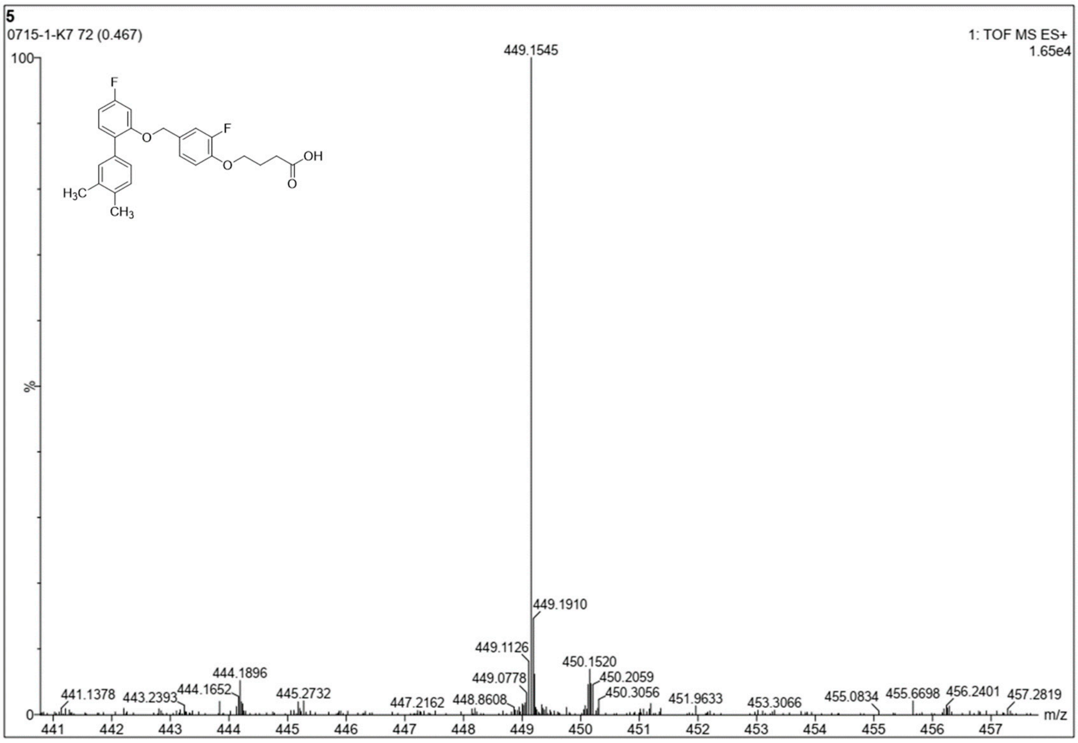

Figure S59. HRMS spectrum **14g.**

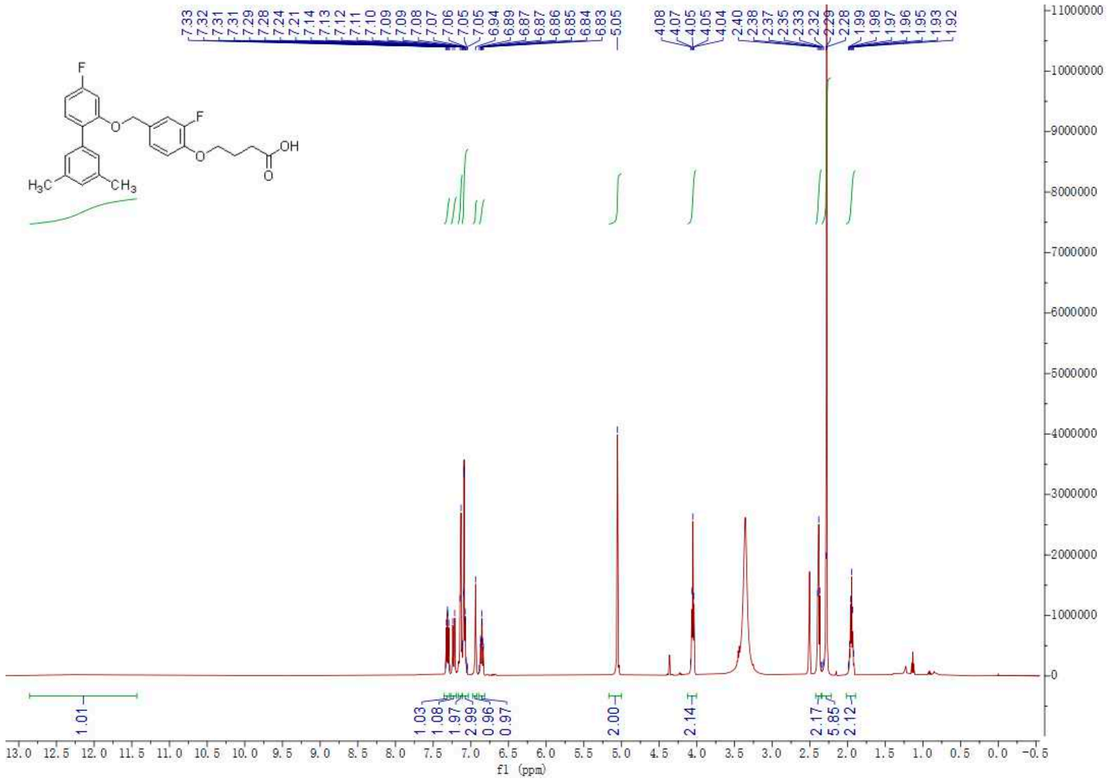

Figure S60. ^{1}H NMR spectrum **14h.**

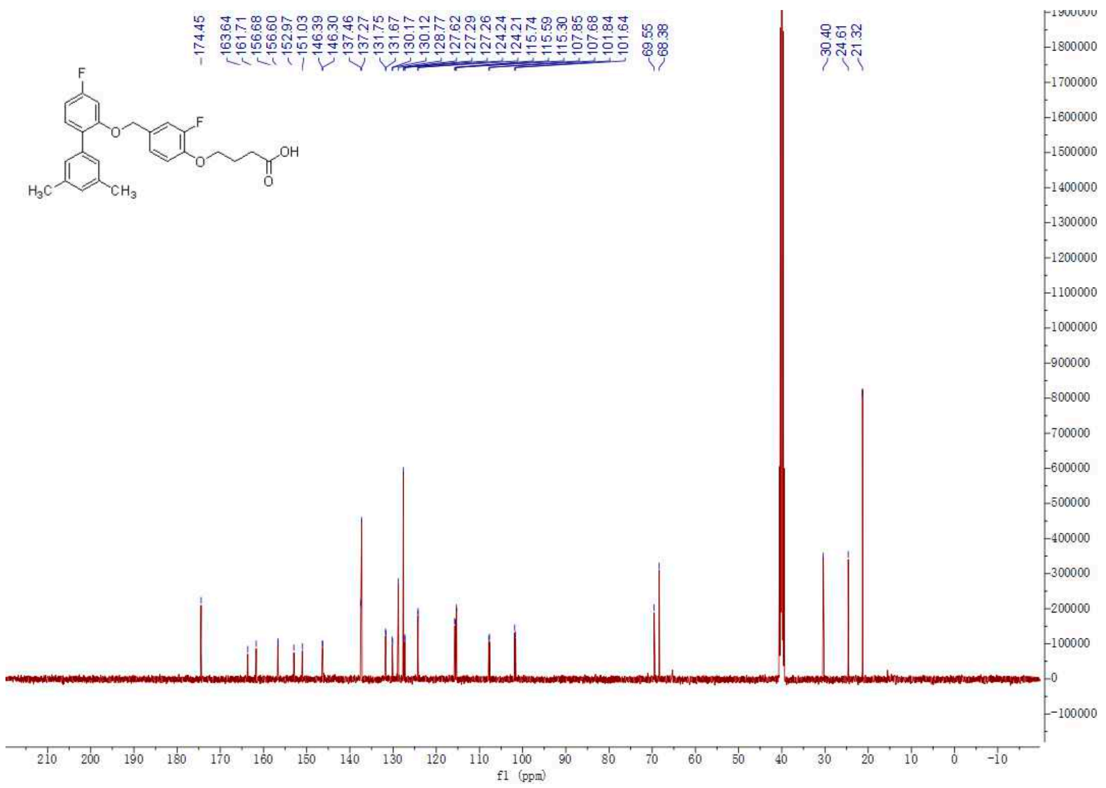

Figure S61. ^{13}C NMR spectrum **14h**.

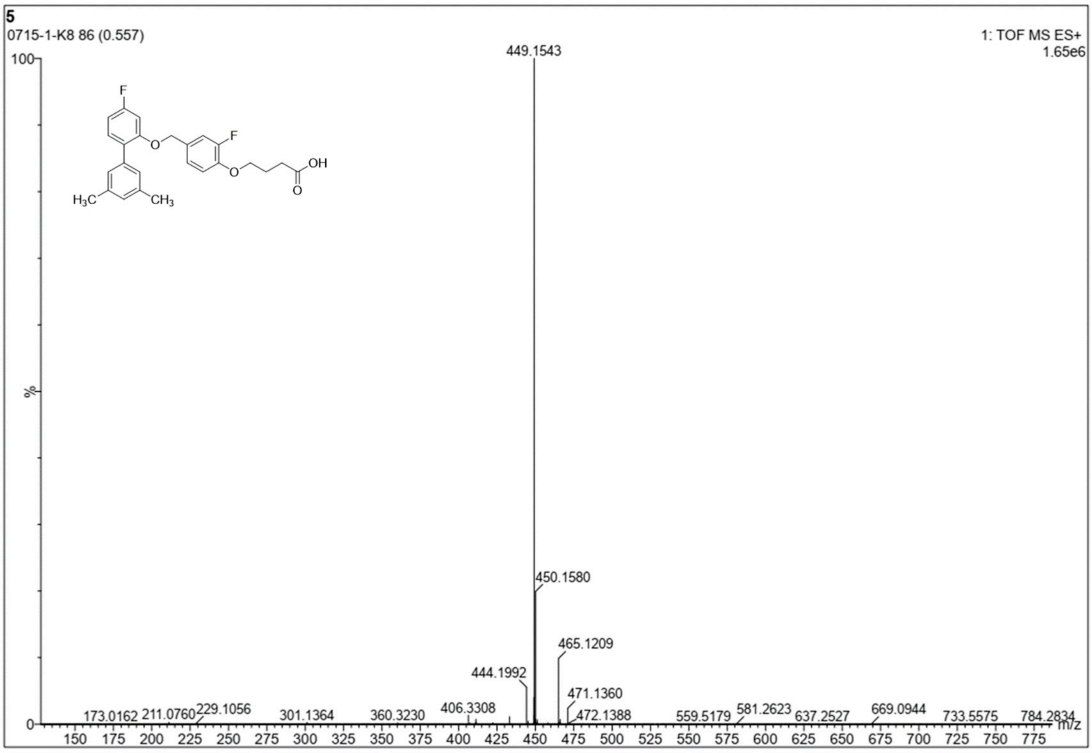

Figure S62. HRMS spectrum **14h.**

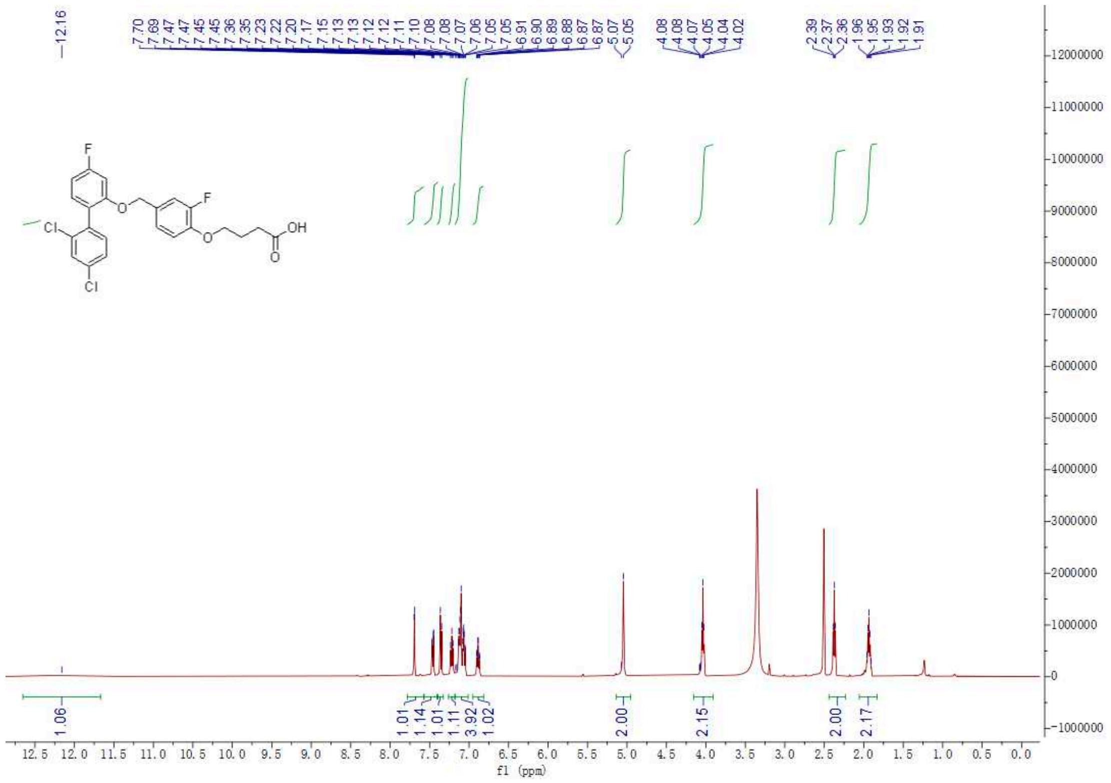

Figure S63. ^{1}H NMR spectrum **14i.**

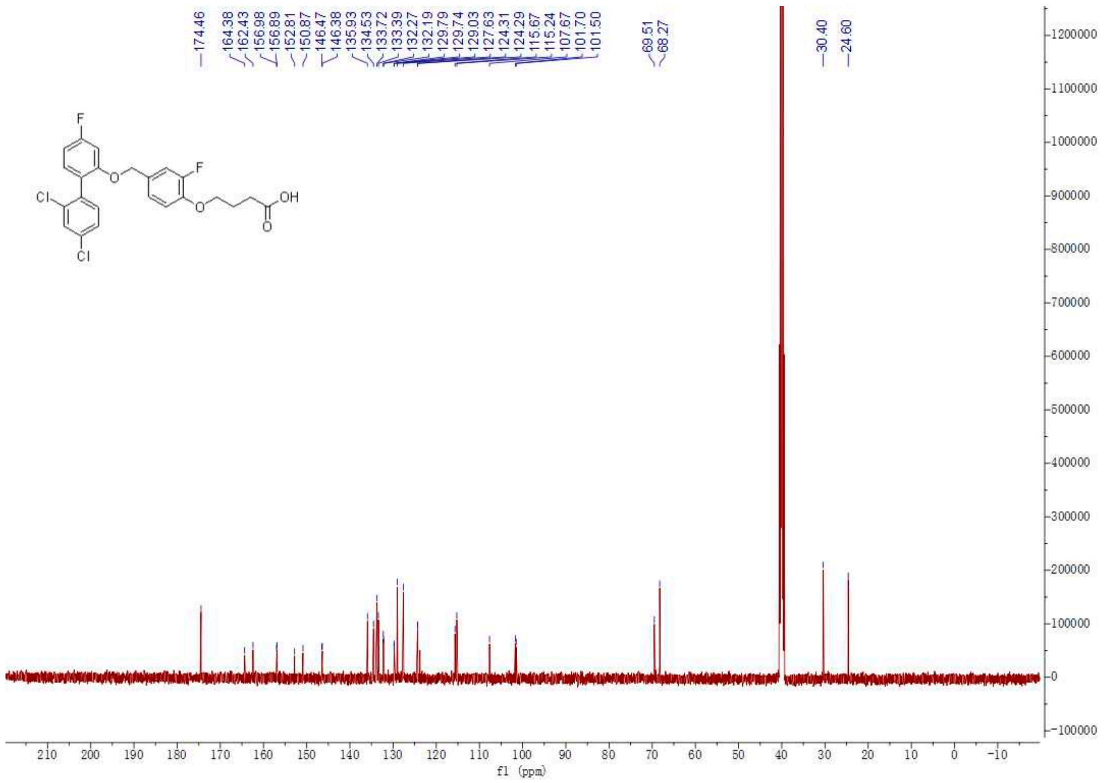

Figure S64. ^{13}C NMR spectrum **14i**.

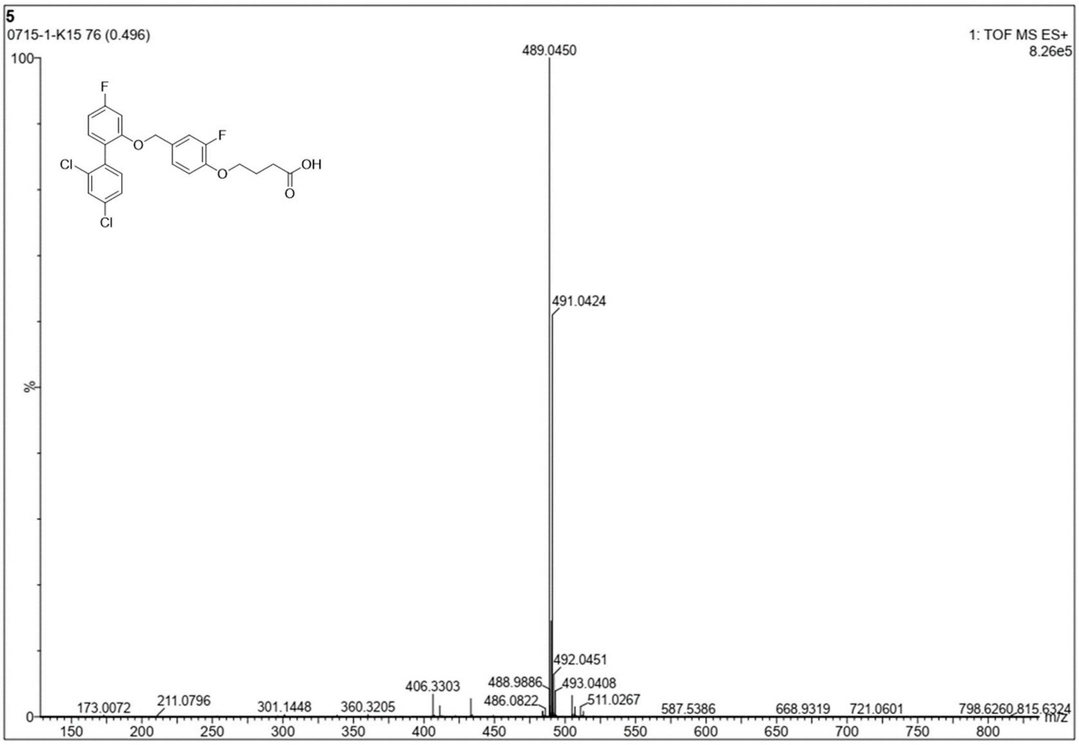

Figure S65. HRMS spectrum **14i.**

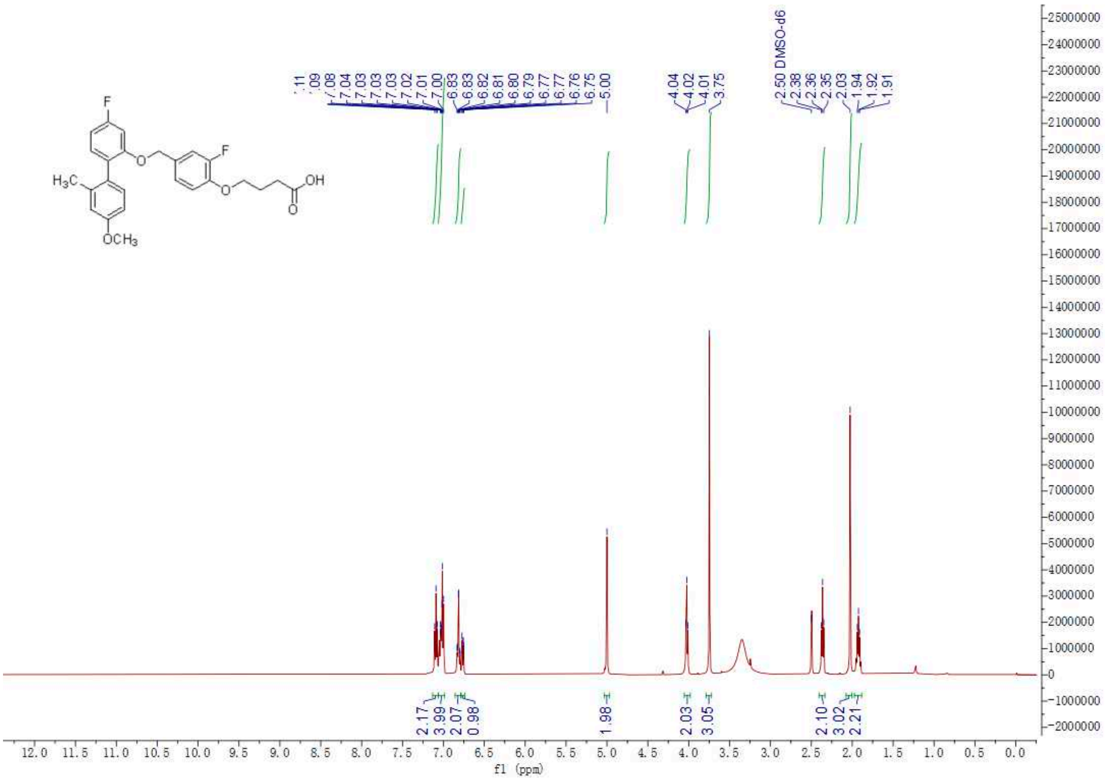

Figure S66. ^{1}H NMR spectrum **14j.**

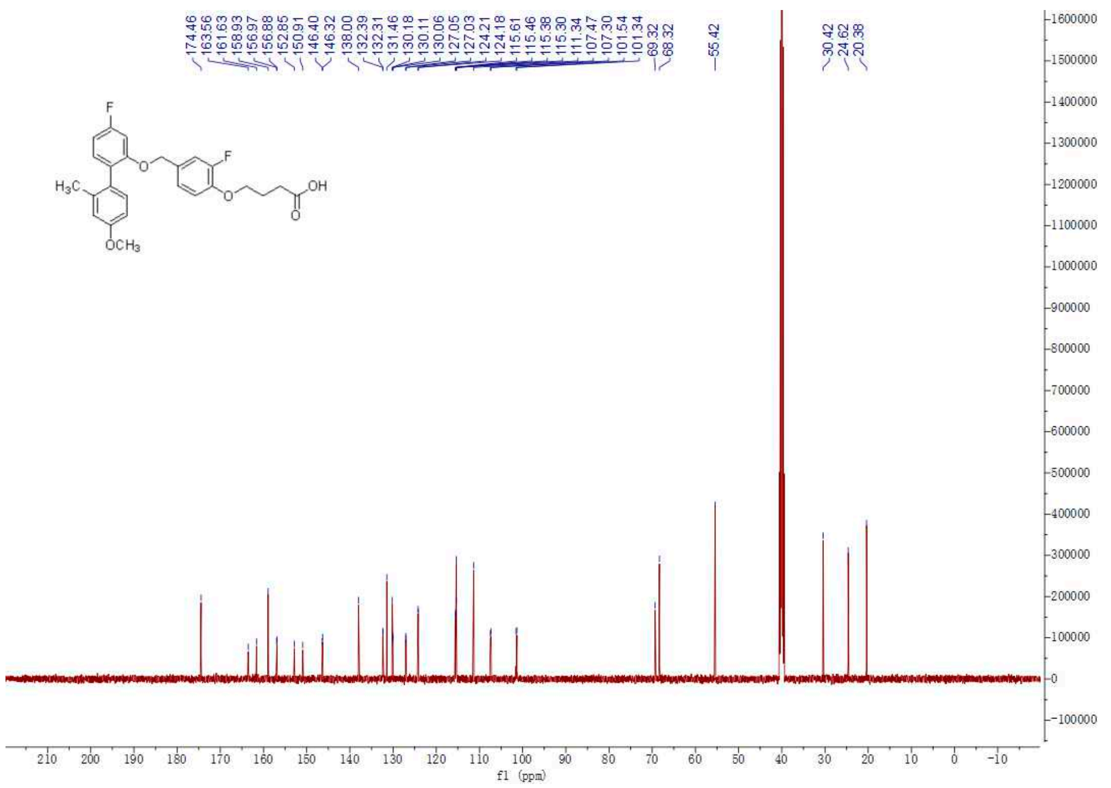

Figure S67. ^{13}C NMR spectrum **14j**.

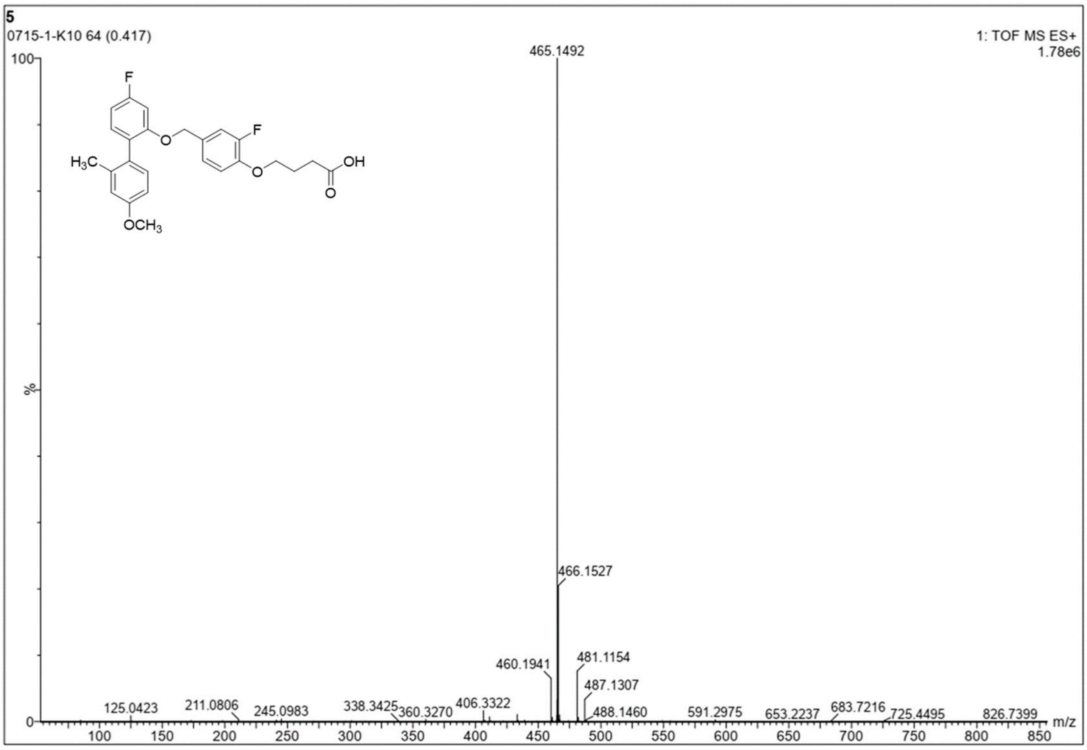

Figure S68. HRMS spectrum **14j.**

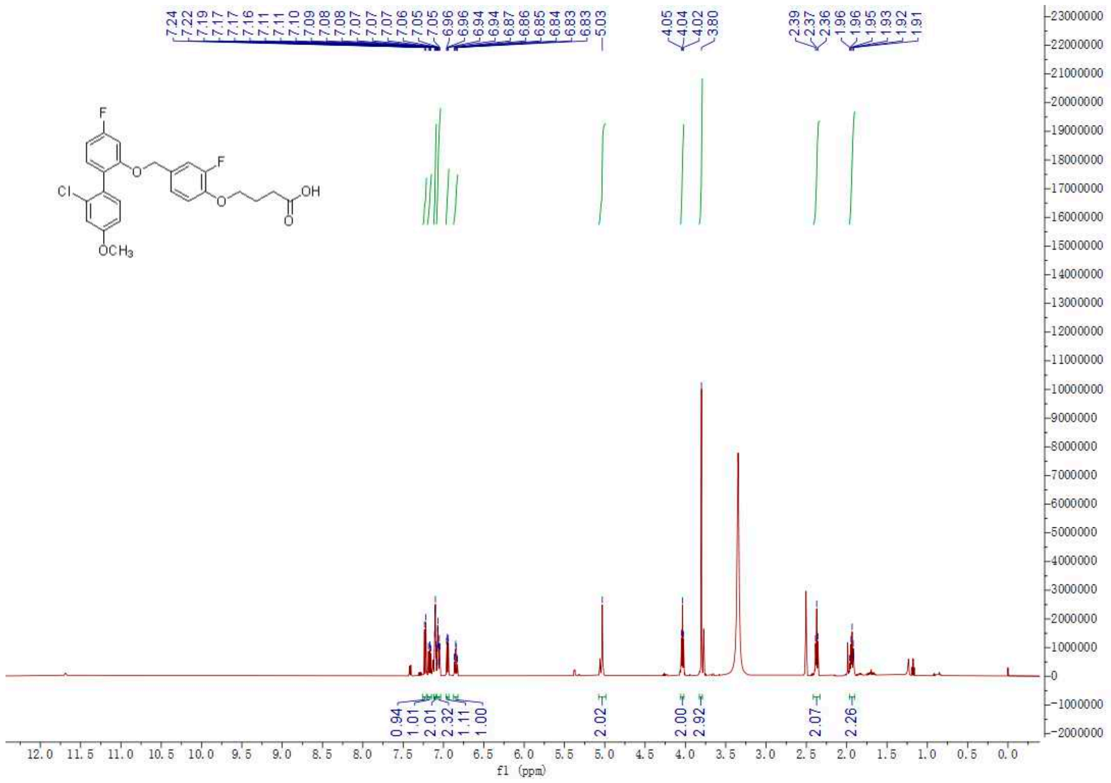

Figure S69. ^{1}H NMR spectrum **14k.**

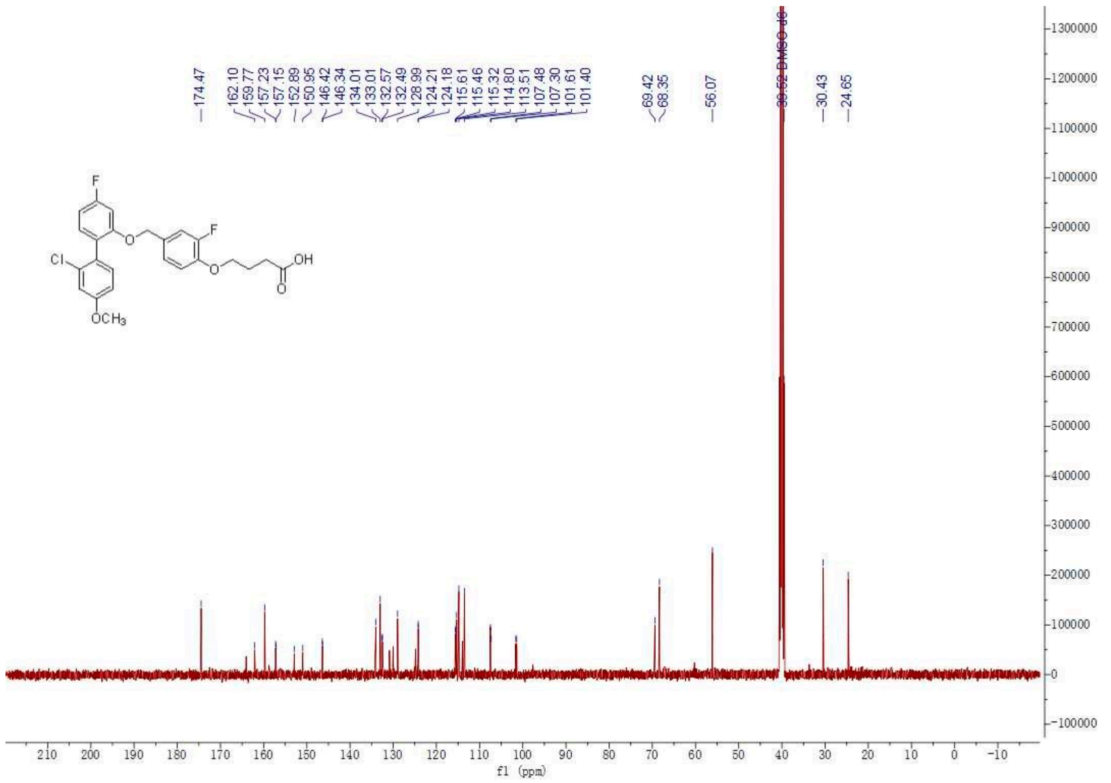

Figure S70. ^{13}C NMR spectrum **14k**.

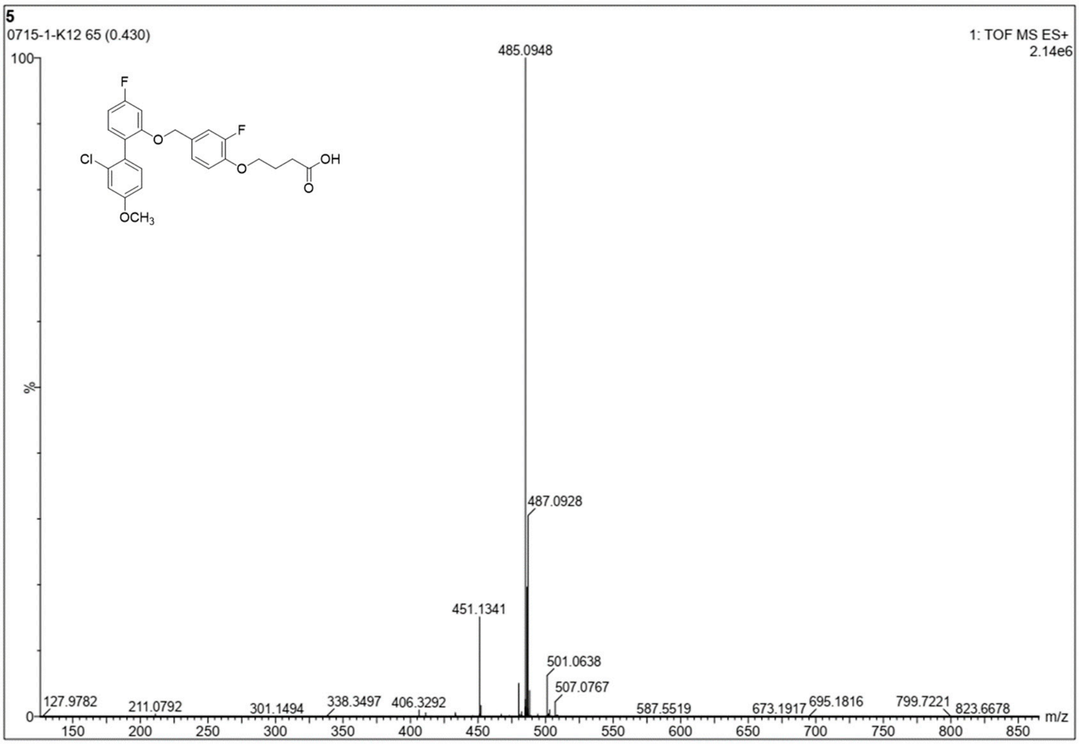

Figure S71. HRMS spectrum **14k.**

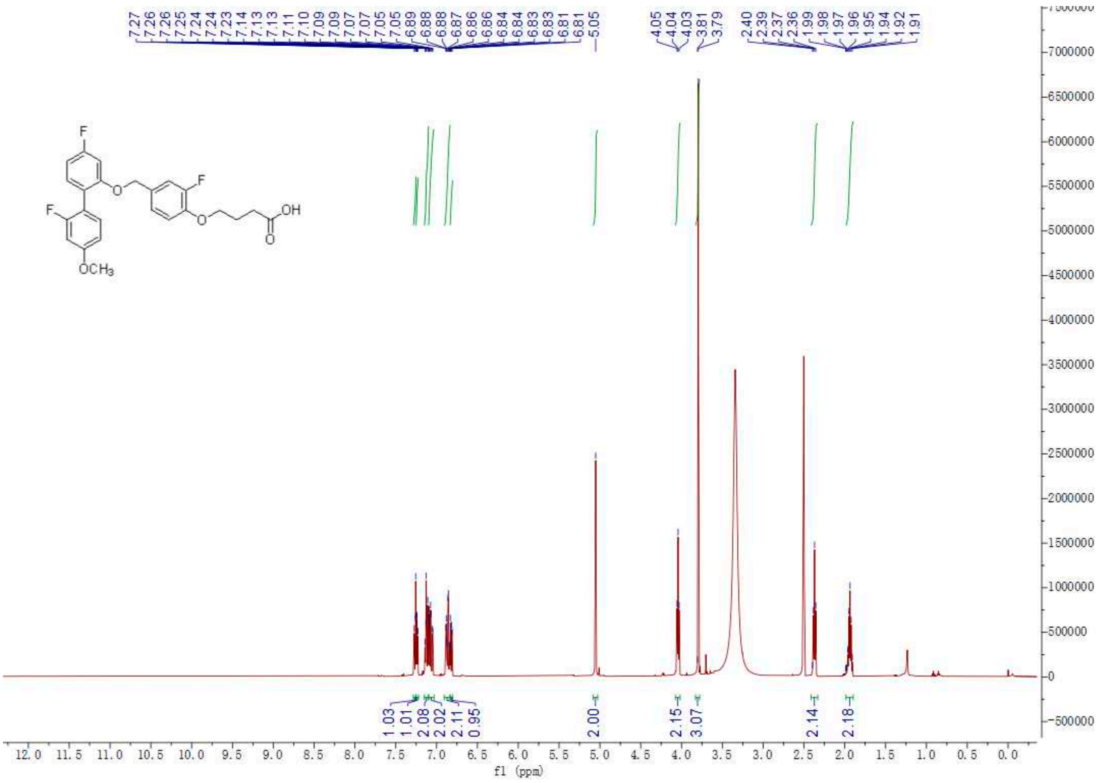

Figure S72. ^{1}H NMR spectrum **14l.**

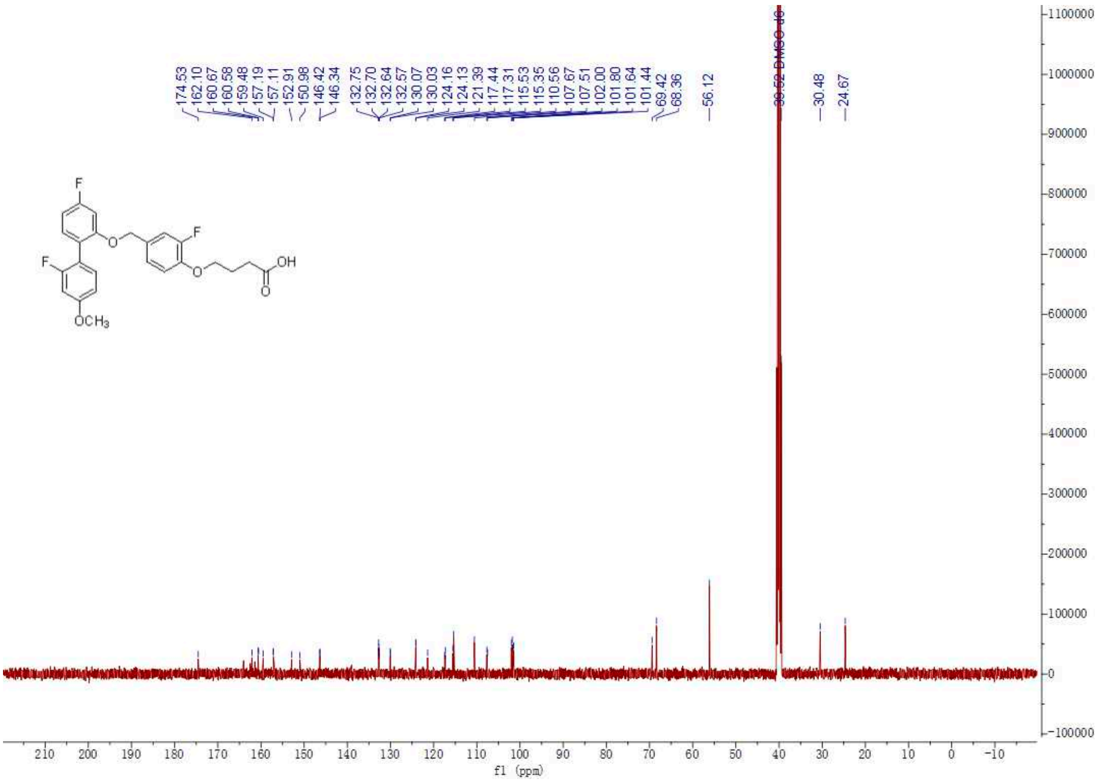

Figure S73. ^{13}C NMR spectrum **14l**.

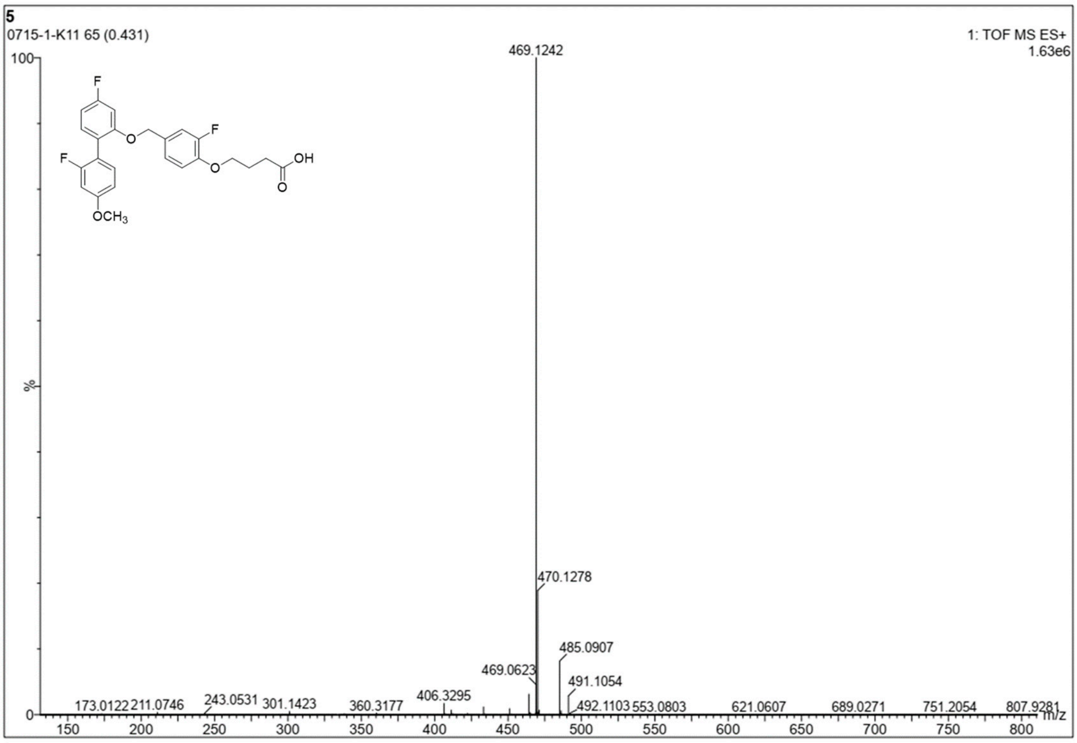

Figure S74. HRMS spectrum **14l.**

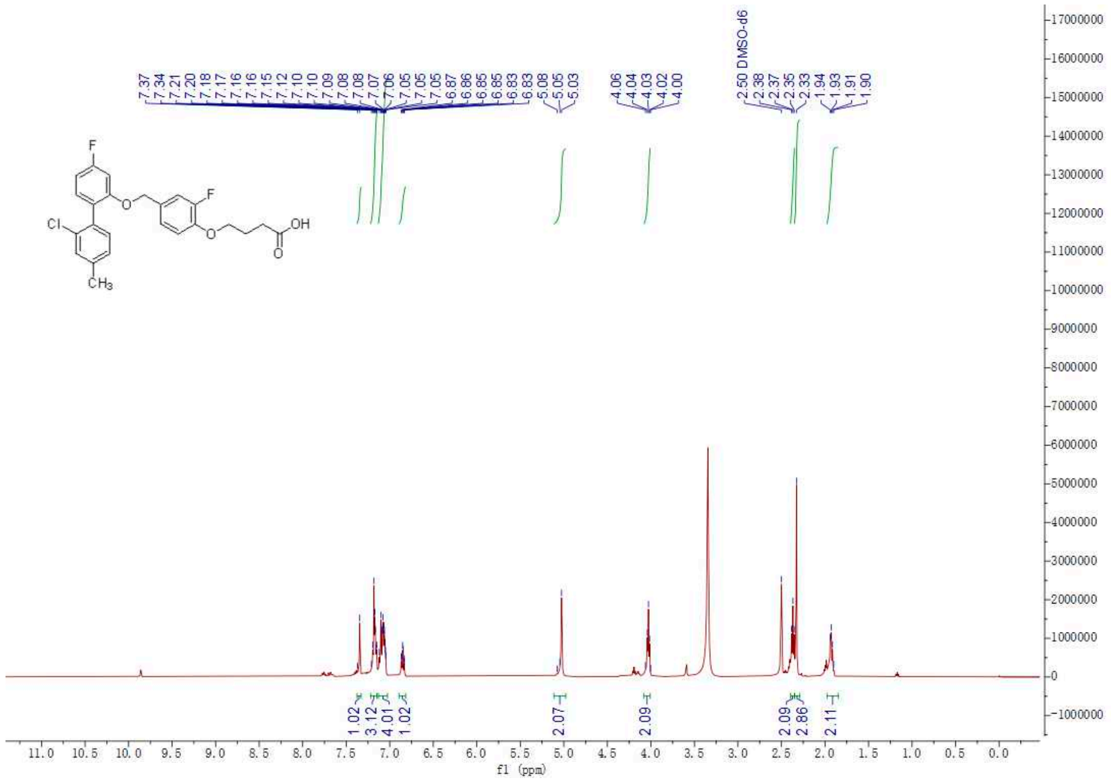

Figure S75. ^{1}H NMR spectrum **14m.**

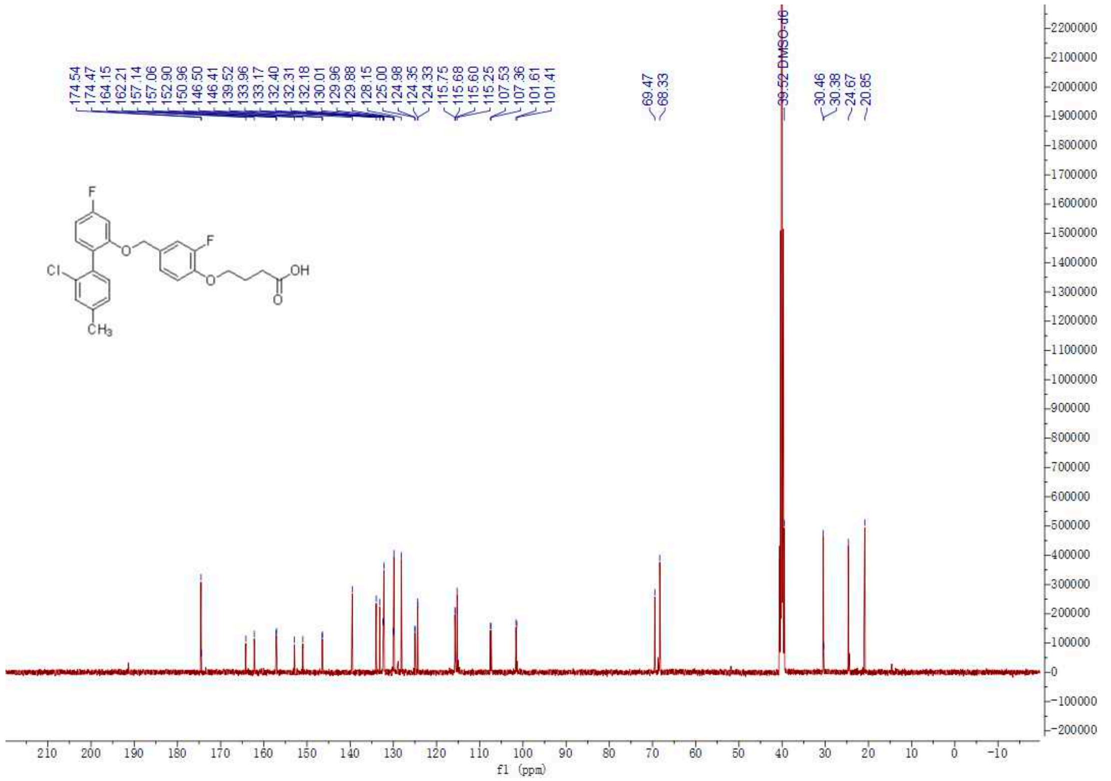

Figure S76. ^{13}C NMR spectrum **14m**.

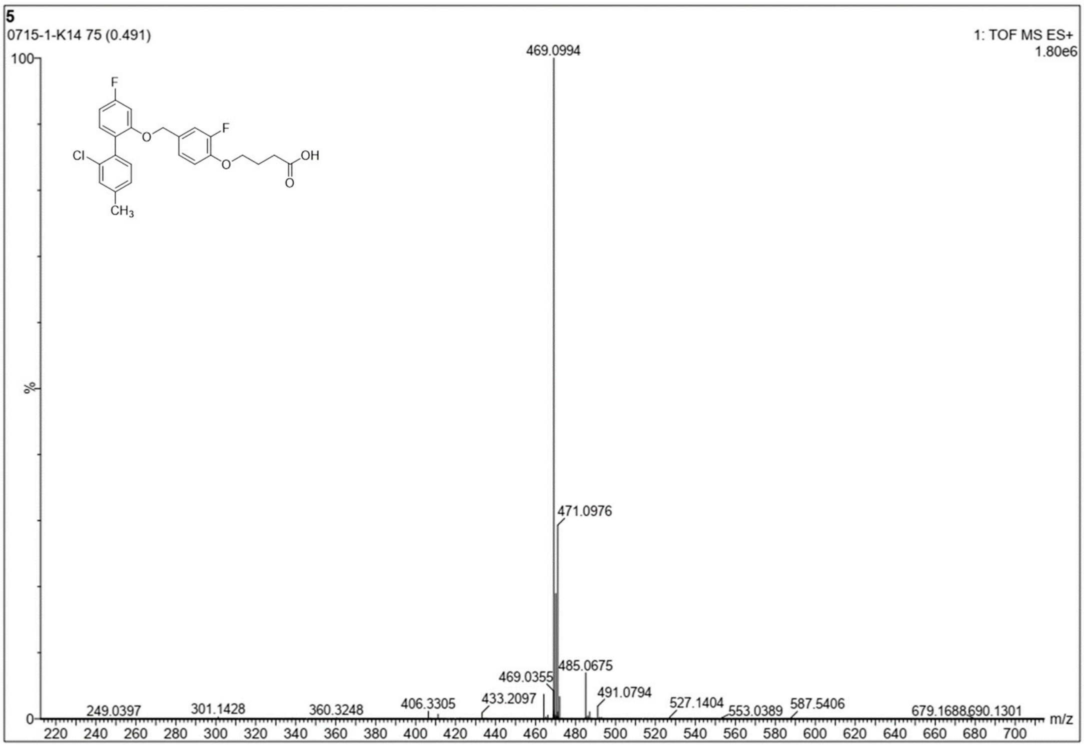

Figure S77. HRMS spectrum **14m.**

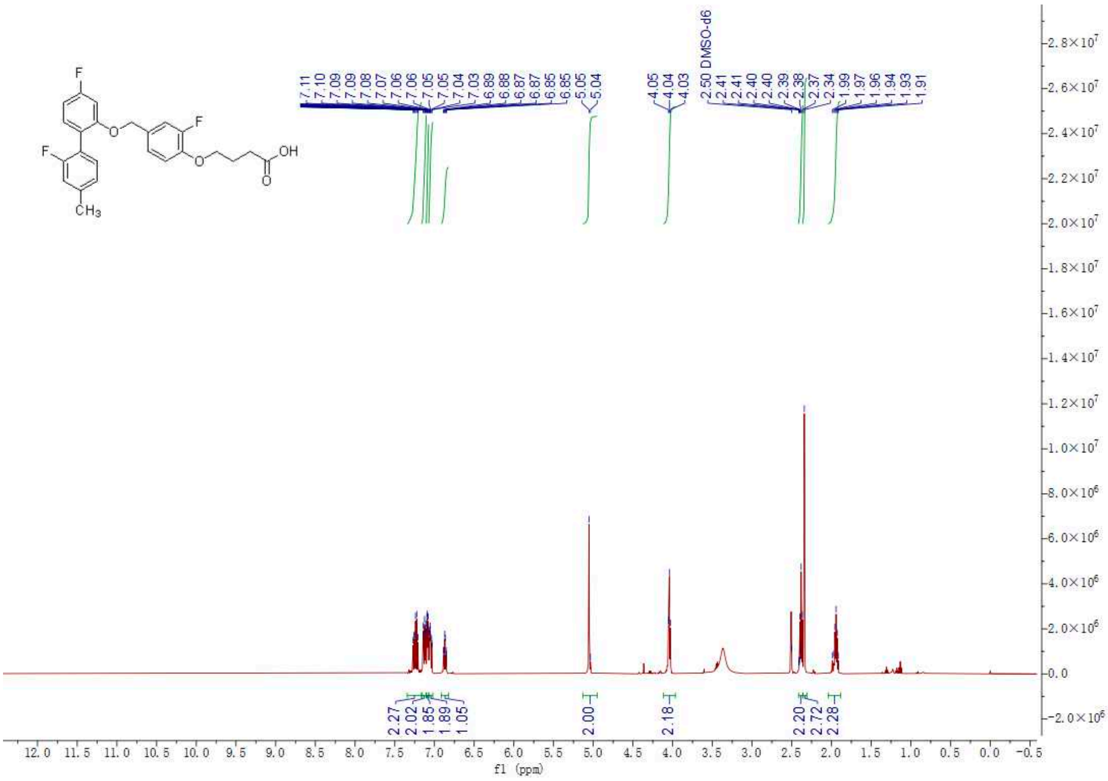

Figure S78. ^{1}H NMR spectrum **14n.**

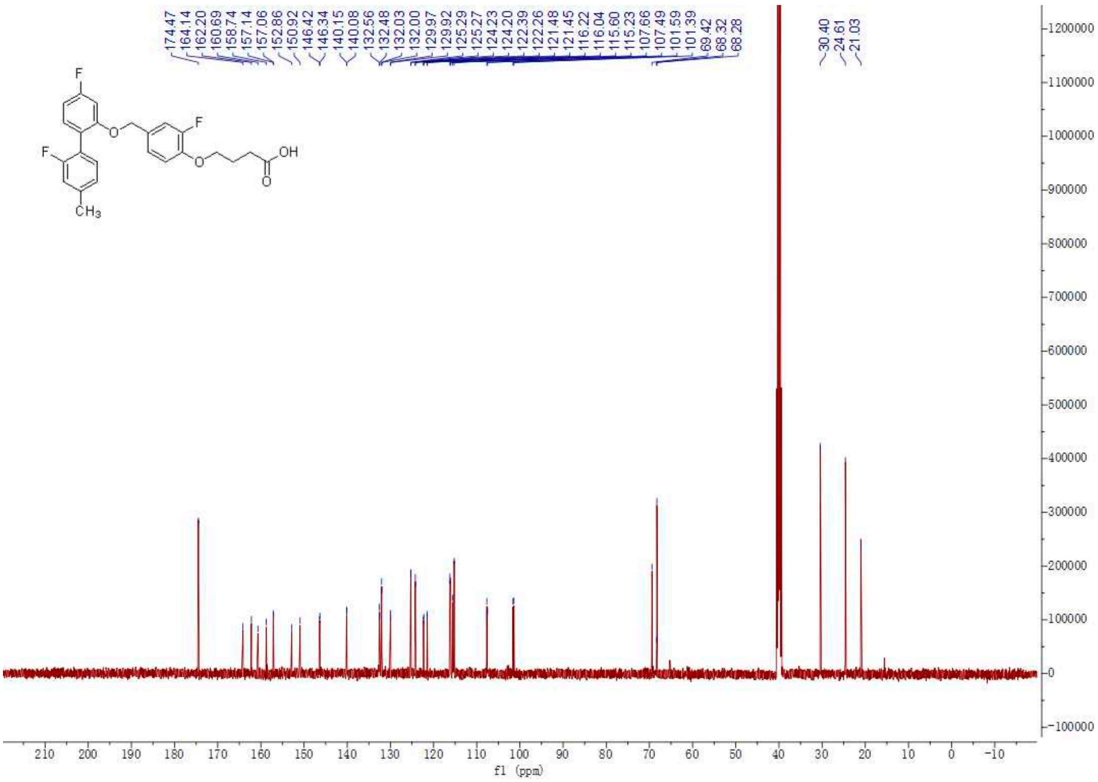

Figure S79. ^{13}C NMR spectrum **14n**.

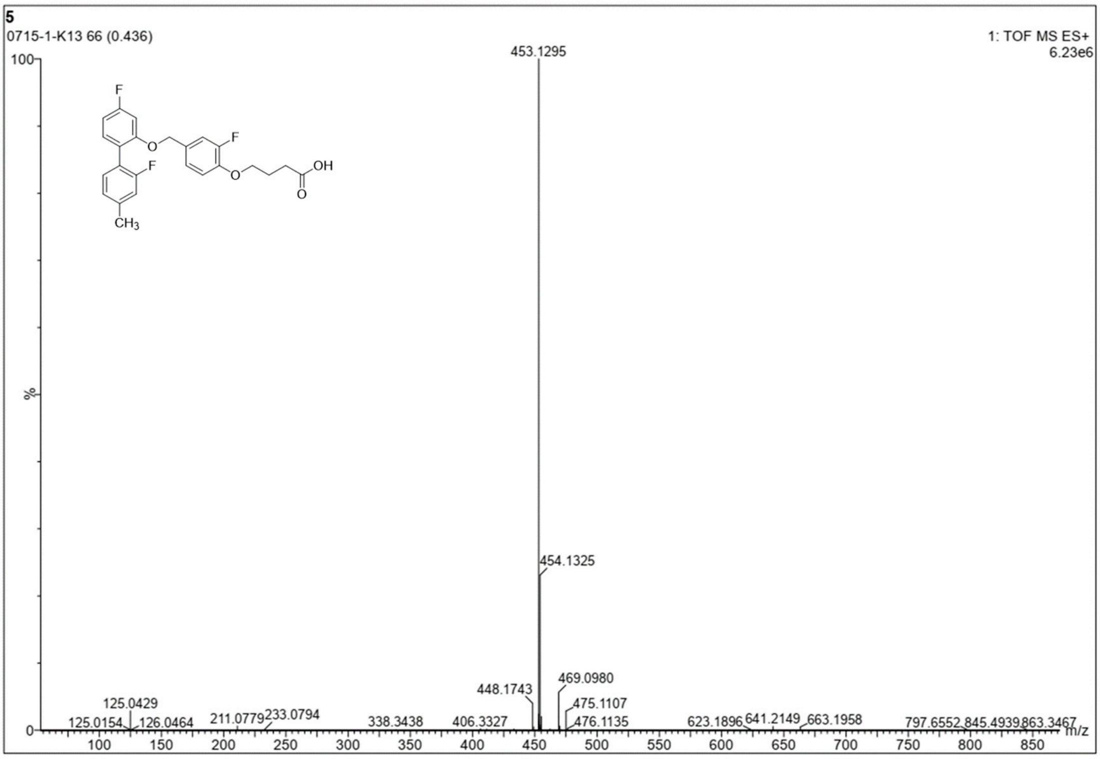

Figure S80. HRMS spectrum **14n.**
